# Point of Care Testing for Infectious Disease in Europe: A Scoping Review and Survey Study

**DOI:** 10.3389/fpubh.2021.722943

**Published:** 2021-10-20

**Authors:** Lucy Hocking, Jenny George, Eeva K. Broberg, Marc J. Struelens, Katrin C. Leitmeyer, Advait Deshpande, Sarah Parkinson, Joe Francombe, Katherine I. Morley, Helena de Carvalho Gomes

**Affiliations:** ^1^RAND Europe, Cambridge, United Kingdom; ^2^European Centre for Disease Prevention and Control, Stockholm, Sweden

**Keywords:** near patient, diagnostics, infectious diseases, Europe, point of care (POC) diagnosis, point of care (POC), near patient testing

## Abstract

**Background:** Point of care testing (POCT) for infectious diseases is testing conducted near the patient. It allows clinicians to offer the most appropriate treatment more quickly. As POCT devices have increased in accuracy and become more cost-effective, their use has grown, but a systematic assessment of their use for clinical and public health management of infectious diseases in EU/EEA countries has not been previously undertaken.

**Methods:** A scoping review of the literature on POCT in EU/ EEA countries as at November 2019, and a survey of key stakeholders.

**Results:** 350 relevant articles were identified and 54 survey responses from 26 EU/EEA countries were analysed. POCT is available for a range of infectious diseases and in all countries responding to the survey (for at least one disease). POCT is commonly available for influenza, HIV/AIDS, Legionnaires' disease and malaria, where it is used in at least half of EU/EEA countries. While POCT has the potential to support many improvements to clinical care of infectious diseases (e.g., faster diagnosis, more appropriate use of antimicrobials), the results suggest POCT is infrequently used to support public health functions (e.g., disease surveillance and reporting).

**Conclusion:** Although POCT is in use to some extent in all EU/EEA countries, the full benefits of POCT in wider public health functions have yet to be realised. Further research on barriers and facilitators to implementation is warranted.

## Introduction

Rapidly diagnosing infectious diseases is important to provide patients with the most appropriate treatment in a timely manner. Diagnosing infections quickly can also aid with detecting and controlling infection outbreaks, as evidenced with the COVID-19 pandemic (1). Point of care testing (POCT), defined as testing that is performed near or at the site of a patient with the result leading to possible change in the care of the patient', offers the ability to rapidly diagnose infectious diseases (2).

The availability and use of POCT has increased in recent decades (3). This is partly due to advances in POCT technology that have made devices more accurate, easier to use and more cost-effective (4–6). One main benefit of POCT is to make diagnosing infectious diseases accessible in more settings, particularly those which may not have skilled technical staff or adequate laboratory equipment (4–6). By making test results available quickly, POCT facilitates faster infection-specific treatment for the patient (5), reducing the likelihood of the disease's transmission and allowing a more rapid public health response such as isolation. This can also help avoid the prescription of inappropriate antimicrobials (7, 8) that can contribute to antimicrobial resistance (AMR).

Although POCT has benefits for infectious disease surveillance and diagnosis, its availability and use across Europe has not previously been investigated in detail. This study aimed to: (1) obtain an overview of the literature on availability and use of POCT for the 56 communicable diseases under EU surveillance in 2019–20 (9) to assess the status and trends in use of POCT in EU/EEA countries as of 2019–20, including the impact of POCT on clinical practise and key public health functions (e.g., disease surveillance, national reporting of infectious diseases or infection control).

## Methods

This study involved a scoping review and a survey of key stakeholders. In this article we report and synthesise key findings from the review and survey. This study was started before the COVID-19 pandemic so it did not collect information on use of POCT during the pandemic, although this topic warrants further investigation.

### Scoping Review

The aim of the scoping review was to collate evidence from the academic literature on POCT in EU/ EEA countries, to gain a high-level overview of evidence on the key characteristics of POCT devices and the diseases they aim to detect. We followed the approach specified in Preferred Reporting Items for Systematic Reviews and Meta-Analyses (PRISMA) extension for Scoping Reviews (PRISMA-SCR) checklist (10).

A literature search protocol was developed to include any POCT device, any EU/EEA country and all 56 infectious diseases under EU surveillance plus a small number of related issues such as AMR and nosocomial infections (see Supplementary Material). The scoping review was limited to studies published between 2014 and 2019 and used the ISO definition of POCT, excluding self-testing by patients. While the ISO definition does not use a fixed time period for which the test needs to provide a result, usually a test is classed as point of care if results are provided in 90 min or less (11), which is the time limit used for this study. The literature search protocol was peer-reviewed using the PRESS approach [Peer-Review of Electronic Search Strategies; (12)]. Four databases of peer-reviewed scientific literature were searched: PubMed; Embase; Cochrane Library; and Scopus.

The articles were screened against defined inclusion/exclusion criteria (see Supplementary Material), including a pilot screen of articles by all researchers. After the screening stage, we developed an extraction template in Excel based on the research questions (see Supplementary Material).

We used the statistical software package, R (13), to summarise categorical data using counts and percentages, and qualitative thematic analysis to identify common themes from open text data (14). The qualitative themes were developed iteratively, with an initial review of all the data to identify common themes, and then identifying commonalities amongst themes in order to further refine them. The extracted data were then reviewed again for potential inclusion in the list of themes.

### Survey

The aim of the online stakeholder survey was to identify the status and trends in use of POCT in EU/EEA countries.

The survey questions focused on a range of topics relating to POCT, including the availability and use of POCT in each country and the impact of POCT on public health (see Supplementary Material).

We invited 186 stakeholders covering all EU/EEA countries, including policymakers, clinicians, European-level association members, clinical scientists and microbiologists, infectious disease specialists, and representatives from national authorities and from microbiological societies. We invited at least two participants per country and allowed participants to suggest colleagues who could complete the survey to support geographical coverage.

Survey responses were summarised as counts and percentages using R software (13). Since a single individual may not possess complete knowledge of POCT for all infectious diseases within their country, we categorised responses as to whether respondents from a single country agreed or disagreed with each other. Where possible, the responses from within each country were compared to assess how closely they aligned with one another. Where there was contradiction or uncertainty between respondents from the same country, the survey results for that question were categorised as such. These were distinguished from those instances in which all survey respondents from a country agreed, whether in the form of a positive or a negative response. For countries from which we had no survey response, we sought an interview with a relevant expert instead (three additional interviews conducted with representatives from the Czech Republic, Lithuania and Poland) and undertook targeted online searches to supplement our findings.

## Results

### Literature Identified Through the Scoping Review

**Figure 1:**
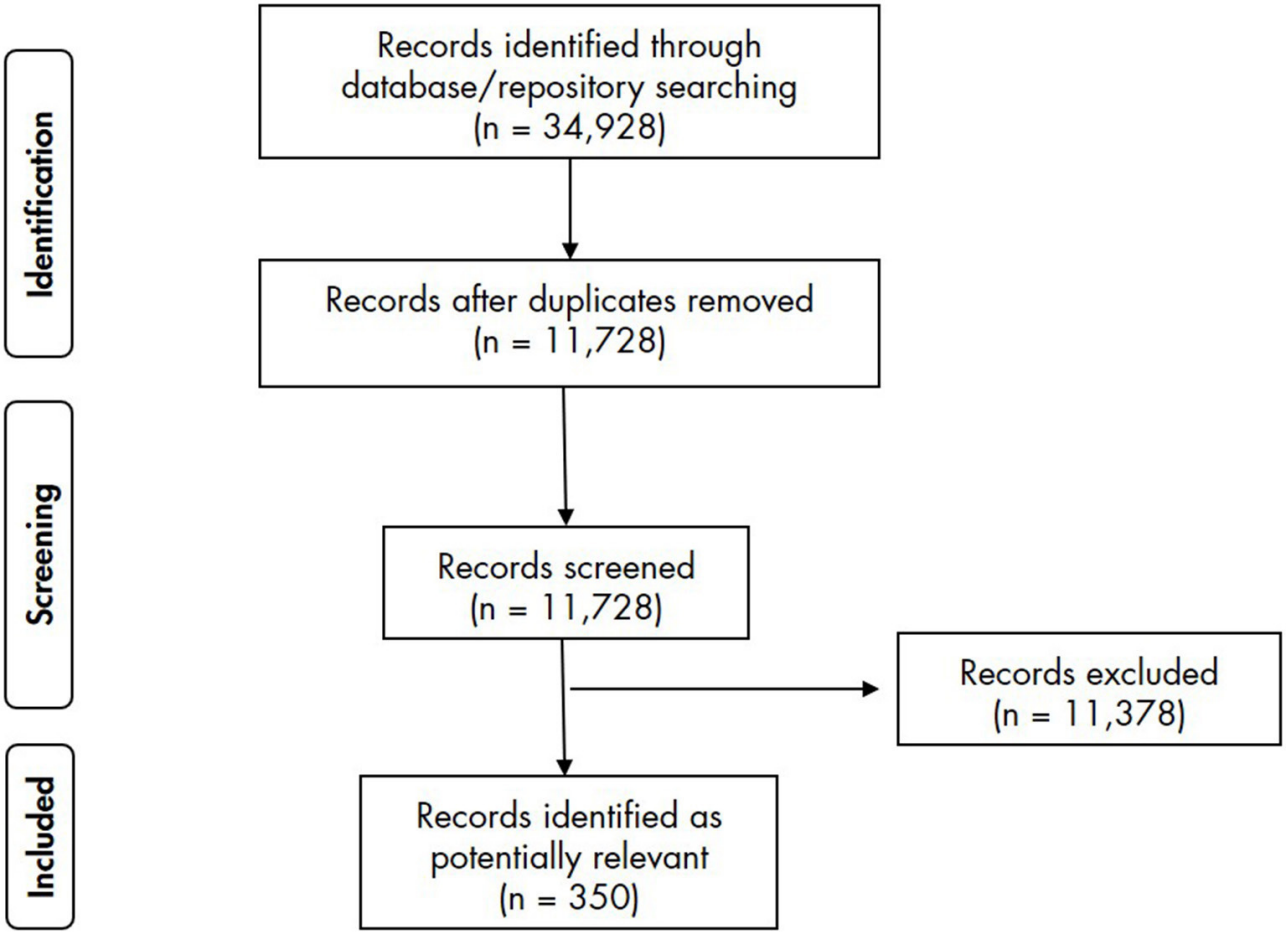
PRISMA-ScR flow diagram for the scoping review.

The literature search returned 11,728 results. Following screening against the inclusion/exclusion criteria, 11,378 articles were excluded and 350 were included and analysed (Figure 1).

### Survey Respondents

Fifty-four responses were analysed from 26 EU/EEA countries. The number of responses per country ranged from 1 to 7. No responses were received from Czech Republic, Hungary, Italy, Luxembourg and Portugal. Respondents came from a variety of professional backgrounds. The number of respondents for each role and length of experience are presented in Table 1. Respondents reporting they had “other” roles generally combined two of the above role descriptions such as clinicians and researchers.

**Table 1:** Survey respondent demographics.

**Country**	**Responses**	
Denmark	7	
Greece, Netherlands, Sweden	4	
Estonia	3	
Croatia, France, Germany, Ireland, Malta, Poland, Romania, Spain, Iceland, Norway, United Kingdom	2	
Austria, Belgium, Bulgaria, Cyprus, Finland, Latvia, Lithuania, Slovak Republic, Slovenia, Liechtenstein	1	
Czech Republic, Hungary, Italy, Luxembourg, Portugal	0	
**Role**	**Responses**	**% of respondents**
Microbiologist or other medical laboratory staff	23	43
Clinician or other healthcare professional	10	19
Academic/researcher	9	17
Regulator, policymaker, or government worker	8	15
Other	4	7
**Years of experience**	**Responses**	**% of respondents**
None	0	0
<1 year	1	2
1–5 years	8	20
6–10 years	7	17
11–15 years	11	27
16–20 year	0	0
21–25 years	6	15
26–30 years	4	10
30+ years	4	10

### The Availability and Use of POCT Devices for Diseases and Across EU/EEA Countries

The most commonly reported infection for which POCT was available, according to the survey, was influenza; for 19 countries (73% of those from which data were available), at least one respondent reported POCT was available. This was followed by HIV, reported by 17 countries (65%) and Legionnaires' disease and malaria [both reported by 13 countries (50%)]. For at least five countries (19% of countries) respondents reported that POCT is in routine clinical use for syphilis, chlamydia, hepatitis B, hepatitis C, nosocomial infections, AMR, tuberculosis, invasive pneumococcal disease, dengue, invasive meningococcal disease, gonorrhoea and cryptosporidiosis. Sometimes respondents from the same country gave differing answers, which may happen because a single individual may not have full knowledge of POCT use.

**Figure 2:**
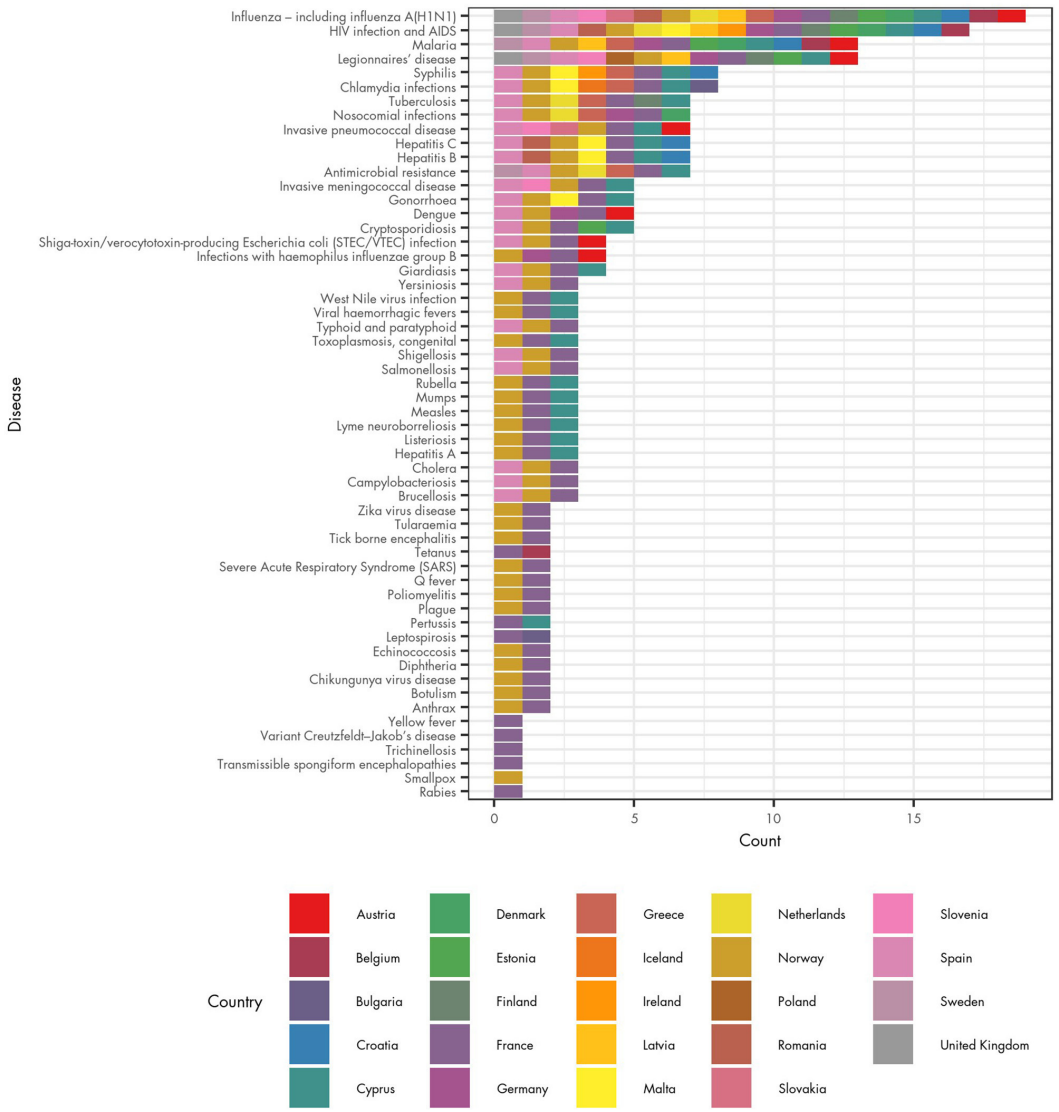
Availability of POCT for infectious diseases and associated health conditions in EU/EEA countries.

The survey also identified the EU/EEA countries in which POCT is available for the largest range of infectious diseases (Figure 2). Respondents from France reported the use of POCT for the largest number of diseases (55 diseases), followed by Norway (44 diseases). Respondents from Cyprus and Spain reported that POCT is in routine clinical use for 25 diseases, and Denmark reported POCT is used for seven infectious diseases. A further 8 countries reported that POCT was available for at least five infectious diseases: Austria; Germany; Greece; Sweden; Croatia; Malta; Estonia; and Netherlands.

The survey results should be treated with some caution. In France, a single survey respondent answered that POCT was used in clinical practise for 55 of the 56 communicable diseases under EU surveillance (with the exception being smallpox). In Norway a single respondent also reported POCT being used for many diseases. However, there are 18 diseases for which respondents from France and/or Norway were the only ones to report the use of POCT. The considerable difference between the number of diseases reported in these two countries and other countries may reflect incomplete knowledge or a different interpretation of what POCT is, rather than a comparable representation of the use of POCT in those countries.

The scoping review results provide some indication of where POCT is thought to be potentially useful. Outside of a laboratory setting, the most common settings for studies involving POCT were secondary care (47 studies), emergency departments (24 studies), clinics (e.g., sexual health and outpatient clinics; 33 studies), community (e.g., pharmacies, HIV checkpoints; 18 studies) and primary care (14 studies). Other settings were recorded for 15 studies.

### The Quality of POCT Devices Used in Europe

The scoping review indicated that a large number of different POCT devices were used in clinical research studies (with many studies discussing more than one type of POCT device). Many studies used a branded product name (e.g., Alere), but others were only described using generic terminology (e.g., rapid HIV test). Given the large number of tests, we categorised them based on how they were described and how often that description was used across studies. This resulted in identification of 447 brand-named tests that were mentioned in two or more studies. These devices were grouped into 72 categories according to their name (e.g., Alere, OraQuick etc. See Supplementary Material for the full table and description of tests). Due to the very high number of named devices mentioned, those that had a brand name but were only identified in one study were grouped into an “other” category. These categories were used to explore evidence on the diagnostic accuracy of devices and turnaround time (i.e., the time between conducting the test and receiving the results). The tests assigned a generic (non-branded) name were not included in this analysis as these devices are unlikely to be a homogenous group, so analysis of accuracy and turnaround time would not be meaningful.

### Turnaround Time of Test Results

Turnaround time for a POCT device may influence the practicality and feasibility of its use in different settings. The majority of studies did not include a measurement of turnaround time (for 379 tests), but of those that did, half of reported tests provided results between 10 and 29 min (145 tests). Turnaround time was reported as <10 min for 33 tests, 30–59 min for 53 tests and 60–90 min for 63 tests.

**Figure 3:**
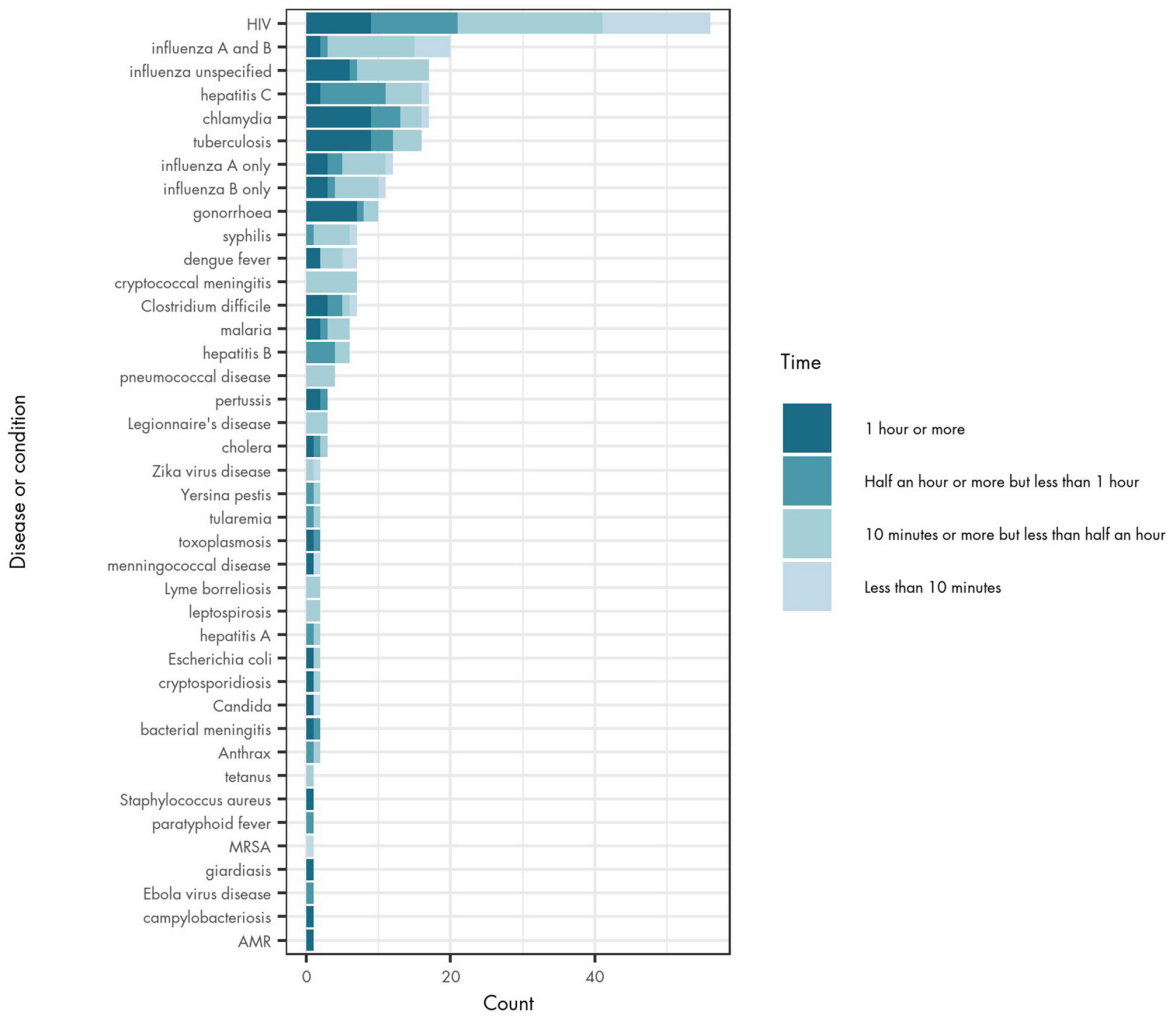
POCT turnaround time by type of infectious disease.

The reported turnaround time varied across different diseases (Figure 3), and also across different studies about the same infectious disease. In general, tests for syphilis, cryptococcal meningitis, pneumococcal disease, Legionnaire's disease, Zika virus infection, Lyme borreliosis, leptospirosis, tetanus and MRSA have turnaround times of <30 min. Chlamydia, tuberculosis (TB) and gonorrhoea have longer testing times (60–90 min), although the evidence on turnaround times is still mixed for these diseases. There were particularly large differences in turnaround time for both Candida species and meningococcal disease where some POCT devices reported results in <10 min and others in 60–90 min.

### Diagnostic Accuracy

For the 72 brand-named POCT devices identified from the scoping review, the available information on their clinical sensitivity and specificity (diagnostic accuracy) was explored. Many studies did not provide information on diagnostic accuracy but where it was included, there was considerable variation, including for the same tests. This may reflect the difficulties faced by the research team in grouping devices due to differences in test names used in the literature, differences in testing conditions, or changes over time to improve the accuracy of the tests. However, it is an important issue for further future research given the importance of diagnostic accuracy to the validity of diagnostics.

A total of 63 devices reported sensitivities of 90% or higher, but 57 devices reported sensitivity of <59%. The results suggest that the majority of named devices have relatively high specificity, with 126 devices reporting specificity of 99% or above.

### The Uses and Impact of POCT Devices in EU/EEA Countries

#### Clinical Impacts of POCT

Of the 350 articles reviewed in the scoping study, 69 discussed clinical impacts of POCT. We used qualitative thematic analysis to group these impacts into the following themes:

- More appropriate use of antimicrobials, which can lead to fewer unnecessary prescriptions and more effective and timely treatments (19 studies)
- Reduced time to diagnosis and/or treatment compared with traditional laboratory testing (18 studies).
- Reduction in hospital admissions, length of stay and/or emergency department waiting times (15 studies)
- The ability to test more people (14 studies)
- Reduction in healthcare costs (nine studies)
- More appropriate infection control measures (seven studies)
- Prevention of infectious disease transmission (seven studies)
- Fewer medical tests needed for a patient (six studies)
- Improved care linkage and patient management (six studies)
- Reductions in patient morbidity and/or mortality (six studies)
- Improved treatment adherence by the patient (three studies)

The majority of clinical impacts reported in the articles were positive. Only two studies noted negative impacts, which both related to economic aspects of the tests. A small number of studies reported that POCT did not have any impacts (14 studies).

#### Public Health Uses of POCT

Respondents to the survey reported that other than diagnosis, POCT was not widely used for public health functions such as disease surveillance, national reporting of infectious diseases or infection control. Nevertheless, disease surveillance was the public health function for which the greatest number of countries reported POCT use; reported by seven countries (Belgium, Bulgaria, Cyprus, Finland, Latvia, Slovakia and Slovenia). Respondents from six countries reported that POCT results are used for national reporting of infectious disease surveillance (Belgium, Bulgaria, Finland, France, Slovakia and Slovenia), while five countries reported that POCT results are used in national surveillance systems (Belgium, Bulgaria, Cyprus Finland and Slovenia). For AMR, there was no country in which all respondents agreed that POCT was used for monitoring such resistance, although our scoping review findings did suggest that POCT may be used for improving the appropriateness of antimicrobial prescriptions. There was also considerable uncertainty among respondents about the role of POCT in wider public health functions.

#### The Extent to Which POCT Has Replaced Traditional Diagnostic Tests

The survey respondents reported that overall, POCT only infrequently replaced traditional diagnostic tests. When exploring the results by country, the impact of POCT appears to be most significant in Spain, where respondents reported that POCT has replaced traditional tests for 14 infectious diseases (AMR; campylobacteriosis; chlamydia infection; cholera; cryptosporidiosis; giardiasis; hepatitis B; hepatitis C; HIV/AIDS; salmonellosis; shiga-toxin/verocytotoxin-producing *Escherichia coli* infection; shigellosis; typhoid and paratyphoid; and yersiniosis). There are only seven other countries where POCT was reported to have replaced other tests for at least one infectious disease: Slovenia, Austria, Cyprus, Norway, Belgium, Denmark and Sweden.

The number of diseases for which POCT has replaced other forms of test appear to be low (see Supplementary Material). For chlamydia, HIV and Legionnaires' disease, two countries (different countries in each case) reported that POCT had replaced other tests. For most other diseases, respondents from a majority of countries reported that POCT has not replaced other tests. For example, of 17 countries reporting that POCT is in routine use for HIV, 13 reported that the test did not replace existing diagnostic methods. Other diseases for which most countries reported no replacement of existing tests include Legionnaires' disease, hepatitis B, hepatitis C, invasive pneumococcal disease, syphilis and tuberculosis. Responses relating to influenza were very mixed, indicating uncertainty.

## Discussion

Amongst the diseases under EU surveillance, POCT is more widely available for infectious diseases with a higher incidence, such as influenza and HIV (15), but also for some diseases which are less common in Europe, such as Legionnaire's disease and malaria. Explanations for having POCT for diseases which are less common in Europe may vary by disease. One possible explanation for Legionnaires' disease may have been the rapid increase in reports of this disease in Europe between 2014 and 2018 (16) as well as the established clinical benefit of rapid administration of appropriate antimicrobial therapy in this condition (17). POCT for malaria may have been influenced by increases in refugee and migrant populations from areas where malaria is endemic (18) or due to Europeans travelling to and from such countries.

The reasons for differences between the availability of POCT by country and by disease were not clear. However, one study indicates that a possible factor behind the high use of POCT in France may be the decision to use fewer centralised laboratories, which creates a gap in diagnostics that POCT could fill (19).

The scoping review identified studies that were conducted in a range of healthcare settings, as well as in the community. This demonstrates the potential scope of POCT to diagnose infectious conditions in hard-to-reach communities and in vulnerable groups who would benefit from community testing. However, the reported turnaround times of some POCT devices may preclude their use in such settings. The most common reported turnaround time was 10–29 min, but few provided results in <10 min. Devices where results take longer than 10 min may not be optimal in some settings, e.g., where appointments are short or patients may not wait for results. The scoping review indicated both variation in, and uncertainty over, turnaround times for POCT devices in the literature. This may be due to factors such as different types of tests being available for one disease, improvements to POCT devices between 2014 and 2019, or due to the variation in design of different studies. There would be merit to further research on this issue.

In addition to turnaround time, indicators of diagnostic accuracy (sensitivity and specificity) demonstrated mixed results, with considerable variation in tests for the same infectious disease or even the same device. This may reflect the difficulties faced by the research team in categorising devices across varied references in the literature, bias related to differences in the quality of studies, differences in testing conditions/populations, or improvements in the accuracy of the tests over time. However, it is an important issue for further future research through systematic review and meta-analysis given the importance of diagnostic accuracy to the validity of a test.

Supporting public health functions is a potential gap in which POCT could provide benefit, particularly for screening asymptomatic chronic infection in at-risk groups and for identifying carriers of transmissible AMR, which is a key threat to global health (20). However, survey respondents reported that POCT was used very little for key public health functions, outside of diagnosis. No respondent reported POCT being used for monitoring antibiotic resistance although the reviewed literature suggests that POCT may be used for improving the appropriateness of antimicrobial prescriptions by detecting virus infections, reducing the number of unnecessary prescriptions and providing disease-specific treatment.

While the reasons behind the lack of replacement of traditional diagnostics were not explored in the survey, it may be that POCT devices are more likely to be introduced in a setting where diagnostics were not previously available (such as community settings). This has been seen with COVID-19, in which the European Commission recommended the use of rapid tests in certain community settings, such as for social care settings, long-term care facilities, food processing settings and for cross-border travel (21). Alternatively, POCT may be used as an additional diagnostic test, possibly to initially screen patients that need additional laboratory tests to confirm results from POCT, as has been seen with COVID-19 testing. This is a potential gap in which POCT could provide benefit, particularly as AMR is increasing. Other frequently mentioned impacts of POCT include quicker time to diagnosis than traditional diagnostic methods, improved flow in hospitals and increased testing rate.

### Study Strengths and Limitations

This study provides a broad overview of the evidence base and expert insights into the use, availability, quality and impact of POCT across the EU/EAA countries for infectious diseases. In particular, 350 studies were included in the scoping review which provides a breadth of evidence on different POCT devices. In addition, representatives from 26 EU/EEA countries responded to the survey.

However, unlike a systematic review, a scoping review does not seek to critically appraise the literature, assess the quality of the evidence or provide a synthesised response to research questions. This scoping review was restricted to European countries and English language text and therefore will not have identified all relevant studies. In addition, the discussion of POCT in academic literature does not mean that the device is in routine clinical use for a particular infectious disease or in a particular country.

The survey was undertaken during the escalation of the COVID-19 pandemic which significantly affected the number of survey respondents. We received no responses from five countries and only one response each from a further 10 countries. A single response from each EU/EEA country would theoretically be sufficient if the respondent had complete knowledge of POCT across all diseases but this is unlikely to be the case. For countries where we had only one respondent, we may not have captured full information.

Since a single individual may not possess complete knowledge of POCT in their country where there were multiple respondents from a single country, for some countries we obtained contradictory evidence. This has implications for undertaking a full analytical interpretation of the results. We compared responses from within each country to assess how closely they aligned and where there was contradiction or uncertainty, and the survey results were categorised as such. In addition, the respondents' interpretations of the survey questions may have differed. We defined POCT using the ISO definition, but results from some countries suggest that survey respondents may have interpreted characteristics of POCT in different ways.

### Conclusion

This study has compiled valuable insights into how and where POCT is used in Europe. However, it has also highlighted several gaps in information and apparent variation between its use for different diseases and between countries. This study highlights how further research could support better understanding of the use and impacts of POCT including the role of POCT promoting public health and reasons for variation in the use of POCT across EU/EEA countries.

For many years, POCT has been applied to detect and diagnose infectious diseases quickly, close to the patient- healthcare interface. The COVID-19 pandemic has underlined the potential uses of POCT devices on a large scale to detect infectious diseases and for public health risk management. POCT is already available for COVID-19 [22] so it would be valuable to update this study to include COVID-19 POCT devices, their potential usefulness and public health effectiveness in Europe.

## Data Availability Statement

Raw Survey Data is unavailable due to confidentiality/data protection regulations. Requests to access the datasets should be directed to Lucy Hocking, lhocking@randeurope.org.

## Author Contributions

LH, JG, AD, SP, and JF had input to the study protocol, survey questions, scoping review, and manuscript preparation. KM assisted with the survey analysis and conducted a critical review of the manuscript. EB, MS, and KL conceptualised the idea for the work, had input to the study protocol, survey questions, contributed to interpretation of results, and critical review of the manuscript. EB was also the project manager. HC was the project owner and conducted a critical review of the manuscript. All authors contributed to the article and approved the submitted version.

## Funding

This study was funded by the European Centre for Disease Prevention and Control.

## Conflict of Interest

The authors declare that the research was conducted in the absence of any commercial or financial relationships that could be construed as a potential conflict of interest.

## Publisher's Note

All claims expressed in this article are solely those of the authors and do not necessarily represent those of their affiliated organizations, or those of the publisher, the editors and the reviewers. Any product that may be evaluated in this article, or claim that may be made by its manufacturer, is not guaranteed or endorsed by the publisher.
