# Supplementary material for: Point of Care Testing for Infectious Disease in Europe: A Scoping Review and Survey Study
Source: Front Public Health. 2021 Oct 20;9:722943. doi: 10.3389/fpubh.2021.722943 (PMC8563586; doi:10.3389/fpubh.2021.722943)
Supplement: Supplementary file 1 [file Data_Sheet_1.docx]

Supplementary Material

# Search protocol

## PubMed search strings and number of search hits

### 56 diseases

**Date Limitation:** 2014-present

This search was run on 15 November 2019.

| Search strings | No of hits |
| --- | --- |
| #1 Point of care test*[tw] OR POCT[tw] OR rapid diagnostic test*[tw] OR rapid test*[tw] OR bedside test*[tw] OR near patient test*[tw] OR handheld device*[tw] OR handheld instrument*[tw] OR portable test*[tw] OR portable device*[tw] OR portable instrument*[tw] OR bedside computing[tw] OR Infectious disease testing[tw] OR infectious disease screening*[tw] OR point of care technolog*[tw] OR bedside technolog*[tw] OR point of care system*[tw] OR point-of-care systems[MeSH] OR point-of-care testing[MeSH] | 12821 |
| #2 Anthrax[tw] OR anthracis[tw] OR "Anthrax"[Mesh] OR "Bacillus anthracis"[Mesh] | 1804 |
| #3 #1 AND #2 | 13 |
| #4  botulism[tw] OR botulinum[tw] OR botulism[Mesh] OR “Clostridium botulinum”[Mesh] | 6346 |
| #5 #1 AND #4 | 11 |
| #6 brucellosis[tw] OR Brucella[tw] OR b. abortus[tw] OR b.canis[tw] OR b. melitensis[tw] OR b.ovis[tw] OR b. suis[tw] OR brucellosis[Mesh] OR Brucella[Mesh] | 2909 |
| #7 #1 AND #6 | 19 |
| #8 Campylobacteriosis[tw] OR campylobacter*[tw] OR c.coli[tw] OR c.fetus[tw] OR c.hyointestinalis[tw] OR c.jejuni[tw] OR c.lari[tw] OR c.rectus[tw] OR c.sputorum[tw] OR c.upsaliensis[tw] OR "Campylobacter"[Mesh] OR “Campylobacter infections“ [Mesh] | 3655 |
| #9 #1 AND #8 | 11 |
| #10 chikungunya[tw] OR chikv[tw] OR “chikungunya virus”[Mesh] OR “Chikungunya fever“ [Mesh] | 3345 |
| #11 #1 AND #10 | 43 |
| #12 Chlamydia[tw] OR c.trachomatis[tw] OR c.pneumoniae[tw] OR c.psittaci[tw] OR trachoma[tw] OR inclusion conjunctivitis[tw] OR ophthalmia neonatorum[tw] OR Lymphogranuloma venereum[tw] OR LGV[tw] OR Psittacosis[tw] OR “Chlamydia infections“ [Mesh] OR “Chlamydia trachomatis“ [Mesh] | 5688 |
| #13 #1 AND #12 | 124 |
| #14 cholera[tw] OR Vibrio cholerae[tw] OR v. cholerae[tw] OR cholera[Mesh] OR “Vibrio cholerae”[Mesh] | 4433 |
| #15 #1 AND #14 | 34 |
| #16 cryptosporidi*[tw] OR c.parvum[tw] OR “cryptosporidiosis“ [Mesh] OR “cryptosporidium“ [Mesh] | 2156 |
| #17 #1 AND #16 | 18 |
| #18 dengue[tw] OR DENV[tw] OR dengue[Mesh] OR dengue virus[Mesh] | 10098 |
| #19 #1 AND #18 | 189 |
| #20 diphtheria[tw] OR Corynebacterium diphtheriae[tw] OR Klebs-Loffler bacillus[tw]^[[1]](#footnote-1)^ OR diphtheria[Mesh] OR Corynebacterium diphtheriae[Mesh] OR Corynebacterium infections[Mesh] | 3151 |
| #21 #1 AND #20 | 1 |
| #22 echinococc*[tw] OR E. granulosus[tw] OR E. multilocularis[tw] OR hydatid cyst[tw] OR Hydatid disease*[tw] OR Echinococcus[Mesh] OR Echinococcosis[Mesh] | 3341 |
| #23 #1 AND #22 | 15 |
| #24 giardia*[tw] OR Lamblia[tw] OR Lamblias[tw] OR Lambliasis[tw] OR G. intestinalis[tw] OR “G. duodenalis”[tw] OR “G. muris”[tw] OR Giardia[Mesh] OR Giardiasis[Mesh] | 2151 |
| #25 #1 AND #24 | 18 |
| #26 gonorrhoea*[tw] OR gonorrhea*[tw] OR gonococc*[tw] OR N. gonorrhoeae[tw] OR gonorrhea[Mesh] OR Neisseria gonorrhoeae[Mesh] | 3516 |
| #27 #1 AND #26 | 107 |
| #28 hepatitis A[tw] OR hep A[tw] OR hepatitis virus a[tw] OR hav[tw] OR “hepatitis A”[Mesh] OR "Hepatitis A virus"[Mesh] | 3137 |
| #29 #1 AND #28 | 11 |
| #30 hepatitis B[tw] OR hep b[tw] OR hepatitis virus b[tw] OR HBV[tw] OR dane particle[tw] OR “hepatitis B”[Mesh] OR "Hepatitis B virus"[Mesh] | 22913 |
| #31 #1 AND #30 | 198 |
| #32 hepatitis C[tw] OR “hep C”[tw] OR hepatitis virus c[tw] OR HCV[tw] OR hepacivirus c[tw] OR “hepatitis C”[Mesh] OR hepacivirus[Mesh] | 28073 |
| #33 #1 AND #32 | 226 |
| #34 HIV[tw] OR human immunodeficiency virus*[tw] OR human immune deficiency virus*[tw] OR "immune deficiency associated virus"[tw] OR "immune deficiency associated viruses"[tw] OR " immunodeficiency associated virus"[tw] OR " immunodeficiency associated viruses"[tw] OR acquired immunodeficiency syndrome[tw] OR acquired immune deficiency syndrome[tw] OR AIDS[tw] OR HIV[Mesh] OR Acquired Immunodeficiency Syndrome[Mesh] | 101957 |
| #35 #1 AND #34 | 1173 |
| #36 Haemophilus influenzae type b[tw] OR Hemophilus influenzae type b[tw] OR Hib[tw] OR H. influenzae type b[tw] OR Haemophilus influenzae group B[tw] OR Hemophilus influenzae group B[tw] OR Haemophilus influenzae type b[Mesh] | 864 |
| #37 #1 AND #36 | 4 |
| #38 flu[tw] OR influenza*[tw] OR H1N1[tw] OR Influenza, Human[Mesh] OR influenza in birds[Mesh] OR "Influenza A Virus, H1N1 Subtype"[Mesh] | 32065 |
| #39 #1 AND #38 | 343 |
| #40 Legionella[tw] OR L. pneumophila[tw] OR Legionnaire’s disease[tw] OR legionnaires’ disease[tw] OR Pontiac fever[tw] OR Legionella pneumophila[Mesh] OR Legionnaires' Disease[Mesh] | 2009 |
| #41 #1 AND #40 | 19 |
| #42 leptospirosis[tw] OR Leptospira[tw] OR L. interrogans[tw] OR L. kirschneri[tw] OR L. borgpetersenii[tw] OR L. santarosai[tw] OR L. noguchii[tw] OR L. weilii[tw] OR L. alexanderi[tw] OR L. alstoni[tw] OR L. kmetyi[tw] OR Leptospira[Mesh] OR Leptospirosis[Mesh] | 2134 |
| #43 #1 AND #42 | 49 |
| #44 Listerios*[tw] OR Listeria*[tw] OR L. monocytogenes[tw] OR L. seeligeri[tw] OR L. ivanovii[tw] OR L. welshimeri[tw] OR L. grayi[tw] OR L. innocua[tw] OR L. marthii[tw] OR L. rocourtiae[tw] OR Listeria[Mesh] OR Listeriosis[Mesh] | 5589 |
| #45 #1 AND #44 | 9 |
| #46 Lyme disease[tw] OR Borrelia[tw] OR neuroborreliosis[tw] OR Lyme disease[Mesh] OR Borrelia burgdorferi[Mesh] | 3767 |
| #47 #1 AND #46 | 16 |
| #48 malaria[tw] OR Plasmodium[tw] OR P. falciparum[tw] OR P. vivax[tw] OR P. ovale[tw] OR P. malariae[tw] OR P. knowlesi[tw] OR Paludism[tw] OR Malaria[Mesh] OR Plasmodium[Mesh] | 24633 |
| #49 #1 AND #48 | 1219 |
| #50 Measles[tw] OR Rubeola[tw] OR Measles[Mesh] OR Measles virus[Mesh] OR morbillivirus[MeSH] | 4918 |
| #51 #1 AND #50 | 8 |
| #52 mumps[tw] OR parotitis[tw] OR "epidemic parotid virus*"[tw] OR "epidemic parotiditis virus*"[tw] OR "epidemic parotitides virus*"[tw] OR "epidemic parotitus virus*"[tw] OR mumps[Mesh] OR mumps virus[Mesh]^[[2]](#footnote-2)^ | 1871 |
| #53 #1 AND #52 | 3 |
| #54 Neisseria meningitidis[tw] OR meningococc*[tw] OR meningitis, bacterial[Mesh] OR meningitis, meningococcal[Mesh] OR Neisseria meningitidis[Mesh] | 5140 |
| #55 #1 AND #54 | 33 |
| #56 pertussis[tw] OR whooping cough[tw] OR Whooping Cough[Mesh] OR Bordetella pertussis[Mesh] OR Pertussis Toxin[Mesh] | 3950 |
| #57 #1 AND #56 | 11 |
| #58 plague*[tw] OR Yersinia pestis[tw] OR y. pestis[tw] OR Black death[tw] OR plague[Mesh] OR Yersinia[Mesh] OR Yersinia infections[Mesh] OR "Yersinia pestis"[Mesh] | 3862 |
| #59 #1 AND #58 | 13 |
| #60 polio[tw] OR poliomyelitis[tw] OR poliovirus*[tw] OR polioenterovirus*[tw] OR poliomyelitis[Mesh] OR poliovirus[Mesh] | 3464 |
| #61 #1 AND #60 | 2 |
| #62 Q fever[tw] OR Coxiella[tw] OR C. burnetii[tw] OR Abattoir fever[tw] OR Coxiellosis[tw] OR Q fever[Mesh] OR coxiella[Mesh] | 1412 |
| #63 #1 AND #62 | 4 |
| #64 rabies[tw] OR rabies[Mesh] OR rabies virus[Mesh] | 2738 |
| #65 #1 AND #64 | 6 |
| #66  rubella[tw] OR rubellavirus[tw] OR RuV[tw] OR German measles[tw] OR rubella[Mesh] OR rubella virus[Mesh] | 2264 |
| #67 #1 AND #66 | 9 |
| #68 salmonellosis[tw] OR Salmonella enterica[tw] OR Salmonella*[tw] OR S. Enteritidis[tw] OR S.Typhimurium[tw] OR S.Typhi[tw] OR Salmonella enterica[Mesh] OR Salmonella infections[Mesh] OR "Salmonella"[Mesh] | 15416 |
| #69 #1 AND #68 | 93 |
| #70 SARS*[tw] OR severe acute respiratory syndrome[tw] OR severe acute respiratory syndrome-related coronavirus[tw] OR Severe Acute Respiratory Syndrome[Mesh] OR SARS virus[Mesh] | 2043 |
| #71 #1 AND #70 | 4 |
| #72 verocytotoxin-producing Escherichia coli[tw] OR Shiga toxin-producing Escherichia coli[tw] OR VTEC[tw] OR STEC[tw] OR verocytotoxin-producing E. coli[tw] OR Shiga toxin-producing E. coli[tw] OR STEC/VTEC[tw] OR EHEC[tw] OR SLTEC[tw] OR Shiga-Toxigenic Escherichia coli[Mesh] OR non-O157[tw] OR coli-O157[tw] | 4184 |
| #73 #1 AND #72 | 36 |
| #74 Shigella*[tw] OR shigellosis[tw] OR S. dysenteriae[tw] OR S. flexneri[tw] OR S. boydii[tw] OR S. sonnei[tw] OR bacillary dysentery[tw] OR Shigella[Mesh] OR Dysentery, Bacillary[Mesh] | 2637 |
| #75 #1 AND #74 | 10 |
| #76 smallpox*[tw] OR Variola*[tw] OR smallpox[Mesh] OR variola virus[Mesh] | 820 |
| #77 #1 AND #76 | 1 |
| #78  Streptococcus pneumonia*[tw] OR S. pneumonia*[tw] OR diplococcus pneumonia*[tw] OR d. pneumonia*[tw] OR pneumococcus[tw] OR pneumococcal[tw] OR IPD[tw] OR Streptococcus pneumoniae[Mesh] OR Pneumococcal Infections[Mesh] | 10613 |
| #79 #1 AND #78 | 36 |
| #80 Syphilis[tw] OR Treponema pallidum[tw] OR T. pallidum[tw] OR Chancre[tw] OR syphilis[Mesh] OR Treponema pallidum[Mesh] | 4750 |
| #81 #1 AND #80 | 214 |
| #82 tetanus[tw] OR Clostridium tetani[tw] OR C. tetani[tw] OR Bacillus tetani[tw] OR B. tetani[tw] OR tetanus[Mesh] OR Clostridium tetani[Mesh] | 3296 |
| #83 #1 AND #82 | 10 |
| #84 tick borne encephalitis*[tw] OR tick-borne encephalitis*[tw] OR TBE*[tw] OR Encephalitis, Tick-Borne [Mesh] OR Encephalitis Viruses, Tick-Borne[Mesh] | 2070 |
| #85 #1 AND #84 | 1 |
| #86 toxoplasm*[tw] OR Toxoplasma[Mesh] OR Toxoplasmosis, Congenital[Mesh] | 5575 |
| #87 #1 AND #86 | 24 |
| #88 Transmissible spongiform encephalopath*[tw] OR TSEs[tw] OR Bovine spongiform encephalopath*[tw] OR Mad cow disease[tw] OR Encephalopathy, Bovine Spongiform[Mesh] | 717 |
| #89 #1 AND #88 | 6 |
| #90 trichina*[tw] OR trichinellosis[tw] OR trichinosis[tw] OR Trichinella*[tw] OR T. britovi[tw] OR T. murrelli[tw] OR T. nativa[tw] OR T. nelson[tw] OR T. spiralis[tw] OR T. papuae[tw] OR T. pseudospiralis[tw] OR T. zimbabwensis[tw] OR Trichinellosis[Mesh] OR Trichinella[Mesh] | 628 |
| #91 #1 AND #90 | 2 |
| #92 tuberculosis[tw] OR MTB[tw] OR LTBI[tw] OR koch’s disease[tw] OR M. africanum[tw] OR M. canetti[tw] OR M. caprae[tw] OR M. orygis[tw] OR tuberculosis[Mesh] OR Mycobacterium tuberculosis[Mesh] | 42320 |
| #93 #1 AND #92 | 365 |
| #94 typhoid[tw] OR paratyphoid[tw] OR Salmonella Typhi*[tw] OR Salmonella Paratyphi*[tw] OR Salmonella Paratyphosa[tw] OR Enteric fever[tw] OR salmonella schottmuelleri[tw] OR salmonella hirschfeldii[tw] OR S. Typhi[tw] OR S. paratyphi[tw] OR S. schottmuelleri[tw] OR S. hirschfeldii[tw] OR Typhoid fever[Mesh] OR paratyphoid fever[Mesh] | 6933 |
| #95 #1 AND #94 | 63 |
| #96 tularemia[tw] OR tularaemia[tw] OR Francisella tularensis[tw] OR Pasteurella tularensis[tw] OR bacterium tularense[tw] OR tularemia[Mesh] OR Francisella tularensis[Mesh] | 976 |
| #97 #1 AND #96 | 7 |
| #98 ((prion[tw] OR prions[tw]) AND (variant[tw] AND (Creutzfeldt-Jakob[tw] OR Creutzfeldt Jakob[tw] OR CJD[tw] OR Creutzfeldt-Jakob Syndrome[MeSH])) OR (variant cjd[tw] OR v cjd[tw] OR v-cjd[tw] OR vcjd[tw])) | 243 |
| #99 #1 AND #98 | 1 |
| #100 viral hemorrhagic fever*[tw] OR viral haemorrhagic fever*[tw] OR arenavirus[tw] OR filovirus[tw] OR ebola*[tw] OR EBOV[tw] OR Lassa[tw] OR Marburg virus[tw] OR Marburgvirus[tw] OR lassavirus[tw] OR marv[tw] OR Hantavirus[tw] OR Hanta virus[tw] OR Junin virus[tw] OR “Machupo mammarenavirus”[tw] OR nairovirus[tw] OR Crimean-congo hemorrhagic fever virus[tw] OR CCHF*[tw] OR zaire ebolavirus[tw] OR omsk hemorrhagic fever virus[tw] OR OHFV[tw] OR kyasanur forest disease virus[tw] OR rift valley fever virus[tw] OR RVF[tw] OR Hemorrhagic Fevers, Viral[Mesh] OR filoviridae[Mesh] OR Hemorrhagic Fever, Ebola[Mesh] OR ebolavirus[Mesh] OR Marburg Virus Disease[Mesh] OR Lassa virus[Mesh] OR Lassa fever[Mesh] | 14924 |
| #101 #1 AND #100 | 247 |
| #102 West Nile Virus[tw] OR West Nile flavivirus[tw] OR WNV[tw] OR West Nile Fever[tw] OR Egypt 101[tw] virus[tw] OR Kunjin virus[tw] OR West Nile Virus[Mesh] OR "West Nile Fever"[Mesh] | 2186 |
| #103 #1 AND #102 | 6 |
| #104 Yellow fever[tw] OR YFV[tw] OR Yellow fever[Mesh] OR Yellow fever virus[Mesh] | 1774 |
| #105 #1 AND #104 | 12 |
| #106 Yersiniosis[tw] OR Yersinia enterocolitica[tw] OR Y. enterocolitica[tw] OR Yersinia pseudotuberculosis[tw] OR Y. pseudotuberculosis[tw] OR Yersinia[Mesh] OR Yersinia infections[Mesh] | 1879 |
| #107 #1 AND #106 | 7 |
| #108 Zika[tw] OR Zikas[tw] OR Zikv[tw] OR zikav[tw] OR congenial zika[tw] OR Zika virus[Mesh] OR Zika virus infection[Mesh] | 6871 |
| #109 #1 AND #108 | 55 |
| #110 #3 OR #5 OR #7 OR #9 OR #11 OR #13 OR #15 OR #17 OR #19 OR #21 OR #23 OR #25 OR #27 OR #29 OR #31 OR #33 OR #35 OR #37 OR #39 OR #41 OR #43 OR #45 OR #47 OR #49 OR #51 OR #53 OR #55 OR #57 OR #59 OR #61 OR #63 OR #65 OR #67 OR #69 OR #71 OR #73 OR #75 OR #77 OR #79 OR #81 OR #83 OR #85 OR #87 OR #91 OR #93 OR #95 OR #97 OR #99 OR #101 OR #103 OR #105 OR #107 OR #109 | 3582 |

### AMR pathogen searches (PubMed)

**Date Limitation:** 2014-present

This search was run on 15 November 2019.

| Search strings | No of hits |
| --- | --- |
| #1  Point of care test*[tw] OR POCT[tw] OR rapid diagnostic test*[tw] OR rapid test*[tw] OR bedside test*[tw] OR near patient test*[tw] OR handheld device*[tw] OR handheld instrument*[tw] OR portable test*[tw] OR portable device*[tw] OR portable instrument*[tw] OR bedside computing[tw] OR Infectious disease testing[tw] OR infectious disease screening*[tw] OR point of care technolog*[tw] OR bedside technolog*[tw] OR point of care system*[tw] OR point-of-care systems[MeSH] OR point-of-care testing[MeSH] | 12828 |
| #2 Staphylococcus aureus[tw] OR micrococcus aureus[tw] OR microccus pyogenes[tw] OR Staphylococcus aureus[Mesh] OR Enterococcus faecium[tw] OR streptococcus faecium[tw] OR Enterococcus faecium[Mesh] OR Enterococcus faecalis[tw] OR streptococcus Group D[tw] OR streptococcus faecalis[tw] OR enterococcus fecalis[tw] OR enterococcus l form[tw] OR enterococcus proteiformis[tw] OR micrococcus ovalis[tw] OR micrococcus zymogenes[tw] OR paraghurt[tw] OR streptococcus fecalis[tw] OR streptococcus glycerinaceus[tw] OR streptococcus liquefaciens[tw] OR streptococcus ovalis[tw] OR th 69[tw] OR Enterococcus faecalis[MeSH] OR Escherichia coli[tw] OR e coli[tw] OR e. coli[tw] OR Alkalescens-Dispar Group[tw] OR EAggEC[tw] OR Bacillus coli[tw] OR "Bacillus escherichii"[tw] OR Bacterium coli[tw] OR "bacterium E3"[tw] OR coli bacillus[tw] OR coli bacterium[tw] OR colibacillus[tw] OR colon bacillus[tw] OR Enterococcus coli[tw] OR "Escherichia alkalescens dispart"[tw] OR Escherichia coli[Mesh] OR Klebsiella pneumoniae[tw] OR Klebsiella rhinoscleromatis[tw] OR "b. Friedlander"[tw] OR bacillus pneumoniae[tw] OR Bacterium pneumoniae crouposae[tw] OR bacterium pneumonie crouposae[tw] OR "friedlaender bacillus"[tw] OR friedlander bacillus[tw] OR "hyalococcus pneumoniae"[tw] OR k. Pneumoniae[tw] OR klebsiella crouposa[tw] OR Klebsiella Pn[tw] OR klebsiella pneumonia[tw] OR Klebsiella pneumoniae aerogenes[tw] OR pneumobacillus[tw] OR Klebsiella pneumoniae[Mesh] OR Acinetobacter baumannii[tw] OR Acinetobacter baumannii[MeSH] OR "Acinetobacter Infections"[Mesh] OR Pseudomonas aeruginosa[tw] OR Pseudomonas pyocyanea[tw] OR Bacillus aeruginosus[tw] OR Bacillus pyocyaneus[tw] OR "Bacterium aeruginosum" [tw] OR Bacterium pyocyaneum[tw] OR "blue pus organism" [tw] OR "Micrococcus pyocyaneus" [tw] OR P. Aeruginosa[tw] OR "Pseudomonas polycolor" [tw] OR "Pseudomonas pyoceaneus" [tw] OR Pseudomonas pyocyaneus[tw] OR Pseudomonas aeruginosa[MeSH] OR staphylococcus argenteus[tw] | 119401 |
| #3 antimicrobial resistance[tw] OR antimicrobial testing[tw] OR Antimicrobial susceptibility[tw] OR Antibiotic susceptibility*[tw] OR antibiotic resistance[tw] OR antibiotic testing[tw] OR multi-drug resistant[tw] OR multidrug-resistant[tw] OR carbapenem-resistant[tw] OR carbapenemase[tw] OR methicillin resistant[tw] OR vancomycin-resistant[tw] OR extended-spectrum beta-lactamase[tw] OR ESBL[tw] OR AMR[tw] OR resistan*[tw] OR MDR[tw] OR XDR[tw] OR PDR[tw] OR pandrug resistance[tw] OR pan-drug resistance[tw] OR Drug Resistance, Microbial[Mesh] OR "Disease Resistance"[Mesh] OR "Drug Resistance"[Mesh:NoExp] OR "Drug Resistance, Bacterial"[Mesh] | 332622 |
| #4  #1 AND #2 AND #3 | 121 |

### Nosocomial pathogen searches (PubMed)

**Date Limitation:** 2014-present

This search was run on 15 November 2019.

| Search strings | No of hits |
| --- | --- |
| #1  Point of care test*[tw] OR POCT[tw] OR rapid diagnostic test*[tw] OR rapid test*[tw] OR bedside test*[tw] OR near patient test*[tw] OR handheld device*[tw] OR handheld instrument*[tw] OR portable test*[tw] OR portable device*[tw] OR portable instrument*[tw] OR bedside computing[tw] OR Infectious disease testing[tw] OR infectious disease screening*[tw] OR point of care technolog*[tw] OR bedside technolog*[tw] OR point of care system*[tw] OR point-of-care systems[MeSH] OR point-of-care testing[MeSH] | 12828 |
| #2 Streptococcus pneumoniae[tw] OR Pneumococc*[tw] OR Diplococcus pneumoniae[tw] OR Micrococcus pneumoniae[tw] OR Streptococcus pneumoniae[Mesh] OR Pneumococcal Infections[Mesh] OR Staphylococcus aureus[tw] OR micrococcus aureus[tw] OR microccus pyogenes[tw] OR Staphylococcus aureus[Mesh] OR Enterococcus faecium[tw] OR streptococcus faecium[tw] OR Enterococcus faecium[Mesh] OR Enterococcus faecalis[tw] OR streptococcus Group D[tw] OR streptococcus faecalis[tw] OR enterococcus fecalis[tw] OR enterococcus l form[tw] OR enterococcus proteiformis[tw] OR micrococcus ovalis[tw] OR micrococcus zymogenes[tw] OR paraghurt[tw] OR streptococcus fecalis[tw] OR streptococcus glycerinaceus[tw] OR streptococcus liquefaciens[tw] OR streptococcus ovalis[tw] OR th 69[tw] OR Enterococcus faecalis[MeSH] OR Escherichia coli[tw] OR e coli[tw] OR e. coli[tw] OR Alkalescens-Dispar Group[tw] OR EAggEC[tw] OR Bacillus coli[tw] OR "Bacillus escherichii"[tw] OR Bacterium coli[tw] OR "bacterium E3"[tw] OR coli bacillus[tw] OR coli bacterium[tw] OR colibacillus[tw] OR colon bacillus[tw] OR Enterococcus coli[tw] OR "Escherichia alkalescens dispart"[tw] OR Escherichia coli[Mesh] OR Klebsiella pneumoniae[tw] OR Klebsiella rhinoscleromatis[tw] OR "b. Friedlander"[tw] OR bacillus pneumoniae[tw] OR Bacterium pneumoniae crouposae[tw] OR bacterium pneumonie crouposae[tw] OR "friedlaender bacillus"[tw] OR friedlander bacillus[tw] OR "hyalococcus pneumoniae"[tw] OR k. Pneumoniae[tw] OR klebsiella crouposa[tw] OR Klebsiella Pn[tw] OR klebsiella pneumonia[tw] OR Klebsiella pneumoniae aerogenes[tw] OR pneumobacillus[tw] OR Klebsiella pneumoniae[Mesh] OR Acinetobacter baumannii[tw] OR Acinetobacter baumannii[MeSH] OR "Acinetobacter Infections"[Mesh] OR Pseudomonas aeruginosa[tw] OR Pseudomonas pyocyanea[tw] OR Bacillus aeruginosus[tw] OR Bacillus pyocyaneus[tw] OR "Bacterium aeruginosum" [tw] OR Bacterium pyocyaneum[tw] OR "blue pus organism" [tw] OR "Micrococcus pyocyaneus" [tw] OR P. Aeruginosa[tw] OR "Pseudomonas polycolor" [tw] OR "Pseudomonas pyoceaneus" [tw] OR Pseudomonas pyocyaneus[tw] OR Pseudomonas aeruginosa[MeSH] OR Clostridium difficile[tw] OR Clostridioides difficile[tw] or c diff[tw] OR c. diff[tw] OR c difficile[tw] OR c. difficile[tw] OR Pseudomembranous colitis[tw] OR Clostridium difficile[MeSH] OR "Clostridium sordellii"[Mesh] OR "Clostridium perfringens"[Mesh] OR Candida[tw] OR C. glabrata[tw] OR candida glabrata[tw] OR candida auris[tw] OR Candidiasis[tw] OR Candidosis[tw] OR candidas[tw] OR monilia[tw] OR monilias[tw] OR torulopsis utilis[tw] OR Candida[MeSH] OR Candidiasis[MeSH] OR staphylococcus argenteus[tw] | 149370 |
| #3 Nosocomial infection*[tw] OR Healthcare associated infection*[tw] OR healthcare acquired infection*[tw] OR Health care associated infection*[tw] OR health care acquired infection*[tw] OR HCAI[tw] OR Hospital acquired infection*[tw] OR HAI[tw] OR cross infection*[tw] OR hospital infection*[tw] OR "Cross Infection"[Mesh] | 17448 |
| #4  #1 AND #2 AND #3 | 33 |

### General infectious disease search (PubMed)

**Date Limitation:** 2014-present

This search was run on 15 November 2019.

| Search strings | No of hits |
| --- | --- |
| #1 Point of care test*[tw] OR POCT[tw] OR rapid diagnostic test*[tw] OR rapid test*[tw] OR bedside test*[tw] OR near patient test*[tw] OR handheld device*[tw] OR handheld instrument*[tw] OR portable test*[tw] OR portable device*[tw] OR portable instrument*[tw] OR bedside computing[tw] OR Infectious disease testing[tw] OR infectious disease screening*[tw] OR point of care technolog*[tw] OR bedside technolog*[tw] OR point of care system*[tw] OR point-of-care systems[MeSH] OR point-of-care testing[MeSH] | 12828 |
| #2 antimicrobial resistance[tw] OR antimicrobial testing[tw] OR Antimicrobial susceptibility[tw] OR Antibiotic susceptibility*[tw] OR antibiotic resistance[tw] OR antibiotic testing[tw] OR multi-drug resistant[tw] OR multidrug-resistant[tw] OR carbapenem-resistant[tw] OR carbapenemase[tw] OR methicillin resistant[tw] OR vancomycin-resistant[tw] OR extended-spectrum beta-lactamase[tw] OR ESBL[tw] OR AMR[tw] OR resistan*[tw] OR MDR[tw] OR XDR[tw] OR PDR[tw] OR pandrug resistance[tw] OR pan-drug resistance[tw] OR Drug Resistance, Microbial[Mesh] OR "Disease Resistance"[Mesh] OR "Drug Resistance"[Mesh:NoExp] OR "Drug Resistance, Bacterial"[Mesh] OR Nosocomial infection*[tw] OR Healthcare associated infection*[tw] OR healthcare acquired infection*[tw] OR Health care associated infection*[tw] OR health care acquired infection*[tw] OR HCAI[tw] OR Hospital acquired infection*[tw] OR HAI[tw] OR cross infection*[tw] OR hospital infection*[tw] OR "Cross Infection"[Mesh] OR Infectious disease*[tw] OR Communicable disease*[tw] OR infection*[tw] OR Communicable Diseases[Mesh] OR Sexually transmitted infection*[tw] OR Sexually transmitted disease*[tw] OR STI[tw] OR STD[tw] OR Venereal disease*[tw] OR Sexually Transmitted Diseases[Mesh] OR Respiratory infection*[tw] OR Respiratory tract infection*[tw] OR Pleural Empyema*[tw] OR Thoracic Empyemas[tw] OR Pyothorax[tw] OR Tuberculous Empyema[tw] OR "Respiratory Tract Infections"[Mesh] OR Gastrointestinal infection*[tw] OR vaccine-preventable infection*[tw] OR vaccine-preventable disease*[tw] OR VPD[tw] OR VPI[tw] OR "Vaccines"[Mesh] OR Emerging infection*[tw] OR Emerging infectious disease*[tw] OR Emerging communicable disease*[tw] OR re-emerging communicable disease*[tw] OR reemerging communicable disease*[tw] OR reemerging infectious disease*[tw] OR re-emerging infectious disease*[tw] OR re-emerging infection*[tw] OR reemerging infection*[tw] OR "Communicable Diseases, Emerging"[Mesh] OR Sepsis[tw] OR Septicemia[tw] OR Septicaemia[tw] OR Septic shock[tw] OR Bloodstream infection*[tw] OR Blood infection*[tw] OR Pyemia[tw] OR Pyemias[tw] OR Pyohemia[tw] OR Pyohemias[tw] OR Pyaemia[tw] OR Pyaemias[tw] OR Blood Poisoning[tw] OR Sepsis[MeSH] OR Viral meningitis[tw] OR Bacterial meningitis[tw] OR Fungal meningitis[tw] OR Parasitic meningitis[tw] OR Meningitis, Viral[Mesh] OR Meningitis, Bacterial[Mesh] OR Meningitis, Fungal[Mesh] OR urinary tract infection*[tw] OR UTI[tw] OR bacteriuria[tw] OR pyuria[tw] OR urinary tract infections[Mesh] OR Bacterial pneumonia*[tw] OR viral pneumonia*[tw] OR fungal pneumonia*[tw] OR Infectious pneumonia*[tw] OR Pneumonia, Bacterial[Mesh] OR Pneumonia, Viral [Mesh] OR diarrhoea[tw] OR diarrhea[tw] OR diarrheal disease*[tw] OR diarrhoeal disease*[tw] OR "Diarrhea"[Mesh] | 845440 |
| #3 #1 AND #2 | 4352 |

### PubMed search summary

| Search summary | Total number of results (before de-duplication) | Number of unique results (after de-duplication)^[[3]](#footnote-3)^ |
| --- | --- | --- |
| 56 Diseases | 3852 | 3852 |
| AMR pathogens | 121 | 108 |
| Nosocomial pathogens | 33 | 13 |
| General infectious diseases | 4352 | 1642 |
| **Total** | **8358** | **5345** |

## Embase search strings and number of search hits

### 56 diseases

**Date Limitation:** 2014-2019

This search was run on 18 November 2019.

| Search strings | No of hits |
| --- | --- |
| #1 'Point of care test*':ab,ti,kw OR POCT:ab,ti,kw OR 'rapid diagnostic test*':ab,ti,kw OR 'rapid test*':ab,ti,kw OR 'bedside test*':ab,ti,kw OR 'near patient test*':ab,ti,kw OR 'handheld device*':ab,ti,kw OR 'handheld instrument*':ab,ti,kw OR 'portable test*':ab,ti,kw OR 'portable device*':ab,ti,kw OR 'portable instrument*':ab,ti,kw OR 'bedside computing':ab,ti,kw OR 'Infectious disease testing':ab,ti,kw OR 'infectious disease screening*':ab,ti,kw OR 'point of care technolog*':ab,ti,kw OR 'bedside technolog*':ab,ti,kw OR 'point of care system*':ab,ti,kw OR 'point of care system'/exp OR 'point of care testing'/exp AND [2014-2019]/py | 19039 |
| #2 Anthrax:ab,ti,kw OR anthracis:ab,ti,kw OR 'Anthrax'/exp OR 'Bacillus anthracis'/exp AND [2014-2019]/py | 2524 |
| #3 #1 AND #2 | 15 |
| #4 botulism:ab,ti,kw OR botulinum:ab,ti,kw OR botulism/exp OR 'Clostridium botulinum'/exp AND [2014-2019]/py | 9073 |
| #5 #1 AND #4 | 17 |
| #6 brucellosis:ab,ti,kw OR Brucella:ab,ti,kw OR 'b. abortus':ab,ti,kw OR 'b.canis':ab,ti,kw OR 'b. melitensis':ab,ti,kw OR 'b.ovis':ab,ti,kw OR 'b. suis':ab,ti,kw OR brucellosis/exp OR Brucella/exp AND [2014-2019]/py | 4238 |
| #7 #1 AND #6 | 33 |
| #8 campylobacteriosis:ab,ti,kw OR campylobacter*:ab,ti,kw OR 'c.coli':ab,ti,kw OR 'c.fetus':ab,ti,kw OR 'c.hyointestinalis':ab,ti,kw OR 'c.jejuni':ab,ti,kw OR 'c.lari':ab,ti,kw OR 'c.rectus':ab,ti,kw OR 'c.sputorum':ab,ti,kw OR ‘c.upsaliensis':ab,ti,kw OR campylobacter/exp OR 'campylobacteriosis'/exp AND [2014-2019]/py | 5466 |
| #9  #1 AND #8 | 28 |
| #10 (chikungunya NEAR/2 (virus OR fever)):ab,ti,kw OR chikv:ab,ti,kw OR 'chikungunya virus'/exp OR chikungunya/exp AND [2014-2019]/py | 4442 |
| #11 #1 AND #10 | 65 |
| #12 Chlamydia:ab,ti,kw OR 'c.trachomatic':ab,ti,kw OR 'c.pneumoniae':ab,ti,kw OR ‘c.psittaci':ab,ti,kw OR trachoma:ab,ti,kw OR 'inclusion conjunctivitis':ab,ti,kw OR 'ophthalmia neonatorum':ab,ti,kw OR 'Lymphogranuloma venereum':ab,ti,kw OR LGV:ab,ti,kw OR psittacosis:ab,ti,kw OR Chlamydia/exp OR 'Chlamydia trachomatis'/exp AND [2014-2019]/py | 9657 |
| #13  #1 AND #12 | 233 |
| #14  cholera:ab,ti,kw OR 'vibrio cholerae':ab,ti,kw OR 'v. cholerae':ab,ti,kw OR cholera/exp OR 'vibrio cholerae'/exp AND [2014-2019]/py | 6288 |
| #15 #1 AND #14 | 51 |
| #16 cryptosporidi*:ab,ti,kw OR 'c.parvum':ab,ti,kw OR cryptosporidiosis/exp OR cryptosporidium/exp AND [2014-2019]/py | 3163 |
| #17 #1 AND #16 | 37 |
| #18 dengue:ab,ti,kw OR DENV:ab,ti,kw OR dengue/exp OR 'Dengue virus'/exp AND [2014-2019]/py | 14574 |
| #19 #1 AND #18 | 307 |
| #20 diphtheria:ab,ti,kw OR 'Corynebacterium diphtheriae':ab,ti,kw OR 'Klebs-Loffler bacillus':ab,ti,kw OR diphtheria/exp OR 'Corynebacterium diphtheriae'/exp OR 'Corynebacterium infection'/exp AND [2014-2019]/py | 4714 |
| #21  #1 AND #20 | 3 |
| #22 echinococc*:ab,ti,kw OR 'E. granulosus':ab,ti,kw OR 'E. multilocularis':ab,ti,kw OR 'Hydatid cyst':ab,ti,kw OR 'Hydatid disease*':ab,ti,kw OR 'Echinococcus'/exp OR 'echinococcosis'/exp AND [2014-2019]/py | 4946 |
| #23 #1 AND #22 | 24 |
| #24 giardia*:ab,ti,kw OR Lamblia:ab,ti,kw OR Lamblias:ab,ti,kw OR Lambliasis:ab,ti,kw OR ‘G. intestinalis':ab,ti,kw OR 'g. duodenalis':ab,ti,kw OR 'g. muris':ab,ti,kw OR giardia/exp OR giardiasis/exp AND [2014-2019]/py | 3451 |
| #25 #1 AND #24 | 38 |
| #26 gonorrhoea*:ab,ti,kw OR gonorrhea*:ab,ti,kw OR gonococc*:ab,ti,kw OR N. gonorrhoeae:ab,ti,kw gonorrhea/exp OR Neisseria gonorrhoeae/exp AND [2014-2019]/py | 3661 |
| #27 #1 AND #26 | 149 |
| #28 'hepatitis A':ab,ti,kw OR 'hep A':ab,ti,kw OR 'hepatitis virus A':ab,ti,kw OR hav:ab,ti,kw OR ‘hepatitis A’/exp OR 'Hepatitis A virus'/exp AND [2014-2019]/py | 4744 |
| #29 #1 AND #28 | 22 |
| #30 'hepatitis B':ab,ti,kw OR 'hep b':ab,ti,kw OR 'hepatitis virus B':ab,ti,kw OR HBV:ab,ti,kw OR 'dane particle':ab,ti,kw OR 'hepatitis B'/exp OR 'Hepatitis B virus'/exp AND [2014-2019]/py | 46939 |
| #31 #1 AND #30 | 380 |
| #32 'hepatitis C':ab,ti,kw OR 'hep C':ab,ti,kw OR 'hepatitis virus C':ab,ti,kw OR HCV:ab,ti,kw OR 'hepacvirus c':ab,ti,kw OR 'hepatitis C'/exp OR 'Hepacivirus'/exp AND [2014-2019]/py | 60212 |
| #33 #1 AND #32 | 530 |
| #34 HIV:ab,ti,kw OR 'human immunodeficiency virus':ab,ti,kw OR 'human immune deficiency virus*':ab,ti,kw OR 'immune deficiency associated virus':ab,ti,kw OR 'immune deficiency associated viruses':ab,ti,kw OR 'immunodeficiency associated virus':ab,ti,kw OR 'immunodeficiency associated viruses':ab,ti,kw OR 'acquired immunodeficiency syndrome':ab,ti,kw OR 'acquired immune deficiency syndrome':ab,ti,kw OR AIDS:ab,ti,kw OR 'Human immunodeficiency virus'/exp OR 'acquired immune deficiency syndrome'/exp AND [2014-2019]/py | 145076 |
| #35 #1 AND #34 | 1948 |
| #36 'haemophilus influenza type b':ab,ti,kw OR 'hemophilus influenza type b':ab,ti,kw OR Hib:ab,ti,kw OR 'H. influenzae type b':ab,ti,kw OR 'hemophilus influenza group b':ab,ti,kw OR 'haemophilus influenza group b':ab,ti,kw OR 'Haemophilus influenzae type b'/exp AND [2014-2019]/py | 1267 |
| #37 #1 AND #36 | 4 |
| #38 flu:ab,ti,kw OR influenza*:ab,ti,kw OR H1N1:ab,ti,kw OR 'influenza'/exp OR 'avian influenza'/exp OR 'Influenza A virus (H1N1)'/exp AND [2014-2019]/py | 46940 |
| #39 #1 AND #38 | 505 |
| #40 legionella:ab,ti,kw OR 'L. pneumophila':ab,ti,kw OR (Legionnaire* near/2 disease*):ab,ti,kw OR 'pontiac fever':ab,ti,kw OR 'legionella pneumophila'/exp OR 'legionnaire disease'/exp AND [2014-2019]/py | 3091 |
| #41 #1 AND #40 | 23 |
| #42 leptospirosis:ab,ti,kw OR Leptospira:ab,ti,kw OR 'L. interrogans':ab,ti,kw OR 'L. kirschneri':ab,ti,kw OR 'L. borgpetrsenii':ab,ti,kw OR 'L. santarosai':ab,ti,kw OR 'L. noguchii':ab,ti,kw OR 'L. weilii':ab,ti,kw OR 'L. alexanderi':ab,ti,kw OR 'L. alstoni':ab,ti,kw OR 'L. kmetyi':ab,ti,kw OR 'Leptospira'/exp OR 'Leptospirosis'/exp AND [2014-2019]/py | 3104 |
| #43 #1 AND #42 | 83 |
| #44 Listerios*:ab,ti,kw OR Listeria*:ab,ti,kw OR 'L. monocytogenes':ab,ti,kw OR 'L. seeligeri':ab,ti,kw OR 'L. ivanovii':ab,ti,kw OR 'L. welshimeri':ab,ti,kw OR 'L. grayi':ab,ti,kw OR 'L. innocua':ab,ti,kw OR 'L. marthii':ab,ti,kw OR 'L. rocourtiae':ab,ti,kw OR 'Listeria'/exp OR 'listeriosis'/exp AND [2014-2019]/py | 7950 |
| #45 #1 AND #44 | 21 |
| #46 (Lyme* near/2 disease):ab,ti,kw OR Borrelia:ab,ti,kw OR neurobirreliosis:ab,ti,kw OR 'Lyme disease'/exp OR 'Borrelia burgdorferi'/exp AND [2014-2019]/py | 5285 |
| #47 #1 AND #46 | 28 |
| #48 malaria:ab,ti,kw OR Plasmodium:ab,ti,kw OR 'P. falciparum':ab,ti,kw OR 'P. vivax':ab,ti,kw OR 'P. ovale':ab,ti,kw OR 'P. malariae':ab,ti,kw OR 'P. knowlesi':ab,ti,kw OR Paludism:ab,ti,kw OR Malaria/exp OR Plasmodium/exp AND [2014-2019]/py | 36369 |
| #49 #1 AND #48 | 2020 |
| #50 Measles:ab,ti,kw OR Rubeola:ab,ti,kw OR Measles/exp OR 'Measles virus'/exp OR 'Morbillivirus'/exp AND [2014-2019]/py | 7247 |
| #51 #1 AND #50 | 22 |
| #52 mumps:ab,ti,kw OR parotitis:ab,ti,kw OR 'epidemic parotid virus*':ab,ti,kw OR 'epidemic parotiditis virus*':ab,ti,kw OR 'epidemic parotitides virus*':ab,ti,kw OR 'epidemic parotitus virus*':ab,ti,kw OR mumps/exp OR mumps virus/exp AND [2014-2019]/py | 889 |
| #53 #1 AND #52 | 3 |
| #54 'Neisseria meningitidis':ab,ti,kw OR meningococcus:ab,ti,kw OR meningococc*:ab,ti,kw OR 'bacterial meningitis'/exp OR 'epidemic meningitis'/exp OR 'Neisseria meningitidis'/exp AND [2014-2019]/py | 7464 |
| #55 #1 AND #54 | 38 |
| #56 pertussis:ab,ti,kw OR 'whooping cough':ab,ti,kw OR 'Whooping Cough'/exp OR 'Bordetella pertussis'/exp OR 'pertussis toxin'/exp | 38262 |
| #57 #1 AND #56 | 19 |
| #58 plague*:ab,ti,kw OR 'Yersinia pestis':ab,ti,kw OR 'Y. pestis':ab,ti,kw OR 'Black death':ab,ti,kw OR plague/exp OR Yersinia/exp OR 'Yersinia infections'/exp OR 'Yersinia pestis'/exp AND [2014-2019]/py | 6007 |
| #59 #1 AND #58 | 30 |
| #60 polio:ab,ti,kw OR poliomyelitis:ab,ti,kw OR poliovirus*:ab,ti,kw OR polioenterovirus*:ab,ti,kw OR poliomyelitis/exp OR 'Poliomyelitis virus'/exp AND [2014-2019]/py | 4987 |
| #61 #1 AND #60 | 6 |
| #62 'Q fever':ab,ti,kw OR Coxiella:ab,ti,kw OR 'C. burnetii':ab,ti,kw OR 'Abattoir fever':ab,ti,kw OR Coxiellosis:ab,ti,kw OR 'Q fever'/exp OR coxiella/exp AND [2014-2019]/py | 1843 |
| #63 #1 AND #62 | 10 |
| #64 rabies:ab,ti,kw OR rabies/exp OR 'rabies virus'/exp AND [2014-2019]/py | 3523 |
| #65 #1 AND #64 | 8 |
| #66 rubella:ab,ti,kw OR rubellavirus:ab,ti,kw OR RuV:ab,ti,kw OR 'German measles':ab,ti,kw OR rubella/exp OR 'rubella virus'/exp AND [2014-2019]/py | 3373 |
| #67 #1 AND #66 | 11 |
| #68 salmonellosis:ab,ti,kw OR 'Salmonella enterica':ab,ti,kw OR Salmonella:ab,ti,kw OR 'S. Enteritidis':ab,ti,kw OR 'S.Typhimurium':ab,ti,kw OR 'S. Typhi':ab,ti,kw OR Salmonella enterica/exp OR 'salmonellosis'/exp OR 'Salmonella'/exp AND [2014-2019]/py | 21969 |
| #69 #1 AND #68 | 160 |
| #70 SARS*:ab,ti,kw OR 'severe acute respiratory syndrome':ab,ti,kw OR 'severe acute respiratory syndrome-related coronavirus':ab,ti,kw OR 'severe acute respiratory syndrome'/exp OR 'SARS coronavirus'/exp AND [2014-2019]/py | 3017 |
| #71 #1 AND #70 | 18 |
| #72 'verocytotoxin-producing Escherichia coli':ab,ti,kw OR 'Shiga toxin-producing Escherichia coli':ab,ti,kw OR VTEC:ab,ti,kw OR STEC:ab,ti,kw OR 'verocytotoxin producing E. coli':ab,ti,kw OR 'Shiga toxin producing E. coli':ab,ti,kw OR EHEC:ab,ti,kw OR SLTEC:ab,ti,kw OR non-O157:ab,ti,kw OR coli-O157:ab,ti,kw OR 'Shiga toxin producing Escherichia coli'/exp AND [2014-2019]/py | 4406 |
| #73 #1 AND #72 | 33 |
| #74 Shigella*:ab,ti,kw OR shigellosis:ab,ti,kw OR 'S. dysenteriae':ab,ti,kw OR 'S. flexneri':ab,ti,kw OR 'S. boydii':ab,ti,kw OR 'S. sonnei':ab,ti,kw OR 'bacillary dysentery':ab,ti,kw OR Shigella/exp OR 'shigellosis'/exp AND [2014-2019]/py | 4920 |
| #75 #1 AND #74 | 18 |
| #76 smallpox*:ab,ti,kw OR Variola*:ab,ti,kw OR smallpox/exp OR 'Smallpox virus'/exp AND [2014-2019]/py | 1187 |
| #77 #1 AND #76 | 5 |
| #78 'Streptococcus pneumonia*':ab,ti,kw OR 'S. pneumonia*':ab,ti,kw OR 'diplococcus pneumonia*':ab,ti,kw OR 'd. pneumonia*':ab,ti,kw OR pneumococcal:ab,ti,kw OR IPD:ab,ti,kw OR pneumococcus:ab,ti,kw OR 'Streptococcus pneumoniae'/exp OR 'pneumococcal infection'/exp AND [2014-2019]/py | 19779 |
| #79  #1 AND #78 | 101 |
| #80 Syphilis:ab,ti,kw OR 'Treponema pallidum':ab,ti,kw OR 'T. pallidum':ab,ti,kw OR Chancre:ab,ti,kw OR syphilis/exp OR 'treponema pallidum'/exp AND [2014-2019]/py | 9084 |
| #81 #1 AND #80 | 422 |
| #82 tetanus:ab,ti,kw OR 'Clostridium tetani':ab,ti,kw OR 'C. tetani':ab,ti,kw OR 'Bacillus tetani':ab,ti,kw OR 'B. tetani':ab,ti,kw OR tetanus/exp OR 'Clostridium tetani'/exp AND [2014-2019]/py | 4882 |
| #83 #1 AND #82 | 11 |
| #84 'tick borne encephalitis*':ab,ti,kw OR 'tick-borne encephalitis*':ab,ti,kw OR TBE*:ab,ti,kw OR 'tick borne encephalitis'/exp OR 'Tick borne encephalitis virus'/exp AND [2014-2019]/py | 2214 |
| #85 #1 AND #84 | 1 |
| #86 toxoplasm*:ab,ti,kw OR Toxoplasma/exp OR 'congenital toxoplasmosis'/exp AND [2014-2019]/py | 7565 |
| #87 #1 AND #86 | 37 |
| #88 'transmissible spongiform encephalopath*':ab,ti,kw OR TSEs:ab,ti,kw OR 'Bovine spongiform encephalopath*':ab,ti,kw OR 'Mad cow disease':ab,ti,kw OR 'bovine spongiform encephalopathy'/exp AND [2014-2019]/py | 1057 |
| #89 #1 AND #88 | 12 |
| #90 trichina*:ab,ti,kw OR trichinellosis:ab,ti,kw OR trichinosis:ab,ti,kw OR Trichinella*:ab,ti,kw OR 't. britovi':ab,ti,kw OR 't. murrelli':ab,ti,kw OR 't. nativa':ab,ti,kw OR 't. nelson':ab,ti,kw OR 't. spiralis':ab,ti,kw OR 't. papuae':ab,ti,kw OR 't. pseudospiralis':ab,ti,kw OR 't. zimbabwensis':ab,ti,kw OR Trichinellosis/exp OR Trichinella/exp AND [2014-2019]/py | 866 |
| #91 #1 AND #90 | 3 |
| #92 tuberculosis:ab,ti,kw OR MTB:ab,ti,kw OR LTBI:ab,ti,kw OR 'koch* disease':ab,ti,kw OR 'm. africanum':ab,ti,kw OR 'm. canetti':ab,ti,kw OR 'm. caprae':ab,ti,kw OR 'm. orygis':ab,ti,kw OR tuberculosis/exp OR 'Mycobacterium tuberculosis'/exp AND [2014-2019]/py | 67411 |
| #93 #1 AND #92 | 602 |
| #94 typhoid:ab,ti,kw OR paratyphoid:ab,ti,kw OR 'Salmonella Typhi':ab,ti,kw OR 'Salmonella Paratyphi':ab,ti,kw OR 'salmonella schottmuelleri':ab,ti,kw OR 'salmonella hirschfeldii':ab,ti,kw OR 'S. Typhi':ab,ti,kw OR 'S. Paratyphi':ab,ti,kw OR 's. schottmuelleri':ab,ti,kw OR 's. hirschfeldii':ab,ti,kw OR 'Enteric fever':ab,ti,kw OR 'Typhoid fever'/exp OR 'paratyphoid fever'/exp AND [2014-2019]/py | 4467 |
| #95 #1 AND #94 | 75 |
| #96 tularemia:ab,ti,kw OR tularaemia:ab,ti,kw OR 'Francisella tularensis':ab,ti,kw OR 'pasteurella tularensis':ab,ti,kw OR 'bacterium tularense':ab,ti,kw OR tularemia/exp OR 'Francisella tularensis'/exp | 6522 |
| #97 #1 AND #96 | 10 |
| #98 ((prion:ab,ti,kw OR prions:ab,ti,kw) AND ((variant:ab,ti,kw AND (Creutzfeldt-Jakob*:ab,ti,kw OR CJD:ab,ti,kw OR 'Creutzfeldt Jakob disease'/exp))) OR (vCJD:ab,ti,kw OR 'variant cjd':ab,ti,kw OR v-cjd:ab,ti,kw OR vcjd:ab,ti,kw)) AND [2014-2019]/py | 433 |
| #99 #1 AND #98 | 1 |
| #100 'viral hemorrhagic fever*':ab,ti,kw OR 'viral haemorrhagic fever*':ab,ti,kw OR arenavirus:ab,ti,kw OR filovirus:ab,ti,kw OR ebola*:ab,ti,kw OR EBOV:ab,ti,kw OR Lassa:ab,ti,kw OR 'Marburg virus':ab,ti,kw OR Marburgvirus:ab,ti,kw OR lassavirus:ab,ti,kw OR marv:ab,ti,kw OR hantavirus:ab,ti,kw OR 'hanta virus':ab,ti,kw OR 'junin virus':ab,ti,kw OR 'Machupo mammarenavirus':ab,ti,kw OR nairovirus:ab,ti,kw OR 'Crimean-congo hemorrhagic fever virus':ab,ti,kw OR CCHF*:ab,ti,kw OR 'zaire ebolavirus':ab,ti,kw OR 'omsk hemorrhagic fever virus':ab,ti,kw OR OHFV:ab,ti,kw OR 'kyasanur forest disease virus':ab,ti,kw OR 'rift valley fever virus':ab,ti,kw OR RVF:ab,ti,kw OR 'virus hemorrhagic fever'/exp OR filoviridae/exp OR 'Ebola hemorrhagic fever'/exp OR ebolavirus/exp OR 'Marburg hemorrhagic fever'/exp OR 'Lassa virus'/exp OR 'Lassa fever'/exp AND [2014-2019]/py | 12362 |
| #101 #1 AND #100 | 201 |
| #102 ('west nile' NEAR/2 (fever OR virus)):ab,ti,kw OR 'West Nile flavivirus':ab,ti,kw OR 'egypt 101 virus':ab,ti,kw OR 'kunjin virus':ab,ti,kw OR WNV:ab,ti,kw OR 'West Nile Virus'/exp OR 'West Nile fever'/exp AND [2014-2019]/py | 3533 |
| #103 #1 AND #102 | 22 |
| #104 'Yellow fever':ab,ti,kw OR YFV:ab,ti,kw OR 'Yellow fever'/exp OR 'Yellow fever virus'/exp AND [2014-2019]/py | 2679 |
| #105 #1 AND #104 | 20 |
| #106 Yersiniosis:ab,ti,kw OR 'Yersinia enterocolitica':ab,ti,kw OR 'Y. enterocolitica':ab,ti,kw OR 'Y. pseudotuberculosis':ab,ti,kw OR 'Yersinia pseudotuberculosis':ab,ti,kw OR Yersinia/exp OR 'Yersinia infection'/exp AND [2014-2019]/py | 3841 |
| #107 #1 AND #106 | 27 |
| #108 Zika:ab,ti,kw OR Zikas:ab,ti,kw OR Zikv:ab,ti,kw OR Zikav:ab,ti,kw OR 'congenial zika':ab,ti,kw OR 'Zika virus'/exp OR 'Zika fever'/exp AND [2014-2019]/py | 9053 |
| #109 #1 AND #108 | 102 |
| #110 #3 OR #5 OR #7 OR #9 OR #11 OR #13 OR #15 OR #17 OR #19 OR #21 OR #23 OR #25 OR #27 OR #29 OR #31 OR #33 OR #35 OR #37 OR #39 OR #41 OR #43 OR #45 OR #47 OR #49 OR #51 OR #53 OR #55 OR #57 OR #59 OR #61 OR #63 OR #65 OR #67 OR #69 OR #71 OR #73 OR #75 OR #77 OR #79 OR #81 OR #83 OR #85 OR #87 OR #91 OR #93 OR #95 OR #97 OR #99 OR #101 OR #103 OR #105 OR #107 OR #109 | 6150 |

### AMR pathogen searches (Embase)

**Date Limitation:** 2014-2019

This search was run on 18 November 2019.

| Search strings | No of hits |
| --- | --- |
| #1  'Point of care test*':ab,ti,kw OR POCT:ab,ti,kw OR 'rapid diagnostic test*':ab,ti,kw OR 'rapid test*':ab,ti,kw OR 'bedside test*':ab,ti,kw OR 'near patient test*':ab,ti,kw OR 'handheld device*':ab,ti,kw OR 'handheld instrument*':ab,ti,kw OR 'portable test*':ab,ti,kw OR 'portable device*':ab,ti,kw OR 'portable instrument*':ab,ti,kw OR 'bedside computing':ab,ti,kw OR 'Infectious disease testing':ab,ti,kw OR 'infectious disease screening*':ab,ti,kw OR 'point of care technolog*':ab,ti,kw OR 'bedside technolog*':ab,ti,kw OR 'point of care system*':ab,ti,kw OR 'point of care system'/exp OR 'point of care testing'/exp AND [2014-2019]/py | 19039 |
| #2 'Staphylococcus aureus':ab,ti,kw OR 'micrococcus aureus':ab,ti,kw OR 'microccus pyogenes':ab,ti,kw OR Staphylococcus aureus/exp OR 'Enterococcus faecium':ab,ti,kw OR 'streptococcus faecium':ab,ti,kw OR 'Enterococcus faecium'/exp OR 'Enterococcus faecalis':ab,ti,kw OR 'streptococcus Group D':ab,ti,kw OR 'streptococcus faecalis':ab,ti,kw OR 'enterococcus fecalis':ab,ti,kw OR 'enterococcus l form':ab,ti,kw OR 'enterococcus proteiformis':ab,ti,kw OR 'micrococcus ovalis':ab,ti,kw OR 'micrococcus zymogenes':ab,ti,kw OR paraghurt:ab,ti,kw OR 'streptococcus fecalis':ab,ti,kw OR 'streptococcus glycerinaceus':ab,ti,kw OR 'streptococcus liquefaciens':ab,ti,kw OR 'streptococcus ovalis':ab,ti,kw OR 'th 69':ab,ti,kw OR 'Enterococcus faecalis'/exp OR 'Escherichia coli':ab,ti,kw OR 'e coli':ab,ti,kw OR 'e. coli':ab,ti,kw OR 'Alkalescens-Dispar Group':ab,ti,kw OR EAggEC:ab,ti,kw OR 'Bacillus coli':ab,ti,kw OR 'Bacillus escherichii':ab,ti,kw OR 'Bacterium coli':ab,ti,kw OR 'bacterium E3':ab,ti,kw OR 'coli bacillus':ab,ti,kw OR 'coli bacterium':ab,ti,kw OR colibacillus:ab,ti,kw OR 'colon bacillus':ab,ti,kw OR 'Enterococcus coli':ab,ti,kw OR 'Escherichia alkalescens dispart':ab,ti,kw OR 'Escherichia coli'/exp OR 'Klebsiella pneumoniae':ab,ti,kw OR 'Klebsiella rhinoscleromatis':ab,ti,kw OR 'b. Friedlander':ab,ti,kw OR 'bacillus pneumoniae':ab,ti,kw OR 'Bacterium pneumoniae crouposae':ab,ti,kw OR 'bacterium pneumonie crouposae':ab,ti,kw OR 'friedlaender bacillus':ab,ti,kw OR 'friedlander bacillus':ab,ti,kw OR 'hyalococcus pneumoniae':ab,ti,kw OR 'k. Pneumoniae':ab,ti,kw OR 'klebsiella crouposa':ab,ti,kw OR 'Klebsiella Pn':ab,ti,kw OR 'klebsiella pneumonia':ab,ti,kw OR 'Klebsiella pneumoniae aerogenes':ab,ti,kw OR pneumobacillus:ab,ti,kw OR 'Klebsiella pneumoniae'/exp OR 'Acinetobacter baumannii':ab,ti,kw OR 'Acinetobacter baumannii'/exp OR 'Acinetobacter infection'/exp OR 'Pseudomonas aeruginosa':ab,ti,kw OR 'Pseudomonas pyocyanea':ab,ti,kw OR 'Bacillus aeruginosus':ab,ti,kw OR 'Bacillus pyocyaneus':ab,ti,kw OR 'Bacterium aeruginosum':ab,ti,kw OR 'Bacterium pyocyaneum':ab,ti,kw OR 'blue pus organism':ab,ti,kw OR 'Micrococcus pyocyaneus':ab,ti,kw OR P. Aeruginosa:ab,ti,kw OR 'Pseudomonas polycolor':ab,ti,kw OR 'Pseudomonas pyoceaneus':ab,ti,kw OR 'Pseudomonas pyocyaneus':ab,ti,kw OR 'Pseudomonas aeruginosa'/exp OR 'staphylococcus argenteus':ab,ti,kw AND [2014-2019]/py | 36986 |
| #3 'antimicrobial resistance':ab,ti,kw OR 'antimicrobial testing':ab,ti,kw OR 'Antimicrobial susceptibility':ab,ti,kw OR 'Antibiotic susceptibility*':ab,ti,kw OR 'antibiotic resistance':ab,ti,kw OR 'antibiotic testing':ab,ti,kw OR 'multi-drug resistant':ab,ti,kw OR 'multidrug-resistant':ab,ti,kw OR carbapenem-resistant:ab,ti,kw OR carbapenemase:ab,ti,kw OR methicillin resistant:ab,ti,kw OR vancomycin-resistant:ab,ti,kw OR 'extended-spectrum beta-lactamase':ab,ti,kw OR ESBL:ab,ti,kw OR AMR:ab,ti,kw OR resistan*:ab,ti,kw OR MDR:ab,ti,kw OR XDR:ab,ti,kw OR PDR:ab,ti,kw OR pandrug resistance:ab,ti,kw OR pan-drug resistance:ab,ti,kw OR 'disease resistance'/exp OR 'drug resistance'/de OR 'antibiotic resistance'/exp AND [2014-2019]/py | 450729 |
| #4  #1 AND #2 AND #3 | 36 |

### Nosocomial pathogen search (Embase)

**Date Limitation:** 2014-2019

This search was run on 18 November 2019.

| Search strings | No of hits |
| --- | --- |
| #1 'Point of care test*':ab,ti,kw OR POCT:ab,ti,kw OR 'rapid diagnostic test*':ab,ti,kw OR 'rapid test*':ab,ti,kw OR 'bedside test*':ab,ti,kw OR 'near patient test*':ab,ti,kw OR 'handheld device*':ab,ti,kw OR 'handheld instrument*':ab,ti,kw OR 'portable test*':ab,ti,kw OR 'portable device*':ab,ti,kw OR 'portable instrument*':ab,ti,kw OR 'bedside computing':ab,ti,kw OR 'Infectious disease testing':ab,ti,kw OR 'infectious disease screening*':ab,ti,kw OR 'point of care technolog*':ab,ti,kw OR 'bedside technolog*':ab,ti,kw OR 'point of care system*':ab,ti,kw OR 'point of care system'/exp OR 'point of care testing'/exp AND [2014-2019]/py | 19039 |
| #2 'Streptococcus pneumoniae':ab,ti,kw OR Pneumococc*:ab,ti,kw OR 'Diplococcus pneumoniae':ab,ti,kw OR 'Micrococcus pneumoniae':ab,ti,kw OR 'Streptococcus pneumoniae'/exp OR 'Pneumococcal Infection'/exp OR 'Staphylococcus aureus':ab,ti,kw OR 'micrococcus aureus':ab,ti,kw OR 'microccus pyogenes':ab,ti,kw OR 'Staphylococcus aureus'/exp OR 'Enterococcus faecium':ab,ti,kw OR 'streptococcus faecium':ab,ti,kw OR Enterococcus faecium/exp OR 'Enterococcus faecalis':ab,ti,kw OR 'streptococcus Group D':ab,ti,kw OR 'streptococcus faecalis':ab,ti,kw OR 'enterococcus fecalis':ab,ti,kw OR 'enterococcus l form':ab,ti,kw OR 'enterococcus proteiformis':ab,ti,kw OR 'micrococcus ovalis':ab,ti,kw OR 'micrococcus zymogenes':ab,ti,kw OR 'paraghurt':ab,ti,kw OR 'streptococcus fecalis':ab,ti,kw OR 'streptococcus glycerinaceus':ab,ti,kw OR 'streptococcus liquefaciens':ab,ti,kw OR 'streptococcus ovalis':ab,ti,kw OR 'th 69':ab,ti,kw OR 'Enterococcus faecalis'/exp OR 'Escherichia coli':ab,ti,kw OR 'e coli':ab,ti,kw OR 'e. coli':ab,ti,kw OR 'Alkalescens-Dispar Group':ab,ti,kw OR EAggEC:ab,ti,kw OR 'Bacillus coli':ab,ti,kw OR 'Bacillus escherichii':ab,ti,kw OR 'Bacterium coli':ab,ti,kw OR 'bacterium E3':ab,ti,kw OR 'coli bacillus':ab,ti,kw OR 'coli bacterium':ab,ti,kw OR colibacillus:ab,ti,kw OR 'colon bacillus':ab,ti,kw OR 'Enterococcus coli':ab,ti,kw OR 'Escherichia alkalescens dispart':ab,ti,kw OR Escherichia coli/exp OR 'Klebsiella pneumoniae':ab,ti,kw OR 'Klebsiella rhinoscleromatis':ab,ti,kw OR 'b. Friedlander':ab,ti,kw OR 'bacillus pneumoniae':ab,ti,kw OR 'Bacterium pneumoniae crouposae':ab,ti,kw OR 'bacterium pneumonie crouposae':ab,ti,kw OR 'friedlaender bacillus':ab,ti,kw OR 'friedlander bacillus':ab,ti,kw OR 'hyalococcus pneumoniae':ab,ti,kw OR 'k. Pneumoniae':ab,ti,kw OR 'klebsiella crouposa':ab,ti,kw OR 'Klebsiella Pn':ab,ti,kw OR 'klebsiella pneumonia':ab,ti,kw OR 'Klebsiella pneumoniae aerogenes':ab,ti,kw OR pneumobacillus:ab,ti,kw OR Klebsiella pneumoniae/exp OR 'Acinetobacter baumannii':ab,ti,kw OR 'Acinetobacter baumannii'/exp OR 'Acinetobacter infection'/exp OR 'Pseudomonas aeruginosa':ab,ti,kw OR 'Pseudomonas pyocyanea':ab,ti,kw OR 'Bacillus aeruginosus':ab,ti,kw OR 'Bacillus pyocyaneus':ab,ti,kw OR 'Bacterium aeruginosum':ab,ti,kw OR 'Bacterium pyocyaneum':ab,ti,kw OR 'blue pus organism':ab,ti,kw OR 'Micrococcus pyocyaneus':ab,ti,kw OR 'P. Aeruginosa':ab,ti,kw OR 'Pseudomonas polycolor':ab,ti,kw OR 'Pseudomonas pyoceaneus':ab,ti,kw OR 'Pseudomonas pyocyaneus':ab,ti,kw OR 'Pseudomonas aeruginosa'/exp OR 'Clostridium difficile':ab,ti,kw OR 'Clostridioides difficile':ab,ti,kw or 'c diff':ab,ti,kw OR 'c. diff':ab,ti,kw OR 'c difficile':ab,ti,kw OR 'c. difficile':ab,ti,kw OR 'Pseudomembranous colitis':ab,ti,kw OR 'Clostridium difficile'/exp OR 'Clostridium perfringens'/exp OR 'Clostridium sordellii'/exp OR Candida:ab,ti,kw OR 'C. glabrata':ab,ti,kw OR 'candida glabrata':ab,ti,kw OR 'candida auris':ab,ti,kw OR Candidiasis:ab,ti,kw OR Candidosis:ab,ti,kw OR candidas:ab,ti,kw OR monilia:ab,ti,kw OR monilias:ab,ti,kw OR 'torulopsis utilis':ab,ti,kw OR 'Candida'/exp OR 'candidiasis'/exp OR 'staphylococcus argenteus':ab,ti,kw AND [2014-2019]/py | 96318 |
| #3 'Nosocomial infection*':ab,ti,kw OR 'Healthcare associated infection*':ab,ti,kw OR 'healthcare acquired infection*':ab,ti,kw OR 'Health care associated infection*':ab,ti,kw OR 'health care acquired infection*':ab,ti,kw OR HCAI:ab,ti,kw OR 'Hospital acquired infection*':ab,ti,kw OR HAI:ab,ti,kw OR 'cross infection*':ab,ti,kw OR 'hospital infection*':ab,ti,kw OR 'Cross Infection'/exp AND [2014-2019]/py | 16810 |
| #4 #1 AND #2 AND #3 | 18 |

### General infectious disease search (Embase)

**Date Limitation:** 2014-2019

This search was run on 18 November 2019.

| Search strings | No of hits |
| --- | --- |
| #1 'Point of care test*':ab,ti,kw OR POCT:ab,ti,kw OR 'rapid diagnostic test*':ab,ti,kw OR 'rapid test*':ab,ti,kw OR 'bedside test*':ab,ti,kw OR 'near patient test*':ab,ti,kw OR 'handheld device*':ab,ti,kw OR 'handheld instrument*':ab,ti,kw OR 'portable test*':ab,ti,kw OR 'portable device*':ab,ti,kw OR 'portable instrument*':ab,ti,kw OR 'bedside computing':ab,ti,kw OR 'Infectious disease testing':ab,ti,kw OR 'infectious disease screening*':ab,ti,kw OR 'point of care technolog*':ab,ti,kw OR 'bedside technolog*':ab,ti,kw OR 'point of care system*':ab,ti,kw OR 'point of care system'/exp OR 'point of care testing'/exp AND [2014-2019]/py | 19039 |
| #2 'antimicrobial resistance':ab,ti,kw OR 'antimicrobial testing':ab,ti,kw OR 'Antimicrobial susceptibility':ab,ti,kw OR 'Antibiotic susceptibility*':ab,ti,kw OR 'antibiotic resistance':ab,ti,kw OR 'antibiotic testing':ab,ti,kw OR 'multi-drug resistant':ab,ti,kw OR 'multidrug-resistant':ab,ti,kw OR carbapenem-resistant:ab,ti,kw OR carbapenemase:ab,ti,kw OR 'methicillin resistant':ab,ti,kw OR 'vancomycin-resistant':ab,ti,kw OR 'extended-spectrum beta-lactamase':ab,ti,kw OR ESBL:ab,ti,kw OR AMR:ab,ti,kw OR resistan*:ab,ti,kw OR MDR:ab,ti,kw OR XDR:ab,ti,kw OR PDR:ab,ti,kw OR 'pandrug resistance':ab,ti,kw OR 'pan-drug resistance':ab,ti,kw OR 'disease resistance'/exp OR 'drug resistance'/de OR 'antibiotic resistance'/exp OR 'Nosocomial infection*':ab,ti,kw OR 'Healthcare associated infection*':ab,ti,kw OR 'healthcare acquired infection*':ab,ti,kw OR 'Health care associated infection*':ab,ti,kw OR 'health care acquired infection*':ab,ti,kw OR HCAI:ab,ti,kw OR 'Hospital acquired infection*':ab,ti,kw OR HAI:ab,ti,kw OR 'cross infection*':ab,ti,kw OR 'hospital infection*':ab,ti,kw OR 'Cross Infection'/exp OR 'Infectious disease*':ab,ti,kw OR 'Communicable disease*':ab,ti,kw OR infection*:ab,ti,kw OR 'communicable disease'/exp OR 'Sexually transmitted infection*':ab,ti,kw OR 'Sexually transmitted disease*':ab,ti,kw OR STI:ab,ti,kw OR STD:ab,ti,kw OR 'Venereal disease*':ab,ti,kw OR 'sexually transmitted disease'/exp OR 'Respiratory infection*':ab,ti,kw OR 'Respiratory tract infection*':ab,ti,kw OR 'Pleural Empyema*':ab,ti,kw OR 'Thoracic Empyemas':ab,ti,kw OR Pyothorax:ab,ti,kw OR 'Tuberculous Empyema':ab,ti,kw OR 'respiratory tract infection'/exp OR 'Gastrointestinal infection*':ab,ti,kw OR 'gastrointestinal infection'/exp OR 'vaccine-preventable infection*':ab,ti,kw OR 'vaccine-preventable disease*':ab,ti,kw OR VPD:ab,ti,kw OR VPI:ab,ti,kw OR 'vaccine'/exp OR 'Emerging infection*':ab,ti,kw OR 'Emerging infectious disease*':ab,ti,kw OR 'Emerging communicable disease*':ab,ti,kw OR 're-emerging communicable disease*':ab,ti,kw OR 'reemerging communicable disease*':ab,ti,kw OR 'reemerging infectious disease*':ab,ti,kw OR 're-emerging infectious disease*':ab,ti,kw OR 're-emerging infection*':ab,ti,kw OR 'reemerging infection*':ab,ti,kw OR Sepsis:ab,ti,kw OR Septicemia:ab,ti,kw OR Septicaemia:ab,ti,kw OR 'Septic shock':ab,ti,kw OR 'Bloodstream infection*':ab,ti,kw OR 'Blood infection*':ab,ti,kw OR Pyemia:ab,ti,kw OR Pyemias:ab,ti,kw OR Pyohemia:ab,ti,kw OR Pyohemias:ab,ti,kw OR Pyaemia:ab,ti,kw OR Pyaemias:ab,ti,kw OR 'Blood Poisoning':ab,ti,kw OR Sepsis/exp OR 'Viral meningitis':ab,ti,kw OR 'Bacterial meningitis':ab,ti,kw OR 'Fungal meningitis':ab,ti,kw OR 'Parasitic meningitis':ab,ti,kw OR 'virus meningitis'/exp OR 'fungal meningitis'/exp OR 'bacterial meningitis'/exp OR 'urinary tract infection*':ab,ti,kw OR UTI:ab,ti,kw OR bacteriuria:ab,ti,kw OR pyuria:ab,ti,kw OR 'urinary tract infection'/exp OR 'Bacterial pneumonia*':ab,ti,kw OR 'viral pneumonia*':ab,ti,kw OR 'fungal pneumonia*':ab,ti,kw OR 'Infectious pneumonia*':ab,ti,kw OR 'bacterial pneumonia'/exp OR 'virus pneumonia'/exp OR diarrhoea:ab,ti,kw OR diarrhea:ab,ti,kw OR 'diarrheal disease*':ab,ti,kw OR 'diarrhoeal disease*':ab,ti,kw OR 'diarrhea'/exp AND [2014-2019]/py | 1302318 |
| #3 #1 AND #2 | 6362 |

### Embase search summary

| Search summary | Number of initial results (before de-duplication) | Number of unique results (after de-duplication) |
| --- | --- | --- |
| 56 Diseases | 6150 | 2710 |
| AMR pathogens | 36 | 12 |
| Nosocomial pathogens | 18 | 4 |
| General infectious diseases | 6362 | 1261 |
| **Total** | **12566** | **3987** |

## Scopus search strings and number of search hits

### 56 diseases

**Date Limitation:** 2014-2019

This search was run on 19 November 2019.

| Search strings | No of hits |
| --- | --- |
| #1 TITLE-ABS-KEY ( "Point of care test*" OR POCT OR "rapid diagnostic test*" OR "rapid test*" OR "bedside test*" OR "near patient test*" OR "handheld device*" OR "handheld instrument*" OR "portable test*" OR "portable device*" OR "portable instrument*" OR "bedside computing" OR "Infectious disease testing" OR "infectious disease screening*" OR "point of care technolog*" OR "bedside technolog*" OR "point of care system*") AND PUBYEAR > 2013 | 24810 |
| #2 TITLE-ABS-KEY ( Anthrax OR anthracis ) AND PUBYEAR > 2013 | 2837 |
| #3 #1 AND #2 | 24 |
| #4  TITLE-ABS-KEY ( botulism OR botulinum ) AND PUBYEAR > 2013 | 10150 |
| #5 #1 AND #4 | 22 |
| #6 TITLE-ABS-KEY ( brucellosis OR Brucella OR "b. abortus" OR "b. canis" OR "b. melitensis" OR "b. ovis" OR "b. suis" ) AND PUBYEAR > 2013 | 4429 |
| #7 #1 AND #6 | 41 |
| #8 TITLE-ABS-KEY ( campylobacteriosis OR campylobacter* OR "c.coli" OR "c.fetus" OR "c.hyointestinalis" OR "c.jejuni" OR "c.lari" OR "c.rectus" OR "c.sputorum" OR "c.upsaliensis" ) AND PUBYEAR > 2013 | 5789 |
| #9 #1 AND #8 | 41 |
| #10 TITLE-ABS-KEY ( (chikungunya W/2 (virus OR fever))) OR TITLE-ABS-KEY ( chikv ) ) AND PUBYEAR > 2013 | 3340 |
| #11 #1 AND #10 | 49 |
| #12 TITLE-ABS-KEY ( Chlamydia OR "c.trachomatic" OR "c.pneumoniae" OR "c.psittaci" OR trachoma OR "inclusion conjunctivitis" OR "ophthalmia neonatorum" OR "Lymphogranuloma venereum" OR LGV OR psittacosis) AND PUBYEAR > 2013 | 7504 |
| #13 #1 AND #12 | 202 |
| #14  TITLE-ABS-KEY ( cholera OR "vibrio cholerae" OR "v. cholerae") AND PUBYEAR > 2013 | 7041 |
| #15 #1 AND #14 | 63 |
| #16 TITLE-ABS-KEY ( cryptosporidi* OR "c.parvum" ) AND PUBYEAR > 2013 | 3249 |
| #17 #1 AND #16 | 39 |
| #18 TITLE-ABS-KEY ( dengue OR DENV ) AND PUBYEAR > 2013 | 14180 |
| #19 #1 AND #18 | 294 |
| #20 TITLE-ABS-KEY ( diphtheria OR "Corynebacterium diphtheriae" OR "Klebs-Loffler bacillus" ) AND PUBYEAR > 2013 | 5250 |
| #21 #1 AND #20 | 4 |
| #22 TITLE-ABS-KEY ( echinococc* OR "E. granulosusv" OR "E. multilocularis" OR "Hydatid cyst" OR "Hydatid disease*") AND PUBYEAR > 2013 | 4479 |
| #23 #1 AND #22 | 24 |
| #24 TITLE-ABS-KEY ( giardia* OR Lamblia OR Lamblias OR Lambliasis OR "G. intestinalis" OR "g. duodenalis" OR "g. muris" ) AND PUBYEAR > 2013 | 3350 |
| #25 #1 AND #24 | 49 |
| #26 TITLE-ABS-KEY ( gonorrhoea* OR gonorrhea* OR gonococc* ) AND PUBYEAR > 2013 | 5100 |
| #27 #1 AND #26 | 175 |
| #28 TITLE-ABS-KEY ( "hepatitis A" OR "hep A" OR "hepatitis virus A" OR hav ) AND PUBYEAR > 2013 | 5224 |
| #29 #1 AND #28 | 22 |
| #30 TITLE-ABS-KEY ( "hepatitis B" OR "hep b" OR "hepatitis virus B" OR HBV OR "dane particle" ) AND PUBYEAR > 2013 | 32858 |
| #31 #1 AND #30 | 322 |
| #32 TITLE-ABS-KEY ( "hepatitis C" OR "hep C" OR "hepatitis virus C" OR HCV OR "hepacvirus c" ) AND PUBYEAR > 2013 | 37366 |
| #33 #1 AND #32 | 375 |
| #34 TITLE-ABS-KEY ( HIV OR "human immunodeficiency virus" OR "human immune deficiency virus*" OR "immune deficiency associated virus" OR "immune deficiency associated viruses" OR "immunodeficiency associated virus" OR "immunodeficiency associated viruses" OR "acquired immunodeficiency syndrome" OR "acquired immune deficiency syndrome" OR AIDS ) AND PUBYEAR > 2013 | 138609 |
| #35 #1 AND #34 | 1995 |
| #36 TITLE-ABS-KEY ( "haemophilus influenza type b" OR "hemophilus influenza type b" OR Hib OR "H. influenzae type b" OR "hemophilus influenza group b" OR "haemophilus influenza group b" ) AND PUBYEAR > 2013 | 832 |
| #37 #1 AND #36 | 3 |
| #38  TITLE-ABS-KEY ( flu OR influenza* OR H1N1 ) AND PUBYEAR > 2013 | 48205 |
| #39 #1 AND #38 | 622 |
| #40 TITLE-ABS-KEY ( legionella OR "L. pneumophila" OR (Legionnaire* w/2 disease*) OR "pontiac fever" ) AND PUBYEAR > 2013 | 3094 |
| #41 #1 AND #40 | 41 |
| #42  TITLE-ABS-KEY ( leptospirosis OR Leptospira OR "L. interrogans" OR "L. kirschneri" OR "L. borgpetrsenii" OR "L. santarosai" OR "L. noguchii" OR "L. weilii" OR "L. alexanderi" OR "L. alstoni" OR "L. kmetyi" ) AND PUBYEAR > 2013 | 3183 |
| #43 #1 AND #42 | 81 |
| #44 TITLE-ABS-KEY ( Listerios* OR Listeria* OR "L. monocytogenes" OR "L. seeligeri" OR "L. ivanovii" OR "L. welshimeri" OR "L. grayi" OR "L. innocua" OR "L. marthii" OR "L. rocourtiae" ) AND PUBYEAR > 2013 | 10644 |
| #45 #1 AND #44 | 35 |
| #46 TITLE-ABS-KEY ( (Lyme* w/2 disease) OR Borrelia OR neurobirreliosis ) AND PUBYEAR > 2013 | 5005 |
| #47 #1 AND #46 | 34 |
| #48  TITLE-ABS-KEY ( malaria OR Plasmodium OR "P. falciparum" OR "P. vivax" OR "P. ovale" OR "P. malariae" OR "P. knowlesi" OR Paludism ) AND PUBYEAR > 2013 | 32358 |
| #49  #1 AND #48 | 1557 |
| #50  TITLE-ABS-KEY ( Measles OR Rubeola ) AND PUBYEAR > 2013 | 7101 |
| #51 #1 AND #51 | 22 |
| #52 TITLE-ABS-KEY ( mumps OR parotitis OR "epidemic parotid virus*" OR "epidemic parotiditis virus*" OR "epidemic parotitides virus*" OR "epidemic parotitus virus*" ) AND PUBYEAR > 2013 | 3528 |
| #53 #1 AND #52 | 12 |
| #54 TITLE-ABS-KEY ( "Neisseria meningitidis" OR meningococcus OR meningococc* ) AND PUBYEAR > 2013 | 5059 |
| #55 #1 AND #54 | 39 |
| #56 TITLE-ABS-KEY ( pertussis OR "whooping cough" ) AND PUBYEAR > 2013 | 6204 |
| #57 #1 AND #56 | 24 |
| #58 TITLE-ABS-KEY ( plague* OR "Yersinia pestis" OR "Y. pestis" OR "Black death" ) AND PUBYEAR > 2013 | 8428 |
| #59 #1 AND #58 | 27 |
| #60 TITLE-ABS-KEY ( polio OR poliomyelitis OR poliovirus* OR polioenterovirus* ) AND PUBYEAR > 2013 | 5568 |
| #61 #1 AND #60 | 8 |
| #62 TITLE-ABS-KEY ( "Q fever" OR Coxiella OR "C. burnetii" OR "Abattoir fever" OR Coxiellosis ) AND PUBYEAR > 2013 | 1811 |
| #63 #1 AND #62 | 11 |
| #64  TITLE-ABS-KEY ( rabies ) AND PUBYEAR > 2013 | 3901 |
| #65 #1 AND #64 | 13 |
| #66 TITLE-ABS-KEY ( rubella OR rubellavirus OR RuV OR "German measles" ) AND PUBYEAR > 2013 | 4014 |
| #67 #1 AND #66 | 19 |
| #68 TITLE-ABS-KEY ( salmonellosis OR "Salmonella enterica" OR Salmonella OR "S. Enteritidis" OR "S.Typhimurium" OR "S. Typhi" ) AND PUBYEAR > 2013 | 26672 |
| #69 #1 AND #68 | 182 |
| #70 TITLE-ABS-KEY ( SARS* OR "severe acute respiratory syndrome" OR "severe acute respiratory syndrome-related coronavirus" ) AND PUBYEAR > 2013 | 4431 |
| #71 #1 AND #70 | 18 |
| #72 TITLE-ABS-KEY ( "verocytotoxin-producing Escherichia coli" OR "Shiga toxin-producing Escherichia coli" OR VTEC OR STEC OR "verocytotoxin-producing E. coli" OR "Shiga toxin-producing E. coli" OR EHEC OR SLTEC OR non-O157 OR coli-O157) AND PUBYEAR > 2013 | 6324 |
| #73 #1 AND #72 | 65 |
| #74 TITLE-ABS-KEY ( Shigella* OR shigellosis OR "S. dysenteriae" OR "S. flexneri" OR "S. boydii" OR "S. sonnei" OR "bacillary dysentery" ) AND PUBYEAR > 2013 | 4986 |
| #75 #1 AND #74 | 25 |
| #76 TITLE-ABS-KEY ( smallpox* OR Variola* ) AND PUBYEAR > 2013 | 1470 |
| #77 #1 AND #76 | 8 |
| #78 TITLE-ABS-KEY ( "Streptococcus pneumonia*" OR "S. pneumonia*" OR "diplococcus pneumonia*" OR "d. pneumonia*" OR pneumococcal OR IPD OR pneumococcus ) AND PUBYEAR > 2013 | 17390 |
| #79 #1 AND #78 | 129 |
| #80 TITLE-ABS-KEY ( Syphilis OR "Treponema pallidum" OR "T. pallidum" OR chancre ) AND PUBYEAR > 2013 | 6791 |
| #81 #1 AND #80 | 325 |
| #82 TITLE-ABS-KEY ( tetanus OR "Clostridium tetani" OR "C. tetani" OR "Bacillus tetani" OR "B. tetani" ) AND PUBYEAR > 2013 | 5954 |
| #83 #1 AND #82 | 18 |
| #84 TITLE-ABS-KEY ( "tick borne encephalitis*" OR "tick-borne encephalitis*" OR TBE* ) AND PUBYEAR > 2013 | 2228 |
| #85 #1 AND #84 | 4 |
| #86 TITLE-ABS-KEY ( toxoplasm* ) AND PUBYEAR > 2013 | 7817 |
| #87 #1 AND #86 | 52 |
| #88 TITLE-ABS-KEY ( "transmissible spongiform encephalopath*" OR TSEs OR "Bovine spongiform encephalopath*" OR "Mad cow disease" ) AND PUBYEAR > 2013 | 1083 |
| #89 #1 AND #88 | 11 |
| #90 TITLE-ABS-KEY ( trichina* OR trichinellosis OR trichinosis OR Trichinella* OR "t. britovi" OR "t. murrelli" OR "t. nativa" OR "t. nelson" OR "t. spiralis" OR "t. papuae" OR "t. pseudospiralis" OR "t. zimbabwensis" ) AND PUBYEAR > 2013 | 922 |
| #91 #1 AND #90 | 4 |
| #92 TITLE-ABS-KEY ( tuberculosis OR MTB OR LTBI OR "koch’s disease" OR "m. africanum" OR "m. canetti" OR "m. caprae" OR "m. orygis" ) AND PUBYEAR > 2013 | 57650 |
| #93 #1 AND #92 | 962 |
| #94 TITLE-ABS-KEY ( typhoid OR paratyphoid OR "Salmonella Typhi" OR "Salmonella Paratyphi" OR "salmonella schottmuelleri" OR "salmonella hirschfeldii" OR "S. Typhi" OR "S. Paratyphi" OR "s. schottmuelleri" OR "s. hirschfeldii" OR "Enteric fever" ) AND PUBYEAR > 2013 | 5064 |
| #95 #1 AND #94 | 84 |
| #96 TITLE-ABS-KEY ( tularemia OR tularaemia OR "Francisella tularensis" OR "pasteurella tularensis" OR "bacterium tularense" ) AND PUBYEAR > 2013 | 1297 |
| #97 #1 AND #96 | 12 |
| #98 TITLE-ABS-KEY ( (prion OR prions) AND ((variant) AND (Creutzfeldt-Jakob* OR CJD)) OR (vCJD OR "variant cjd" OR v-cjd OR vcjd)) AND PUBYEAR > 2013 | 298 |
| #99 #1 AND #98 | 2 |
| #100 TITLE-ABS-KEY ( "viral hemorrhagic fever*" OR "viral haemorrhagic fever*" OR arenavirus OR filovirus OR ebola* OR EBOV OR Lassa OR "Marburg virus" OR Marburgvirus OR lassavirus OR marv OR hantavirus OR "hanta virus" OR "junin virus" OR "Machupo mammarenavirus" OR nairovirus OR "Crimean-congo hemorrhagic fever virus" OR CCHF* OR "zaire ebolavirus" OR "omsk hemorrhagic fever virus" OR OHFV OR "kyasanur forest disease virus" OR "rift valley fever virus" OR RVF ) AND PUBYEAR > 2013 | 12934 |
| #101 #1 AND #100 | 221 |
| #102  TITLE-ABS-KEY ( ("west nile" W/2 (fever OR virus))) OR TITLE-ABS-KEY ( "West Nile flavivirus" OR "egypt 101 virus" OR "kunjin virus" OR WNV ) AND PUBYEAR > 2013 | 3256 |
| #103 #1 AND #102 | 17 |
| #104 TITLE-ABS-KEY ( "Yellow fever" OR YFV) AND PUBYEAR > 2013 | 2814 |
| #105 #1 AND #104 | 24 |
| #106 TITLE-ABS-KEY ( Yersiniosis OR "Yersinia enterocolitica" OR "Y. enterocolitica" OR "Y. pseudotuberculosis" ) AND PUBYEAR > 2013 | 1418 |
| #107 #1 AND #106 | 8 |
| #108 TITLE-ABS-KEY ( Zika OR Zikas OR Zikv OR Zikav OR "congenial zika" ) AND PUBYEAR > 2013 | 8500 |
| #109 #1 AND #108 | 102 |
| #110 #3 OR #5 OR #7 OR #9 OR #11 OR #13 OR #15 OR #17 OR #19 OR #21 OR #23 OR #25 OR #27 OR #29 OR #31 OR #33 OR #35 OR #37 OR #39 OR #41 OR #43 OR #45 OR #47 OR #49 OR #51 OR #53 OR #55 OR #57 OR #59 OR #61 OR #63 OR #65 OR #67 OR #69 OR #71 OR #73 OR #75 OR #77 OR #79 OR #81 OR #83 OR #85 OR #87 OR #91 OR #93 OR #95 OR #97 OR #99 OR #101 OR #103 OR #105 OR #107 OR #109 | 5947 |

### AMR pathogen search (Scopus)

**Date Limitation:** 2014-2019

This search was run on 20 November 2019.

| Search strings | No of hits |
| --- | --- |
| #1 TITLE-ABS-KEY ( "Point of care test*" OR POCT OR "rapid diagnostic test*" OR "rapid test*" OR "bedside test*" OR "near patient test*" OR "handheld device*" OR "handheld instrument*" OR "portable test*" OR "portable device*" OR "portable instrument*" OR "bedside computing" OR "Infectious disease testing" OR "infectious disease screening*" OR "point of care technolog*" OR "bedside technolog*" OR "point of care system*") AND PUBYEAR > 2013 | 24817 |
| #2 TITLE-ABS-KEY ( "Staphylococcus aureus" OR "micrococcus aureus" OR "microccus pyogenes" ) OR TITLE-ABS-KEY ( "Enterococcus faecium" OR "streptococcus faecium" ) OR TITLE-ABS-KEY ( "Enterococcus faecalis" OR "streptococcus Group D" OR "streptococcus faecalis" OR "enterococcus fecalis" OR "enterococcus l form" OR "enterococcus proteiformis" OR "micrococcus ovalis" OR "micrococcus zymogenes" OR paraghurt OR "streptococcus fecalis" OR "streptococcus glycerinaceus" OR "streptococcus liquefaciens" OR "streptococcus ovalis" OR "th 69" ) OR TITLE-ABS-KEY ( "Escherichia coli" OR "e coli" OR "e. coli" OR "Alkalescens-Dispar Group" OR EAggEC OR "Bacillus coli" OR "Bacillus escherichii" OR "Bacterium coli" OR "bacterium E3" OR "coli bacillus" OR "coli bacterium" OR colibacillus OR "colon bacillus" OR "Enterococcus coli" OR "Escherichia alkalescens dispart" ) OR TITLE-ABS-KEY ( "Klebsiella pneumoniae" OR "Klebsiella rhinoscleromatis" OR "b. Friedlander" OR "bacillus pneumoniae" OR "Bacterium pneumoniae crouposae" OR "bacterium pneumonie crouposae" OR "friedlaender bacillus" OR "friedlander bacillus" OR "hyalococcus pneumoniae" OR "k. Pneumoniae" OR "klebsiella crouposa" OR "Klebsiella Pn" OR "klebsiella pneumonia" OR "Klebsiella pneumoniae aerogenes" OR pneumobacillus ) OR TITLE-ABS-KEY ( "Acinetobacter baumannii" ) OR TITLE-ABS-KEY ( "Pseudomonas aeruginosa" OR "Pseudomonas pyocyanea" OR "Bacillus aeruginosus" OR "Bacillus pyocyaneus" OR "Bacterium aeruginosum" OR "Bacterium pyocyaneum" OR "blue pus organism" OR "Micrococcus pyocyaneus" OR "P. Aeruginosa" OR "Pseudomonas polycolor" OR "Pseudomonas pyoceaneus" OR "Pseudomonas pyocyaneus") OR TITLE-ABS-KEY ( "staphylococcus argenteus" ) AND PUBYEAR > 2013 | 190118 |
| #3  TITLE-ABS-KEY ( "antimicrobial resistance" OR "antimicrobial testing" OR "Antimicrobial susceptibility" OR "Antibiotic susceptibility*" OR "antibiotic resistance" OR "antibiotic testing" OR "multi-drug resistant" OR '"multidrug-resistant" OR carbapenem-resistant OR carbapenemase OR "methicillin resistant" OR vanomycin-resistant OR "extended-spectrum beta-lactamase" OR ESBL OR AMR OR resistan* OR MDR OR XDR OR PDR OR pandrug resistance OR pan-drug resistance | 61612 |
| #4  #1 AND #2 AND #3 | 133 |

### Nosocomial pathogen search (Scopus)

**Date Limitation:** 2014-2019

This search was run on 20 November 2019.

| Search strings | No of hits |
| --- | --- |
| #1 TITLE-ABS-KEY ( "Point of care test*" OR POCT OR "rapid diagnostic test*" OR "rapid test*" OR "bedside test*" OR "near patient test*" OR "handheld device*" OR "handheld instrument*" OR "portable test*" OR "portable device*" OR "portable instrument*" OR "bedside computing" OR "Infectious disease testing" OR "infectious disease screening*" OR "point of care technolog*" OR "bedside technolog*" OR "point of care system*") AND PUBYEAR > 2013 | 24817 |
| #2 TITLE-ABS-KEY ( "Streptococcus pneumoniae" OR Pneumococc* OR "Diplococcus pneumoniae" OR "Micrococcus pneumoniae" ) OR TITLE-ABS-KEY ( "Staphylococcus aureus" OR "micrococcus aureus" OR "microccus pyogenes" ) OR TITLE-ABS-KEY ( "Enterococcus faecium" OR "streptococcus faecium" ) OR  TITLE-ABS-KEY ( "Enterococcus faecalis" OR "streptococcus Group D" OR "streptococcus faecalis" OR "enterococcus fecalis" OR "enterococcus l form" OR "enterococcus proteiformis" OR "micrococcus ovalis" OR "micrococcus zymogenes" OR paraghurt OR "streptococcus fecalis" OR "streptococcus glycerinaceus" OR "streptococcus liquefaciens" OR "streptococcus ovalis" OR "th 69" ) OR TITLE-ABS-KEY ( "Escherichia coli" OR "e coli" OR "e. coli" OR "Alkalescens-Dispar Group" OR EAggEC OR "Bacillus coli" OR "Bacillus escherichii" OR "Bacterium coli" OR "bacterium E3" OR "coli bacillus" OR "coli bacterium" OR colibacillus OR "colon bacillus" OR "Enterococcus coli" OR "Escherichia alkalescens dispart" ) OR TITLE-ABS-KEY ( "Klebsiella pneumoniae" OR "Klebsiella rhinoscleromatis" OR "b. Friedlander" OR "bacillus pneumoniae" OR "Bacterium pneumoniae crouposae" OR "bacterium pneumonie crouposae" OR "friedlaender bacillus" OR "friedlander bacillus" OR "hyalococcus pneumoniae" OR "k. Pneumoniae" OR "klebsiella crouposa" OR "Klebsiella Pn" OR "klebsiella pneumonia" OR "Klebsiella pneumoniae aerogenes" OR pneumobacillus ) OR TITLE-ABS-KEY ( "Acinetobacter baumannii" ) OR TITLE-ABS-KEY ( "Pseudomonas aeruginosa" OR "Pseudomonas pyocyanea" OR "Bacillus aeruginosus" OR "Bacillus pyocyaneus" OR "Bacterium aeruginosum" OR "Bacterium pyocyaneum" OR "blue pus organism" OR "Micrococcus pyocyaneus" OR "P. Aeruginosa" OR "Pseudomonas polycolor" OR "Pseudomonas pyoceaneus" OR "Pseudomonas pyocyaneus" ) OR TITLE-ABS-KEY ( "Clostridium difficile" OR "Clostridioides difficile" or "c diff" OR "c. diff" OR "c difficile" OR "c. difficile" OR "Pseudomembranous colitis" ) OR TITLE-ABS-KEY ( Candida OR "C. glabrata" OR "candida glabrata" OR "candida auris" OR Candidiasis OR Candidosis OR candidas OR monilia OR monilias OR "torulopsis utilis" ) OR TITLE-ABS-KEY ( "staphylococcus argenteus" ) AND PUBYEAR > 2013 | 232932 |
| #3 TITLE-ABS-KEY ( "Nosocomial infection*" OR "Healthcare associated infection*" OR "healthcare acquired infection*" OR "Health care associated infection*" OR "health care acquired infection*" OR HCAI OR "Hospital acquired infection*" OR HAI OR "cross infection*" OR "hospital infection*" ) AND PUBYEAR > 2013 | 23369 |
| #4 #1 AND #2 AND #3 | 60 |

### General infectious disease search (Scopus)

**Date Limitation:** 2014-2019

This search was run on 20 November 2019.

| Search strings | No of hits |
| --- | --- |
| #1 TITLE-ABS-KEY ( "Point of care test*" OR POCT OR "rapid diagnostic test*" OR "rapid test*" OR "bedside test*" OR "near patient test*" OR "handheld device*" OR "handheld instrument*" OR "portable test*" OR "portable device*" OR "portable instrument*" OR "bedside computing" OR "Infectious disease testing" OR "infectious disease screening*" OR "point of care technolog*" OR "bedside technolog*" OR "point of care system*") AND PUBYEAR > 2013 | 24817 |
| #2  TITLE-ABS-KEY ( "antimicrobial resistance" OR "antimicrobial testing" OR "Antimicrobial susceptibility" OR "Antibiotic susceptibility*" OR "antibiotic resistance" OR "antibiotic testing" OR "multi-drug resistant" OR '"multidrug-resistant" OR carbapenem-resistant OR carbapenemase OR "methicillin resistant" OR vanomycin-resistant OR "extended-spectrum beta-lactamase" OR ESBL OR AMR OR resistan* OR MDR OR XDR OR PDR OR "pandrug resistance" OR "pan-drug resistance") OR TITLE-ABS-KEY ( "Nosocomial infection*" OR "Healthcare associated infection*" OR "healthcare acquired infection*" OR "Health care associated infection*" OR "health care acquired infection*" OR HCAI OR "Hospital acquired infection*" OR HAI OR "cross infection*" OR "hospital infection*" ) OR TITLE-ABS-KEY ( "Infectious disease*" OR "Communicable disease*" OR infection* ) OR TITLE-ABS-KEY ( "Sexually transmitted infection*" OR "Sexually transmitted disease*" OR STI OR STD OR "Venereal disease*" ) OR TITLE-ABS-KEY ( "Respiratory infection*'" OR "Respiratory tract infection*" OR "Pleural Empyema*" OR "Thoracic Empyemas" OR Pyothorax OR "Tuberculous Empyema" ) OR TITLE-ABS-KEY ( "Gastrointestinal infection*" ) OR TITLE-ABS-KEY ( "vaccine-preventable infection*" OR "vaccine-preventable disease*" OR VPD OR VPI ) OR TITLE-ABS-KEY ( "Emerging infection*" OR "Emerging infectious disease*" OR "Emerging communicable disease*" OR "re-emerging communicable disease*" OR "reemerging communicable disease*" OR "reemerging infectious disease*" OR "re-emerging infectious disease*" OR "re-emerging infection*" OR "reemerging infection*" ) OR TITLE-ABS-KEY ( Sepsis OR Septicemia OR Septicaemia OR "Septic shock" OR "Bloodstream infection*" OR "Blood infection*" OR Pyemia OR Pyemias OR Pyohemia OR Pyohemias OR Pyaemia OR Pyaemias OR "Blood Poisoning" ) OR TITLE-ABS-KEY ( "Viral meningitis" OR "Bacterial meningitis" OR "Fungal meningitis" OR "Parasitic meningitis" ) OR TITLE-ABS-KEY ( "urinary tract infection*" OR UTI OR bacteriuria OR pyuria ) OR TITLE-ABS-KEY ( "Bacterial pneumonia*" OR "viral pneumonia*" OR "fungal pneumonia*" OR "Infectious pneumonia*" ) OR TITLE-ABS-KEY ( diarrhoea OR diarrhea OR "diarrheal disease*" OR "diarrhoeal disease*" ) AND PUBYEAR > 2013 | 783208 |
| #3 #1 AND #2 | 7072 |

### Scopus search summary

| Search summary | Number of initial results (before de-duplication) | Number of unique results (after de-duplication) |
| --- | --- | --- |
| 56 Diseases | 5947 | 1423 |
| AMR pathogens | 133 | 22 |
| Nosocomial pathogens | 60 | 11 |
| General infectious diseases | 7072 | 822 |
| **Total** | **13212** | **2278** |

## Cochrane search strings and search hits

### 56 diseases

This search was run on 21 November 2019.

| Search strings | No of hits |
| --- | --- |
| #1 ("Point of care test*" OR POCT OR "rapid diagnostic test*" OR "rapid test*" OR "bedside test*" OR "near patient test*" OR "handheld device*" OR "handheld instrument*" OR "portable test*" OR "portable device*" OR "portable instrument*" OR "bedside computing" OR "Infectious disease testing" OR "infectious disease screening*" OR "point of care technolog*" OR "bedside technolog*" OR "point of care system*"):ti,ab,kw OR ([mh "point-of-care systems"] OR [mh "point-of-care testing"]) | 1096 |
| #2 Anthrax:ti,ab,kw OR anthracis:ti,ab,kw OR [mh Anthrax] OR [mh “Bacillus anthracis”] | 70 |
| #3  #1 AND #2 | 0 |
| #4 botulism:ti,ab,kw OR botulinum:ti,ab,kw OR [mh botulism] OR [mh “Clostridium botulinum”] | 2655 |
| #5 #1 AND #4 | 0 |
| #6 brucellosis:ti,ab,kw OR Brucella:ti,ab,kw OR “b. abortus”:ti,ab,kw OR “b.canis”:ti,ab,kw OR “b. melitensis”:ti,ab,kw OR “b.ovis”:ti,ab,kw OR “b. suis”:ti,ab,kw OR [mh brucellosis] OR [mh Brucella] | 159 |
| #7 #1 AND #6 | 1 |
| #8 (campylobacteriosis OR campylobacter* OR "c.coli" OR "c.fetus" OR "c.hyointestinalis" OR "c.jejuni" OR "c.lari" OR "c.rectus" OR "c.sputorum" OR "c.upsaliensis"):ti,ab,kw OR [mh "Campylobacter"] OR [mh “Campylobacter infections“] | 119 |
| #9  #1 AND #8 | 3 |
| #10 (chikungunya NEAR/2 (virus OR fever)):ti,ab,kw OR chikv:ti,ab,kw OR [mh “chikungunya virus”] OR [mh chikungunya] | 71 |
| #11 #1 AND #10 | 0 |
| #12 Chlamydia:ti,ab,kw OR “c.trachomatis”:ti,ab,kw OR “c.pneumoniae”:ti,ab,kw OR “c.psittaci”:ti,ab,kw OR trachoma:ti,ab,kw OR “inclusion conjunctivitis”:ti,ab,kw OR “ophthalmia neonatorum”:ti,ab,kw OR “Lymphogranuloma venereum”:ti,ab,kw OR LGV:ti,ab,kw OR psittacosis:ti,ab,kw OR [mh “Chlamydia infections”] OR [mh “Chlamydia trachomatis”] | 902 |
| #13 #1 AND #12 | 17 |
| #14 (cholera OR "vibrio cholerae" OR "v. cholerae"):ti,ab,kw OR [mh cholera] OR [mh “vibrio cholerae”] | 223 |
| #15 #1 AND #14 | 1 |
| #16 cryptosporidi*:ti,ab,kw OR “c.parvum”:ti,ab,kw OR [mh cryptosporidiosis] OR [mh cryptosporidium] | 105 |
| #17 #1 AND #16 | 4 |
| #18 dengue:ti,ab,kw OR DENV:ti,ab,kw OR [mh dengue] OR [mh “Dengue virus”] | 542 |
| #19 #1 AND #17 | 15 |
| #20 diphtheria:ti,ab,kw OR “Corynebacterium diphtheriae”:ti,ab,kw OR “Klebs Loffler bacillus”:ti,ab,kw OR [mh diphtheria] OR [mh “Corynebacterium diphtheriae”] OR [mh “Corynebacterium infection”] | 1003 |
| #21 #1 AND #20 | 0 |
| #22 echinococc*:ti,ab,kw OR “E. granulosus”:ti,ab,kw OR “Hydatid cyst”:ti,ab,kw OR “Hydatid disease*”:ti,ab,kw OR [mh “Echinococcus”] OR [mh “echinococcosis”] | 54 |
| #23  #1 AND #22 | 0 |
| #24 giardia*:ti,ab,kw OR Lamblia:ti,ab,kw OR Lamblias:ti,ab,kw OR Lambliasis:ti,ab,kw OR “G. intestinalis”:ti,ab,kw OR “g. duodenalis”:ti,ab,kw OR “g. muris”:ti,ab,kw OR [mh giardia] OR [mh giardiasis] | 122 |
| #25 #1 AND #24 | 3 |
| #26 gonorrhoea*:ti,ab,kw OR gonorrhea*:ti,ab,kw OR gonococc*:ti,ab,kw OR “n. gonorrhoeae”:ti,ab,kw OR [mh gonorrhea] OR [mh “Neisseria gonorrhoeae”] | 518 |
| #27 #1 AND #26 | 13 |
| #28 “hepatitis A”:ti,ab,kw OR “hep A”:ti,ab,kw OR "hepatitis virus A":ti,ab,kw OR hav:ti,ab,kw OR [mh “hepatitis A”] OR [mh "Hepatitis A virus"] | 1333 |
| #29 #1 AND #28 | 2 |
| #30 “hepatitis B”:ti,ab,kw OR “hep b”:ti,ab,kw OR "hepatitis virus B":ti,ab,kw OR HBV:ti,ab,kw OR "dane particle":ti,ab,kw OR [mh “hepatitis B”] OR [mh "Hepatitis B virus"] | 9178 |
| #31 #1 AND #30 | 20 |
| #32  ("hepatitis C" OR "hep C" OR "hepatitis virus C" OR HCV OR "hepacvirus c" OR “hepatitis C”):ti,ab,kw OR “hep C”:ti,ab,kw OR "hepatitis virus C":ti,ab,kw OR HCV:ti,ab,kw OR "hepacvirus c":ti,ab,kw OR [mh “hepatitis C”] OR [mh hepacivirus] | 5804 |
| #33 #1 AND #32 | 33 |
| #34 HIV:ti,ab,kw OR “human immunodeficiency virus”:ti,ab,kw OR "human immune deficiency virus*":ti,ab,kw OR "immune deficiency associated viruses":ti,ab,kw OR "immunodeficiency associated virus":ti,ab,kw OR "immunodeficiency associated viruses":ti,ab,kw OR “acquired immunodeficiency syndrome”:ti,ab,kw OR “acquired immune deficiency syndrome”:ti,ab,kw OR AIDS:ti,ab,kw OR [mh “Human immunodeficiency virus”] OR [mh “acquired immune deficiency syndrome”] | 18946 |
| #35 #1 AND #34 | 181 |
| #36 “haemophilus influenzae type b”:ti,ab,kw OR "hemophilus influenzae type b":ti,ab,kw OR Hib:ti,ab,kw OR "H. influenzae type b":ti,ab,kw OR "hemophilus influenza group b":ti,ab,kw OR "haemophilus influenza group b":ti,ab,kw OR [mh “Haemophilus influenzae type b”] | 592 |
| #37 #1 AND #36 | 0 |
| #38 flu:ti,ab,kw OR influenza*:ti,ab,kw OR H1N1:ti,ab,kw OR [mh "Influenza, Human"] OR [mh "influenza in birds"] OR [mh "Influenza A Virus, H1N1 Subtype"] | 6426 |
| #39 #1 AND #38 | 48 |
| #40 legionella:ti,ab,kw OR "L. pneumophila":ti,ab,kw OR (Legionnaire* near/2 disease*):ti,ab,kw OR "pontiac fever":ti,ab,kw OR [mh “legionella pneumophila”] OR [mh “legionnaire disease”] | 71 |
| #41 #1 AND #40 | 0 |
| #42  leptospirosis:ti,ab,kw OR Leptospira:ti,ab,kw OR “L. interrogans”:ti,ab,kw OR “L. kirschneri”:ti,ab,kw OR “L. borgpetersenii”:ti,ab,kw OR “L. santarosai”:ti,ab,kw OR “L. noguchii”:ti,ab,kw OR “L. weilii”:ti,ab,kw OR “L. alexanderi”:ti,ab,kw OR “L. alstoni”:ti,ab,kw OR “L. kmetyi”:ti,ab,kw OR [mh “Leptospira”] OR [mh “Leptospirosis”] | 7 |
| #43 #1 AND #42 | 2 |
| #44 Listerios*:ti,ab,kw OR Listeria*:ti,ab,kw OR “L. monocytogenes“:ti,ab,kw OR “L. seeligeri”:ti,ab,kw OR “L. ivanovii”:ti,ab,kw OR “L. welshimeri”:ti,ab,kw OR “L. grayi”:ti,ab,kw OR “L. innocua”:ti,ab,kw OR “L. marthii”:ti,ab,kw OR “L. rocourtiae”:ti,ab,kw OR [mh “Listeria”] OR [mh “Listeriosis”] | 88 |
| #45 #1 AND #44 | 1 |
| #46 (Lyme* near/2 disease):ti,ab,kw OR Borrelia:ti,ab,kw OR neurobirreliosis:ti,ab,kw OR [mh “Lyme disease”] OR [mh “Borrelia burgdorferi”] | 58 |
| #47 #1 AND #46 | 0 |
| #48 malaria:ti,ab,kw OR Plasmodium:ti,ab,kw OR “P. falciparum”:ti,ab,kw OR “P. vivax”:ti,ab,kw OR “P. ovale”:ti,ab,kw OR “P. malariae”:ti,ab,kw OR "P. knowlesi":ti,ab,kw OR Paludism:ti,ab,kw OR [mh Malaria] OR [mh Plasmodium] | 3604 |
| #49 #1 AND #48 | 221 |
| #50 Measles:ti,ab,kw OR Rubeola:ti,ab,kw OR [mh Measles] OR [mh “Measles virus”] OR [mh morbillivirus] | 548 |
| #51 #1 AND #50 | 0 |
| #52 Mumps:ti,ab,kw OR parotitis:ti,ab,kw OR "epidemic parotid virus*":ti,ab,kw OR "epidemic parotiditis virus*":ti,ab,kw OR "epidemic parotitides virus*":ti,ab,kw OR "epidemic parotitus virus*":ti,ab,kw OR [mh mumps] OR [mh “mumps virus”] | 313 |
| #53 #1 AND #52 | 0 |
| #54  "Neisseria meningitidis":ti,ab,kw OR meningococcus:ti,ab,kw OR meningococc*:ti,ab,kw OR [mh "meningitis, bacterial"] OR [mh "meningitis, meningococcal"] OR [mh "Neisseria meningitidis"] | 814 |
| #55 #1 AND #54 | 5 |
| #56 pertussis:ti,ab,kw OR "whooping cough":ti,ab,kw OR [mh "Whooping Cough"] OR [mh “Bordetella pertussis”] OR [mh “Pertussis Toxin”] | 954 |
| #57 #1 AND #56 | 0 |
| #58 plague*:ti,ab,kw OR "Yersinia pestis":ti,ab,kw OR "Y. pestis":ti,ab,kw OR "Black death":ti,ab,kw OR [mh plague] OR [mh Yersinia] OR [mh "Yersinia infections"] OR [mh "Yersinia pestis"] | 158 |
| #59 #1 AND #58 | 0 |
| #60 polio:ti,ab,kw OR poliomyelitis:ti,ab,kw OR poliovirus*:ti,ab,kw OR polioenterovirus*:ti,ab,kw OR [mh poliomyelitis] OR [mh poliovirus] | 805 |
| #61  #1 AND #60 | 0 |
| #62 "Q fever":ti,ab,kw OR Coxiella:ti,ab,kw OR "C. burnetii":ti,ab,kw OR "Abattoir fever":ti,ab,kw OR Coxiellosis:ti,ab,kw OR [mh "Q fever"] OR [mh coxiella] | 27 |
| #63 #1 AND #62 | 0 |
| #64 rabies:ti,ab,kw OR [mh rabies] OR [mh "rabies virus"] | 221 |
| #65 #1 AND #64 | 0 |
| #66 rubella:ti,ab,kw OR rubellavirus:ti,ab,kw OR RuV:ti,ab,kw OR "German measles":ti,ab,kw OR [mh rubella] OR [mh "rubella virus"] | 347 |
| #67 #1 AND #66 | 0 |
| #68 salmonellosis:ti,ab,kw OR "Salmonella enterica":ti,ab,kw OR Salmonella:ti,ab,kw OR "S. Enteritidis":ti,ab,kw OR "S.Typhimurium":ti,ab,kw OR "S. Typhi":ti,ab,kw OR [mh "Salmonella enterica"] OR [mh “Salmonella infections”] OR [mh "Salmonella"] | 359 |
| #69 #1 AND #68 | 4 |
| #70 SARS*:ti,ab,kw OR "severe acute respiratory syndrome":ti,ab,kw OR "severe acute respiratory syndrome-related coronavirus":ti,ab,kw OR [mh "Severe Acute Respiratory Syndrome"] OR [mh "SARS virus"] | 130 |
| #71 #1 AND #70 | 0 |
| #72 "verocytotoxin-producing Escherichia coli":ti,ab,kw OR "Shiga toxin-producing Escherichia coli":ti,ab,kw OR VTEC:ti,ab,kw OR STEC:ti,ab,kw OR "verocytotoxin-producing E. coli":ti,ab,kw OR "Shiga toxin-producing E. coli":ti,ab,kw OR EHEC:ti,ab,kw OR SLTEC:ti,ab,kw OR non-O157:ti,ab,kw OR coli-O157:ti,ab,kw OR [mh "Shiga-Toxigenic Escherichia coli"] | 39 |
| #73 #1 AND #72 | 1 |
| #74 Shigella*:ti,ab,kw OR shigellosis:ti,ab,kw OR "S. dysenteriae":ti,ab,kw OR "S. flexneri":ti,ab,kw OR "S. boydii":ti,ab,kw OR "S. sonnei":ti,ab,kw OR "bacillary dysentery":ti,ab,kw OR [mh Shigella] OR [mh "Dysentery, Bacillary"] | 136 |
| #75 #1 AND #74 | 2 |
| #76 smallpox*:ti,ab,kw OR Variola*:ti,ab,kw OR [mh smallpox] OR [mh “variola virus”] | 88 |
| #77 #1 AND #75 | 0 |
| #78 "Streptococcus pneumonia*":ti,ab,kw OR "S. pneumonia*":ti,ab,kw OR "diplococcus pneumonia*":ti,ab,kw OR "d. pneumonia*":ti,ab,kw OR pneumococcus:ti,ab,kw OR pneumococcal:ti,ab,kw OR IPD:ti,ab,kw OR [mh "Streptococcus pneumoniae"] OR [mh "Pneumococcal Infections"] | 1170 |
| #79 #1 AND #76 | 6 |
| #80 Syphilis:ti,ab,kw OR "Treponema pallidum":ti,ab,kw OR "T. pallidum":ti,ab,kw OR chancre:ti,ab,kw OR [mh syphilis] OR [mh "Treponema pallidum"] | 413 |
| #81 #1 AND #80 | 21 |
| #82 tetanus:ti,ab,kw OR "Clostridium tetani":ti,ab,kw OR "C. tetani":ti,ab,kw OR "Bacillus tetani":ti,ab,kw OR "B. tetani":ti,ab,kw OR [mh tetanus] OR [mh "Clostridium tetani"] | 1221 |
| #83 #1 AND #82 | 3 |
| #84 "tick borne encephalitis*":ti,ab,kw OR "tick-borne encephalitis*":ti,ab,kw OR TBE*:ti,ab,kw OR [mh "Encephalitis, Tick-Borne"] OR [mh "Encephalitis Viruses, Tick-Borne"] | 169 |
| #85 #1 AND #84 | 4 |
| #86 toxoplasm*:ti,ab,kw OR [mh Toxoplasma] OR [mh "Toxoplasmosis, Congenital"] | 155 |
| #87 #1 AND #86 | 0 |
| #88 "transmissible spongiform encephalopath*":ti,ab,kw OR TSEs:ti,ab,kw OR "Bovine spongiform encephalopath*":ti,ab,kw OR "Mad cow disease":ti,ab,kw OR [mh "Encephalopathy, Bovine Spongiform"] | 13 |
| #89 #1 AND 88 | 0 |
| #90 trichina*:ti,ab,kw OR trichinellosis:ti,ab,kw OR trichinosis:ti,ab,kw OR Trichinella*:ti,ab,kw OR "t. britovi":ti,ab,kw OR "t. murrelli":ti,ab,kw OR "t. nativa":ti,ab,kw OR "t. nelson":ti,ab,kw OR "t. spiralis":ti,ab,kw OR "t. papuae":ti,ab,kw OR "t. pseudospiralis":ti,ab,kw OR "t. zimbabwensis":ti,ab,kw OR [mh Trichinellosis] OR [mh Trichinella] | 0 |
| #91 #1 AND #90 | 0 |
| #92 tuberculosis:ti,ab,kw OR MTB:ti,ab,kw OR LTBI:ti,ab,kw OR "koch’s disease":ti,ab,kw OR "m. africanum":ti,ab,kw OR "m. canetti":ti,ab,kw OR "m. caprae":ti,ab,kw OR "m. orygis":ti,ab,kw OR [mh tuberculosis] OR [mh "Mycobacterium tuberculosis"] | 4102 |
| #93 #1 AND #92 | 52 |
| #94 typhoid:ti,ab,kw OR paratyphoid:ti,ab,kw OR "Salmonella Typhi":ti,ab,kw OR "Salmonella Paratyphi":ti,ab,kw OR "salmonella schottmuelleri":ti,ab,kw OR "salmonella hirschfeldii":ti,ab,kw OR "S. Typhi":ti,ab,kw OR "S. Paratyphi":ti,ab,kw OR "s. schottmuelleri":ti,ab,kw OR "s. hirschfeldii":ti,ab,kw OR "Enteric fever":ti,ab,kw OR [mh "Typhoid fever"] OR [mh "paratyphoid fever"] | 326 |
| #95 #1 AND #94 | 4 |
| #96 tularemia:ti,ab,kw OR tularaemia:ti,ab,kw OR "Francisella tularensis":ti,ab,kw OR "pasteurella tularensis":ti,ab,kw OR "bacterium tularense":ti,ab,kw OR [mh tularemia] OR [mh "Francisella tularensis"] | 7 |
| #97 #1 AND #96 | 0 |
| #98 ((prion OR prions):ti,ab,kw AND ((variant) AND ("Creutzfeldt-Jakob*" OR CJD OR [mh "Creutzfeldt-Jakob Syndrome"]):ti,ab,kw)) OR ((prion OR prions):ti,ab,kw AND (vCJD OR "variant cjd" OR v-cjd OR vcjd)):ti,ab,kw | 3 |
| #99 #1 AND #98 | 0 |
| #100 "viral hemorrhagic fever*":ti,ab,kw OR "viral haemorrhagic fever*":ti,ab,kw OR arenavirus:ti,ab,kw OR filovirus:ti,ab,kw OR ebola*:ti,ab,kw OR EBOV:ti,ab,kw OR Lassa OR "Marburg virus":ti,ab,kw OR Marburgvirus:ti,ab,kw OR lassavirus:ti,ab,kw OR marv:ti,ab,kw OR hantavirus:ti,ab,kw OR "hanta virus":ti,ab,kw OR "junin virus":ti,ab,kw OR "Machupo mammarenavirus":ti,ab,kw OR nairovirus:ti,ab,kw OR "Crimean-congo hemorrhagic fever virus":ti,ab,kw OR CCHF*:ti,ab,kw OR "zaire ebolavirus":ti,ab,kw OR "omsk hemorrhagic fever virus":ti,ab,kw OR OHFV OR "kyasanur forest disease virus":ti,ab,kw OR "rift valley fever virus":ti,ab,kw OR RVF:ti,ab,kw OR [mh "Hemorrhagic Fevers, Viral"] OR [mh filoviridae] OR [mh "Hemorrhagic Fever, Ebola"] OR [mh ebolavirus] OR [mh "Marburg Virus Disease"] OR [mh "Lassa virus"] OR [mh "Lassa fever"] | 697 |
| #101 #1 AND #100 | 4 |
| #102 ( ("west nile" NEAR/2(fever OR virus)):ti,ab,kw) OR "West Nile flavivirus":ti,ab,kw OR "egypt 101 virus":ti,ab,kw OR "kunjin virus":ti,ab,kw OR WNV:ti,ab,kw OR [mh "West Nile Virus"] OR [mh "West Nile Fever"] | 43 |
| #103 #1 AND #102 | 0 |
| #104 "Yellow fever":ti,ab,kw OR YFV:ti,ab,kw OR [mh "Yellow fever"] OR [mh "Yellow fever virus"] | 73 |
| #105 #1 AND #104 | 0 |
| #106 Yersiniosis:ti,ab,kw OR "Yersinia enterocolitica":ti,ab,kw OR "Y. enterocolitica":ti,ab,kw OR "Y. pseudotuberculosis":ti,ab,kw OR [mh Yersinia] OR [mh "Yersinia infections"] | 12 |
| #107 #1 AND #106 | 0 |
| #108 Zika:ti,ab,kw OR Zikas:ti,ab,kw OR Zikv:ti,ab,kw OR Zikav:ti,ab,kw OR "congenial zika":ti,ab,kw OR [mh "zika virus"] OR [mh "zika virus infection"] | 86 |
| #109  #1 AND #108 | 1 |
| #110  #3 OR #5 OR #7 OR #9 OR #11 OR #13 OR #15 OR #17 OR #19 OR #21 OR #23 OR #25 OR #27 OR #29 OR #31 OR #33 OR #35 OR #37 OR #39 OR #41 OR #43 OR #45 OR #47 OR #49 OR #51 OR #53 OR #55 OR #57 OR #59 OR #61 OR #63 OR #65 OR #67 OR #69 OR #71 OR #73 OR #75 OR #77 OR #79 OR #81 OR #83 OR #85 OR #87 OR #91 OR #93 OR #95 OR #97 OR #99 OR #101 OR #103 OR #105 OR #107 OR #109 | 507 |
| **Apply Publication YEAR FILTER TO TRIALS (2014-2019) :** | **399** |

### AMR pathogen search (Cochrane)

This search was run on 22 November 2019.

| Search strings | No of hits |
| --- | --- |
| #1 ("Point of care test*" OR POCT OR "rapid diagnostic test*" OR "rapid test*" OR "bedside test*" OR "near patient test*" OR "handheld device*" OR "handheld instrument*" OR "portable test*" OR "portable device*" OR "portable instrument*" OR "bedside computing" OR "Infectious disease testing" OR "infectious disease screening*" OR "point of care technolog*" OR "bedside technolog*" OR "point of care system*"):ti,ab,kw OR ([mh "point-of-care systems"] OR [mh "point-of-care testing"]) | 1096 |
| #2 "Staphylococcus aureus":ti,ab,kw OR "micrococcus aureus":ti,ab,kw OR "microccus pyogenes":ti,ab,kw OR [mh Staphylococcus aureus] OR "Enterococcus faecium":ti,ab,kw OR "streptococcus faecium":ti,ab,kw OR [mh "Enterococcus faecium"] OR "Enterococcus faecalis":ti,ab,kw OR "streptococcus Group D":ti,ab,kw OR "streptococcus faecalis":ti,ab,kw OR "enterococcus fecalis":ti,ab,kw OR "enterococcus l form":ti,ab,kw OR "enterococcus proteiformis":ti,ab,kw OR "micrococcus ovalis":ti,ab,kw OR "micrococcus zymogenes":ti,ab,kw OR paraghurt:ti,ab,kw OR "streptococcus fecalis":ti,ab,kw OR "streptococcus glycerinaceus":ti,ab,kw OR "streptococcus liquefaciens":ti,ab,kw OR "streptococcus ovalis":ti,ab,kw OR "th 69":ti,ab,kw OR [mh "Enterococcus faecalis"] OR "Escherichia coli":ti,ab,kw OR "e coli":ti,ab,kw OR "e. coli":ti,ab,kw OR "Alkalescens-Dispar Group":ti,ab,kw OR EAggEC:ti,ab,kw OR "Bacillus coli":ti,ab,kw OR "Bacillus escherichii":ti,ab,kw OR "Bacterium coli":ti,ab,kw OR "bacterium E3":ti,ab,kw OR "coli bacillus":ti,ab,kw OR "coli bacterium":ti,ab,kw OR colibacillus:ti,ab,kw OR "colon bacillus":ti,ab,kw OR "Enterococcus coli":ti,ab,kw OR "Escherichia alkalescens dispart":ti,ab,kw OR [mh "Escherichia coli"] OR "Klebsiella pneumoniae":ti,ab,kw OR "Klebsiella rhinoscleromatis":ti,ab,kw OR "b. Friedlander":ti,ab,kw OR "bacillus pneumoniae":ti,ab,kw OR "Bacterium pneumoniae crouposae":ti,ab,kw OR "bacterium pneumonie crouposae":ti,ab,kw OR "friedlaender bacillus":ti,ab,kw OR "friedlander bacillus":ti,ab,kw OR "hyalococcus pneumoniae":ti,ab,kw OR "k. Pneumoniae":ti,ab,kw OR "klebsiella crouposa":ti,ab,kw OR "Klebsiella Pn":ti,ab,kw OR "klebsiella pneumonia":ti,ab,kw OR "Klebsiella pneumoniae aerogenes":ti,ab,kw OR pneumobacillus:ti,ab,kw OR [mh "Klebsiella pneumoniae"] OR "Acinetobacter baumannii":ti,ab,kw OR [mh "Acinetobacter baumannii"] OR [mh "Acinetobacter Infections"] OR "Pseudomonas aeruginosa":ti,ab,kw OR "Pseudomonas pyocyanea":ti,ab,kw OR "Bacillus aeruginosus":ti,ab,kw OR "Bacillus pyocyaneus":ti,ab,kw OR "Bacterium aeruginosum":ti,ab,kw OR "Bacterium pyocyaneum":ti,ab,kw OR "blue pus organism":ti,ab,kw OR "Micrococcus pyocyaneus":ti,ab,kw OR "P. Aeruginosa":ti,ab,kw OR "Pseudomonas polycolor":ti,ab,kw OR "Pseudomonas pyoceaneus":ti,ab,kw OR "Pseudomonas pyocyaneus":ti,ab,kw OR [mh "Pseudomonas aeruginosa"] OR "staphylococcus argenteus":ti,ab,kw | 4292 |
| #3  ("antimicrobial resistance" OR "antimicrobial testing" OR "Antimicrobial susceptibility" OR "Antibiotic susceptibility*" OR "antibiotic resistance" OR "antibiotic testing" OR "multi-drug resistant" OR '"multidrug-resistant" OR carbapenem-resistant OR carbapenemase OR "methicillin resistant" OR vanomycin-resistant OR "extended-spectrum beta-lactamase" OR ESBL OR AMR OR resistan* OR MDR OR XDR OR PDR OR "pandrug resistance" OR "pan-drug resistance"):ti,ab,kw OR [mh "Drug Resistance, Microbial"] OR [mh "Disease Resistance"] OR [mh "Drug Resistance, Bacterial"] | 43488 |
| #4  MeSH descriptor: [Drug Resistance] this term only | 354 |
| #5 #3 OR #4 | 43488 |
| #6 #1 AND #2 AND #5 | 8 |
| **Apply Publication YEAR FILTER TO TRIALS (2014-2019)** | 7 |

### Nosocomial pathogen search (Cochrane)

This search was run on the 22 November 2019.

| Search strings | No of hits |
| --- | --- |
| #1 ("Point of care test*" OR POCT OR "rapid diagnostic test*" OR "rapid test*" OR "bedside test*" OR "near patient test*" OR "handheld device*" OR "handheld instrument*" OR "portable test*" OR "portable device*" OR "portable instrument*" OR "bedside computing" OR "Infectious disease testing" OR "infectious disease screening*" OR "point of care technolog*" OR "bedside technolog*" OR "point of care system*"):ti,ab,kw OR ([mh "point-of-care systems"] OR [mh "point-of-care testing"]) | 1096 |
| #2 ("Streptococcus pneumoniae" OR Pneumococc* OR "Diplococcus pneumoniae" OR "Micrococcus pneumoniae"):ti,ab,kw OR [mh "Streptococcus pneumoniae"] OR [mh "Pneumococcal Infections"] OR ("Staphylococcus aureus" OR "micrococcus aureus" OR "microccus pyogenes"):ti,ab,kw OR [mh "Staphylococcus aureus"] OR ("Enterococcus faecium" OR "streptococcus faecium"):ti,ab,kw OR [mh "Enterococcus faecium"] OR "Enterococcus faecalis":ti,ab,kw OR "streptococcus Group D":ti,ab,kw OR "streptococcus faecalis":ti,ab,kw OR "enterococcus fecalis":ti,ab,kw OR "enterococcus l form":ti,ab,kw OR "enterococcus proteiformis":ti,ab,kw OR "micrococcus ovalis":ti,ab,kw OR "micrococcus zymogenes":ti,ab,kw OR paraghurt:ti,ab,kw OR "streptococcus fecalis":ti,ab,kw OR "streptococcus glycerinaceus":ti,ab,kw OR "streptococcus liquefaciens":ti,ab,kw OR "streptococcus ovalis":ti,ab,kw OR "th 69":ti,ab,kw OR [mh "Enterococcus faecalis"] OR "Escherichia coli":ti,ab,kw OR "e coli":ti,ab,kw OR "e. coli":ti,ab,kw OR "Alkalescens-Dispar Group":ti,ab,kw OR EAggEC:ti,ab,kw OR "Bacillus coli":ti,ab,kw OR "Bacillus escherichii":ti,ab,kw OR "Bacterium coli":ti,ab,kw OR "bacterium E3":ti,ab,kw OR "coli bacillus":ti,ab,kw OR "coli bacterium":ti,ab,kw OR colibacillus:ti,ab,kw OR "colon bacillus":ti,ab,kw OR "Enterococcus coli":ti,ab,kw OR "Escherichia alkalescens dispart":ti,ab,kw OR [mh "Escherichia coli"] OR "Klebsiella pneumoniae":ti,ab,kw OR "Klebsiella rhinoscleromatis":ti,ab,kw OR "b. Friedlander":ti,ab,kw OR "bacillus pneumoniae":ti,ab,kw OR "Bacterium pneumoniae crouposae":ti,ab,kw OR "bacterium pneumonie crouposae":ti,ab,kw OR "friedlaender bacillus":ti,ab,kw OR "friedlander bacillus":ti,ab,kw OR "hyalococcus pneumoniae":ti,ab,kw OR "k. Pneumoniae":ti,ab,kw OR "klebsiella crouposa":ti,ab,kw OR "Klebsiella Pn":ti,ab,kw OR "klebsiella pneumonia":ti,ab,kw OR "Klebsiella pneumoniae aerogenes":ti,ab,kw OR pneumobacillus:ti,ab,kw OR [mh "Klebsiella pneumoniae"] OR "Acinetobacter baumannii":ti,ab,kw OR [mh "Acinetobacter baumannii"] OR [mh "Acinetobacter Infections"] OR "Pseudomonas aeruginosa":ti,ab,kw OR "Pseudomonas pyocyanea":ti,ab,kw OR "Bacillus aeruginosus":ti,ab,kw OR "Bacillus pyocyaneus":ti,ab,kw OR "Bacterium aeruginosum":ti,ab,kw OR "Bacterium pyocyaneum":ti,ab,kw OR "blue pus organism":ti,ab,kw OR "Micrococcus pyocyaneus":ti,ab,kw OR "P. Aeruginosa":ti,ab,kw OR "Pseudomonas polycolor":ti,ab,kw OR "Pseudomonas pyoceaneus":ti,ab,kw OR "Pseudomonas pyocyaneus":ti,ab,kw OR [mh "Pseudomonas aeruginosa"] OR ("Clostridium difficile" OR "Clostridioides difficile" or "c diff" OR "c. diff" OR "c difficile" OR "c. difficile" OR "Pseudomembranous colitis"):ti,ab,kw OR [mh "Clostridium difficile"] OR [mh "Clostridium sordellii"] OR [mh "Clostridium perfringens"] OR (Candida OR "C. glabrata" OR "candida glabrata" OR "candida auris" OR Candidiasis OR Candidosis OR candidas OR monilia OR monilias OR "torulopsis utilis"):ti,ab,kw OR [mh Candida] OR [mh Candidiasis] OR "staphylococcus argenteus":ti,ab,kw | 7414 |
| #3 ("Nosocomial infection*" OR "Healthcare associated infection*" OR "healthcare acquired infection*" OR "Health care associated infection*" OR "health care acquired infection*" OR HCAI OR "Hospital acquired infection*" OR HAI OR "cross infection*" OR "hospital infection*"):ti,ab,kw OR [mh "Cross Infection"] | 1845 |
| #4 #1 AND #2 AND #3 | 3 |
| **Apply Publication YEAR FILTER TO TRIALS (2014-2019)** | 3 |

### General infectious disease search (Cochrane)

This search was run on the 22 November 2019.

| Search strings | No of hits |
| --- | --- |
| #1 ("Point of care test*" OR POCT OR "rapid diagnostic test*" OR "rapid test*" OR "bedside test*" OR "near patient test*" OR "handheld device*" OR "handheld instrument*" OR "portable test*" OR "portable device*" OR "portable instrument*" OR "bedside computing" OR "Infectious disease testing" OR "infectious disease screening*" OR "point of care technolog*" OR "bedside technolog*" OR "point of care system*"):ti,ab,kw OR ([mh "point-of-care systems"] OR [mh "point-of-care testing"]) | 1096 |
| #2 ("antimicrobial resistance" OR "antimicrobial testing" OR "Antimicrobial susceptibility" OR "Antibiotic susceptibility*" OR "antibiotic resistance" OR "antibiotic testing" OR "multi-drug resistant" OR '"multidrug-resistant" OR carbapenem-resistant OR carbapenemase OR "methicillin resistant" OR vanomycin-resistant OR "extended-spectrum beta-lactamase" OR ESBL OR AMR OR resistan* OR MDR OR XDR OR PDR OR "pandrug resistance" OR "pan-drug resistance"):ti,ab,kw OR [mh "Drug Resistance, Microbial"] OR [mh "Disease Resistance"] OR [mh "Drug Resistance, Bacterial"] OR ("Nosocomial infection*" OR "Healthcare associated infection*" OR "healthcare acquired infection*" OR "Health care associated infection*" OR "health care acquired infection*" OR HCAI OR "Hospital acquired infection*" OR HAI OR "cross infection*" OR "hospital infection*"):ti,ab,kw OR [mh "Cross Infection"] OR ("Infectious disease*" OR "Communicable disease*" OR infection*):ti,ab,kw OR [mh ”Communicable Diseases”] OR ("Sexually transmitted infection*" OR "Sexually transmitted disease*" OR STI OR STD OR "Venereal disease*"):ti,ab,kw OR [mh “Sexually Transmitted Diseases”] OR ("Respiratory infection*'" OR "Respiratory tract infection*" OR "Pleural Empyema*" OR "Thoracic Empyemas" OR Pyothorax OR "Tuberculous Empyema"):ti,ab,kw OR [mh "Respiratory Tract Infections"] OR "Gastrointestinal infection*":ti,ab,kw OR ("vaccine-preventable infection*" OR "vaccine-preventable disease*" OR VPD OR VPI):ti,ab,kw OR [mh "Vaccines"] OR ("Emerging infection*" OR "Emerging infectious disease*" OR "Emerging communicable disease*" OR "re-emerging communicable disease*" OR "reemerging communicable disease*" OR "reemerging infectious disease*" OR "re-emerging infectious disease*" OR "re-emerging infection*" OR "reemerging infection*"):ti,ab,kw OR [mh "Communicable Diseases, Emerging"] OR (Sepsis OR Septicemia OR Septicaemia OR "Septic shock" OR "Bloodstream infection*" OR "Blood infection*" OR Pyemia OR Pyemias OR Pyohemia OR Pyohemias OR Pyaemia OR Pyaemias OR "Blood Poisoning"):ti,ab,kw OR [mh Sepsis] OR ("Viral meningitis" OR "Bacterial meningitis" OR "Fungal meningitis" OR "Parasitic meningitis"):ti,ab,kw OR [mh "Meningitis, Viral"] OR [mh "Meningitis, Bacterial"] OR [mh "Meningitis, Fungal"] OR ("urinary tract infection*" OR UTI OR bacteriuria OR pyuria):ti,ab,kw OR [mh "urinary tract infections"] OR ("Bacterial pneumonia*" OR "viral pneumonia*" OR "fungal pneumonia*" OR "Infectious pneumonia*"):ti,ab,kw OR [mh "Pneumonia, Bacterial"] OR [mh "Pneumonia, Viral"] OR (diarrhoea OR diarrhea OR "diarrheal disease*" OR "diarrhoeal disease*"):ti,ab,kw OR [mh "Diarrhea"] | 127442 |
| #3 MeSH descriptor: [Drug Resistance] this term only | 354 |
| #4 #2 OR #3 | 127442 |
| #5 #1 AND #4 | 469 |
| **Apply Publication YEAR FILTER TO TRIALS (2014-2019)** | 383 |

### Cochrane search summary

| Search summary | Number of initial results (after de-duplication) | Number of unique results (after de-duplication) |
| --- | --- | --- |
| 56 Diseases | 399 | 73 |
| AMR pathogens | 7 | 1 |
| Nosocomial pathogens | 3 | 0 |
| General infectious diseases | 383 | 44 |
| **Total** | **792** | **118** |

## Summary of search results

Table 14: Summary of search results across the four databases

|  | PubMed | Embase | Scopus | Cochrane | Total |
| --- | --- | --- | --- | --- | --- |
| **Number of search hits before de-duplication of articles identified through more than one database** | | | | | |
| 56 Diseases | 3852 | 6150 | 5947 | 399 |  |
| AMR pathogens | 121 | 36 | 133 | 7 |  |
| Nosocomial pathogens | 33 | 18 | 60 | 3 |  |
| General infectious diseases | 4352 | 6362 | 7072 | 383 |  |
| **Total number of initial citations per database** | **8358** | **12566** | **13212** | **792** | **34928** |
| **Number of search hits after de-duplication of articles identified through more than one database** | | | | | |
| 56 Diseases | 3852 | 2710 | 1423 | 73 |  |
| AMR pathogens | 108 | 12 | 22 | 1 |  |
| Nosocomial pathogens | 13 | 4 | 11 | 0 |  |
| General infectious diseases | 1642 | 1261 | 822 | 44 |  |
| **Total number of unique citations per database** | **5345** | **3987** | **2278** | **118** | **11728** |

Total number of unique citations across the four databases: **11,728**

# Scoping review inclusion/exclusion criteria

| Criteria | Inclusion criteria | Exclusion criteria |
| --- | --- | --- |
| Population/topic of interest | Anthrax and its causative agent(s)  Avian influenza in humans and its causative agent(s)  Botulism and its causative agent(s)  Brucellosis and its causative agent(s)  Campylobacteriosis and its causative agent(s)  Chikungunya virus disease and its causative agent(s)  Chlamydia infections and its causative agent(s)  Cholera and its causative agent(s)  Cryptosporidiosis and its causative agent(s)  Dengue and its causative agent(s)  Diphtheria and its causative agent(s)  Echinococcosis and its causative agent(s)  Giardiasis and its causative agent(s)  Gonorrhoea and its causative agent(s)  Hepatitis A and its causative agent(s)  Hepatitis B and its causative agent(s)  Hepatitis C and its causative agent(s)  HIV infection and AIDS and its causative agent(s)  Infections with *Haemophilus influenzae* group B and its causative agent(s)  Influenza – including influenza A(H1N1) and its causative agent(s)  Invasive meningococcal disease and its causative agent(s)  Invasive pneumococcal disease and its causative agent(s)  Legionnaires’ disease and its causative agent(s)  Leptospirosis and its causative agent(s)  Listeriosis and its causative agent(s)  Lyme neuroborreliosis and its causative agent(s)  Malaria and its causative agent(s)  Measles and its causative agent(s)  Mumps and its causative agent(s)  Pertussis and its causative agent(s)  Plague and its causative agent(s)  Poliomyelitis and its causative agent(s)  Q fever and its causative agent(s)  Rabies and its causative agent(s)  Rubella and its causative agent(s)  Salmonellosis and its causative agent(s)  Severe Acute Respiratory Syndrome (SARS) and its causative agent(s)  Shiga-toxin/verocytotoxin-producing *Escherichia coli* (STEC/VTEC) infection and its causative agent(s)  Shigellosis and its causative agent(s)  Smallpox and its causative agent(s)  Syphilis and its causative agent(s)  Tetanus and its causative agent(s)  Tick borne encephalitis and its causative agent(s)  Toxoplasmosis, congenital and its causative agent(s)  Transmissible spongiform encephalopathies and its causative agent(s)  Trichinellosis and its causative agent(s)  Tuberculosis and its causative agent(s)  Typhoid and paratyphoid and its causative agent(s)  Tularaemia and its causative agent(s)  Variant Creutzfeldt–Jakob’s disease and its causative agent(s)  Viral haemorrhagic fever and its causative agent(s)  West Nile virus infection and its causative agent(s)  Yellow fever and its causative agent(s)  Yersiniosis and its causative agent(s)  Zika virus disease and its causative agent(s)  Congenital Zika virus disease and its causative agent(s)  *Streptococcus pneumoniae*  *Staphylococcus aureus*  *Enterococcus faecium*  *Enterococcus faecalis*  *Escherichia coli*  *Klebsiella pneumoniae*  *Acinetobacter baumannii complex*  *Pseudomonas aeruginosa*  *Clostridium difficile*  *Candida spp.*  *Staphylococcus argenteus* | Any other diseases/pathogens |
| Population/topic of interest | Studies conducted in EU/EEA countries | Studies conducted in non-EU/EEA countries |
| Intervention | Rapid (up to 90 minutes)  Portable or handheld (do not require laboratory infrastructure)  Delivered to end users  At least being tested in humans | Does not meet all the requirements for inclusion  Devices that are being developed but have not reached the stage of testing in humans |
| Comparison | Any or no comparison | N/A |
| Outcome | Communicable disease surveillance, prevention, and control | Uses of POCT other than for communicable disease surveillance, prevention, and control |
| Study | Peer reviewed article; article in press; conference paper; review; data papers; short survey; clinical trial (all human types); case reports; collected work; comparative study; congress; dataset; equivalence trial; evaluation studies; government document; guideline; historical article; interview; legal case; legislation; meta-analysis; multi-centre study; observational study; practice guideline; randomised control trial; Research Support, N.I.H., Extramural; Research Support, N.I.H., Intramural; Research Support, U.S. Government; systematic review; technical report; twin study | Conference abstract; conference review; editorials; erratum; letter; note; book or book chapter; business article or press; autobiography; bibliography; veterinary studies; dictionary; duplicate publication; expression of concern; Festschrift; interactive tutorial; Introductory Journal Article; lecture; news; newspaper article; overall; patient education handout; periodical index; personal narrative; portrait; publication components; publication formats; publication type category; Research Support; American Recovery and Reinvestment Act; Research Support, Non-U.S. Gov't; Research Support, U.S. Gov't, Non-P.H.S; Research Support, U.S. Gov't, P.H.S.; Scientific Integrity Review; study characteristics; support of research; validation studies; video-audio media; webcasts |
| Date | Published between 01 January 2014 and 15-21 November^[[4]](#footnote-4)^ 2019 | Published before 2014 |
| Language | English | Non-English |

# Scoping review extraction template

| **Citation** | **Include/exclude based on full-text. If exclude, stop here.** | **Study/document type** | **Study size** | **Study population** | **Study aims/objectives** | **Contributing countries** | **Communicable disease/pathogen and related health issue** |
| --- | --- | --- | --- | --- | --- | --- | --- |
|  |  |  |  |  |  |  |  |

| **Name of POCT device** | **Description of the POCT device** | **POCT only used for research recruitment? Yes/no** | **Turn around time** | **Study setting (i.e. hospital, home, primary care, community)** |
| --- | --- | --- | --- | --- |
|  |  |  |  |  |

| **Intended use** | **Intended use category** | **Actual use** | **Actual use category** | **Specificity range** | **Actual specificity** | **Sensitivity range** | **Actual sensitivity** | **Clinical impact** | **Other key findings** |
| --- | --- | --- | --- | --- | --- | --- | --- | --- | --- |
|  |  |  |  |  |  |  |  |  |  |

# Mapping exercise survey questions

**Introductory question**

1. Could you briefly describe your role and how it relates to POCT and infectious diseases?

**Availability of POCT devices and guidelines**

1. For the purpose of this study, we have defined a POCT device as a handheld or portable device that can provide a diagnostic result in 90 minutes or less and that is delivered to the end user (i.e. used near the patient rather than in a laboratory setting). Do you feel this definition captures the most important aspects of a POCT device?
   1. If yes, as there are differing views on what constitutes a POCT device, or do you think the majority of users and developers of these devices would agree with this definition?
   2. If no, what additional characteristics would a POCT device possess that we haven't covered?
2. To the best of your knowledge, for which infectious diseases is POCT commonly used for?
3. Is there variation across EU and EEA countries in the types of diseases POCT is used for, e.g. pathogen identification, antibiotic susceptibility testing?
4. Are there any specific settings or diseases in which the use of POCT device is widely implemented?
5. What specific public health functions is POCT used for in Europe?

**Use of POCT in clinical practice**

1. In general, are guidelines or recommendations available to clinicians for those diseases for which POCT are available for clinical use?
2. Is there variation across EU and EEA countries as to whether guidelines/recommendations are available to clinicians?
3. Are POCT devices routinely used in clinical practice for infectious diseases and related health issues?
   1. If yes: is the use of POCT for infectious diseases reimbursed?
4. Have you seen POCT replace traditional diagnostic techniques for any diseases?
   1. If yes, for which diseases?

**The impact of POCT devices**

9. What impact has the use of POCT devices had on patient-level activities, e.g. reporting test results?

10. What impact has the use of POCT devices had on public health-level activities, e.g. surveillance, infection/outbreak control?

# Replacement of traditional testing approaches by POCT

Number of diseases or related health issues for which POCT has replaced other tests for screening, triaging and diagnosis, by country and infectious disease

| Country | Number of diseases for which POCT has replaced other tests | Number of diseases for which POCT has not replaced other tests | Number of diseases for which responses indicated uncertainty | Total number of diseases for which responses to this question were received |
| --- | --- | --- | --- | --- |
|  | | | | |
| Country | | | | |
| Spain | 14 | 6 | 5 | 25 |
| Slovenia | 4 | 0 | 0 | 4 |
| Austria | 2 | 5 | 0 | 7 |
| Cyprus | 2 | 23 | 0 | 25 |
| Norway | 2 | 28 | 18 | 48 |
| Belgium | 1 | 2 | 1 | 4 |
| Denmark | 1 | 1 | 2 | 4 |
| Sweden | 1 | 3 | 1 | 5 |
| Bulgaria | 0 | 2 | 0 | 2 |
| Croatia | 0 | 6 | 0 | 6 |
| Estonia | 0 | 4 | 1 | 5 |
| Finland | 0 | 3 | 1 | 4 |
| France | 0 | 0 | 55 | 55 |
| Germany | 0 | 0 | 7 | 7 |
| Greece | 0 | 3 | 4 | 7 |
| Iceland | 0 | 1 | 0 | 1 |
| Ireland | 0 | 2 | 0 | 2 |
| Latvia | 0 | 4 | 0 | 4 |
| Liechtenstein | 0 | 0 | 0 | 0 |
| Lithuania | 0 | 0 | 0 | 0 |
| Malta | 0 | 6 | 0 | 6 |
| Netherlands | 0 | 5 | 0 | 5 |
| Poland | 0 | 1 | 0 | 1 |
| Romania | 0 | 4 | 0 | 4 |
| Slovakia | 0 | 2 | 0 | 2 |
| United Kingdom | 0 | 3 | 0 | 3 |
| Infectious diseases | | | | |
| Chlamydia infections | 2 | 3 | 3 | 8 |
| HIV infection and AIDS | 2 | 13 | 2 | 17 |
| Legionnaires’ disease | 2 | 9 | 2 | 13 |
| Antimicrobial resistance | 1 | 3 | 3 | 7 |
| Campylobacteriosis | 1 | 0 | 2 | 3 |
| Cholera | 1 | 0 | 2 | 3 |
| Cryptosporidiosis | 1 | 2 | 2 | 5 |
| Dengue | 1 | 1 | 3 | 5 |
| Giardiasis | 1 | 2 | 1 | 4 |
| Gonorrhoea | 1 | 2 | 2 | 5 |
| Hepatitis B | 1 | 5 | 1 | 7 |
| Hepatitis C | 1 | 5 | 1 | 7 |
| Influenza – including influenza A(H1N1) | 1 | 10 | 8 | 19 |
| Invasive meningococcal disease | 1 | 3 | 1 | 5 |
| Invasive pneumococcal disease | 1 | 5 | 1 | 7 |
| Nosocomial infections | 1 | 2 | 4 | 7 |
| Q fever | 1 | 0 | 1 | 2 |
| Salmonellosis | 1 | 0 | 2 | 3 |
| Shiga-toxin/verocytotoxin-producing *Escherichia coli* (STEC/VTEC) infection | 1 | 2 | 1 | 4 |
| Shigellosis | 1 | 1 | 1 | 3 |
| Tetanus | 1 | 0 | 1 | 2 |
| Typhoid and paratyphoid | 1 | 1 | 1 | 3 |
| Viral haemorrhagic fevers | 1 | 1 | 1 | 3 |
| Yersiniosis | 1 | 1 | 1 | 3 |
| Anthrax | 0 | 1 | 1 | 2 |
| Botulism | 0 | 1 | 1 | 2 |
| Brucellosis | 0 | 1 | 2 | 3 |
| Chikungunya virus disease | 0 | 1 | 1 | 2 |
| Diphtheria | 0 | 1 | 1 | 2 |
| Echinococcosis | 0 | 0 | 2 | 2 |
| Hepatitis A | 0 | 2 | 1 | 3 |
| Infections with *Haemophilus influenzae* group B | 0 | 1 | 3 | 4 |
| Leptospirosis | 0 | 1 | 1 | 2 |
| Listeriosis | 0 | 2 | 1 | 3 |
| Lyme neuroborreliosis | 0 | 1 | 2 | 3 |
| Malaria | 0 | 7 | 6 | 13 |
| Measles | 0 | 2 | 1 | 3 |
| Mumps | 0 | 2 | 1 | 3 |
| Pertussis | 0 | 1 | 1 | 2 |
| Plague | 0 | 0 | 2 | 2 |
| Poliomyelitis | 0 | 1 | 1 | 2 |
| Rabies | 0 | 0 | 1 | 1 |
| Rubella | 0 | 2 | 1 | 3 |
| Severe Acute Respiratory Syndrome (SARS) | 0 | 0 | 2 | 2 |
| Smallpox | 0 | 0 | 1 | 1 |
| Syphilis | 0 | 7 | 1 | 8 |
| Tick borne encephalitis | 0 | 0 | 2 | 2 |
| Toxoplasmosis, congenital | 0 | 1 | 2 | 3 |
| Transmissible spongiform encephalopathies | 0 | 0 | 1 | 1 |
| Trichinellosis | 0 | 0 | 1 | 1 |
| Tuberculosis | 0 | 5 | 2 | 7 |
| Tularaemia | 0 | 1 | 1 | 2 |
| Variant Creutzfeldt–Jakob’s disease | 0 | 0 | 1 | 1 |
| West Nile virus infection | 0 | 1 | 2 | 3 |
| Yellow fever | 0 | 0 | 1 | 1 |
| Zika virus disease | 0 | 1 | 1 | 2 |

**6. Glossary of POCT device names, associated tests, diseases covered and short description**^^[[5]](#footnote-5)^^

| POCT device name derived by research team | Examples of test names mentioned in studies | Disease/pathogen covered^[[6]](#footnote-6)^ | Description |
| --- | --- | --- | --- |
| ACON Chlamydia Rapid Test | ACON chlamydia, Acon Chlamydia/ Gonorrhoea Rapid Test | Chlamydia (*Chlamydia trachomatis*) and gonorrhoea | Immunochromatographic rapid test to diagnose either chlamydia or both chlamydia and gonorrhoea. |
| Actim Influenza A&B |  | Influenza A & B | Immunochromatographic assays to allow for the qualitative detection of influenza A and B nucleoprotein using a strip. The test is also able to distinguish between influenza A and B viruses. |
| Advanced Quality | Advanced, Advanced Quality Rapid Anti-HCV Test | Hepatitis C | Lateral flow test for hepatitis C virus using whole blood, plasma or serum. |
| Alere BinaxNOW | BinaxNOW, Alere BinaxNOW Influenza A & B card, Binax NOW malaria immune chromatography test | Influenza A & B, *Streptococcus pneumoniae*, malaria (*Plasmodium falciparum* and/or *Plasmodium vivax*/*Plasmodium ovale* spp./*Plasmodium malariae*), hepatitis B, pneumococcal infections | Immunochromatographic assays consisting of a strip that allows a qualitative detection and differentiation of a number of pathogens from urine and cerebrospinal fluid. The test detects nucleoproteins of influenza A and B, Histidine rich protein (HRP)-2 to detect *Plasmodium falciparum* and aldolase for other plasmodium species in malaria and membrane C-polysaccharide of *Streptococcus pneumoniae.* |
| Alere clearview | Clearview, Clearview Chlamydia, Clearview Exact Influenza A and B, Clearview HIV-1/2 STAT-PAK, Clearview Malaria Pf test, Clearview Strep A | Chlamydia (*Chlamydia trachomatis*), influenza A & B, malaria, group A Streptococcus, and HIV | Immunochromatographic test for chlamydia trachomatis, nucleoprotein of influenza A and B viruses and anti-HIV-1 and anti-HIV-2 antibodies. |
| Alere Determine | Determine, Alere Determine HIV 1/2 Ag/Ab Combo, Alere Determine Syphilis, Alere Determine TB LAM Ag Alere Determine HBsAgTM, Determine HIV 1/2, Determine QuickProfile VIKIA, Determine Combo | HIV, syphilis, TB, hepatitis B | Rapid immunochromatic blood, serum and urine test to detect antigens or antibodies related to a number of diseases. For HIV, HIV-1/2 and HIV-1 p24 antigen are detected, for syphilis, treponemal antibodies are detected and for TB, lipoarabinomannan (part of the cell wall) is detected. |
| Alere HIV combo | Alere HIV Combo, HIV 1/2 Ag/Ab Combo | HIV | Test to detect both HIV-1/2 antibodies and free HIV-1 p24 antigens using a strip in which the sample diffuses along and the result read in a window. |
| Alere i Influenza A & B | Alere and Influenza A&B, Alere I, Alere i Influenza A & B | Influenza A & B | Nucleic amplification test which uses an isothermal nicking-enzyme amplification method to qualitatively detect the nucleic acids of a number of pathogens, including: influenza A and B viruses, respiratory syncytial virus, and group A Streptococcus. |
| Alere Panbio Dengue Duo | Dengue Duo Rapid Test-SD, Panbio Dengue Duo Cassette, Panbio Dengue Early Rapid, Panbio Early Rapid NS1 and Duo Assay Kit, Alere Panbio Dengue Duo cassette for IgM/IgG analyte, Dengue Duo, Dengue NS1 Ag Strip | Dengue fever | Immunochromatographic assay able to detect non-structural protein 1 (NS1), IgM and IgG for dengue fever diagnosis. |
| Alere PIMA | Alere PIMA, Alere PIMA CD4, PIMA assay, PIMA | HIV | Image-based CD4 cell counting test for use on venous or capillary blood samples. Cartridges use beads to represent normal and low CD4 cell counts. |
| Alere Q | Alere q, Alere q HIV 1/2 Detect, Alere q platform, Alere Influenza A + B | Hepatitis C virus and HIV | Non-immunological, nucleic acid testing for hepatitis C virus, HIV-1 and HIV-2 Ribonucleic acid (RNA) detection to measure viral load. |
| Alere SD bioline | SD Bioline, SD Bioline Chikungunya IgM, SD Bioline Dengue Due NS1 and IgM/IgG Combo device, SD BIOLINE Dengue NS1, SD Bioline HBsAg, SD Bioline HCV, SD Bioline HIV/Syphilis Duo, SD Bioline HIV‐1/2 3.0, SD Bioline Influenza A/B/A(H1N1) Pandemic rapid test, SD Bioline Influenza Ag, SD Bioline Malaria Ag Pf/ Pan Rapid Diagnostic Test, SD Bioline Syphilis 3.0, SD BIOLINE Tetanus, SD Bioline Influenza Virus Antigen Test, SD Dengue IgG/IgM, SD Leptospira LF Standard Diagnostics | Hepatitis B, hepatitis C, syphilis, chikungunya virus disease, dengue fever, HIV, influenza A, B and H1N1, malaria, TB, tetanus and leptospirosis | Immunochromographic assay test using serum, plasma or whole blood to detect a range antigens and antibodies. The NS1 protein is detected for dengue, treponemal antibodies for syphilis, Gp41a, p24a, and Gp36a antigens for HIV, nucleoproteins of influenza A and B, hrp-2 and pan-LDH antigens for malaria, anti-IgM antibodies for leptospirosis. |
| aQcare Chlamydia TRF kit |  | Chlamydia | Rapid lateral flow assay using fluorescent nanoparticles for chlamydia |
| Architect HIV Ag/Ab Combo assay |  | HIV | Fourth generation rapid HIV test which detects HIV antigens and antibodies. |
| BD Directigen EZ Flu A+B |  | Influenza A & B | Immunochromatographic assays test to detect the nucleoproteins of influenza A and B and differentiate between the two viruses. |
| BD Veritor | BD Veritor, BD Veritor Flu A+B, BD Veritor Sytem for rapid detection of flu A+B, BD veritor Influenza A + B, BD veritor RSV, BD Veritor rapid diagnostic assays, Veritor System | Influenza A & B | Immunochromatographic assay to qualitatively detect the nucleoproteins of influenza A and B and respiratory syncytial virus and differentiate between the two viruses using nasopharyngeal aspirate samples. |
| BioNexia | bioNexia test, bioNexia Influenza A+B, bioNexia Strep A plus, Biopanda Toxo IgG/IgM | Influenza A and B, Legionnaires' disease (*Legionella pneumophila)*, toxoplasmosis (*Toxoplasma gondii*), group A streptococcus | Immunochromatographic assay to detect influenza A and B, *Legionella pneumophila*, *Toxoplasma gondii* and group A streptococcus. For Legionnaires' disease detection, *Legionella pneumophila* serogroup 1 is tested for in urine. For toxoplasmosis, IgG and IgM are detected from recombinant *Toxoplasma gondii* antigens. |
| BioRapid | BioRapid, BioRapid Chlamydia Ag test | Chlamydia (*Chlamydia trachomatis*) | Immunochromatographic test for *Chlamydia trachomatis*. |
| Biostar | BioStar Optical ImmunoAssay, BioStar Chlamydia, BioStar OIA Flu A/B | Chlamydia, influenza A and B, gonorrhoeae *(Neisseria gonorrhoeae)* | Optical immunoassay which allows visualisation of reactions between antigens and antibodies. |
| Biosynex CryptoPS |  | Cryptococcal meningitis | Lateral flow assay for detecting cryptococcal antigens. A qualitative band is shown in positive samples, with a second quantitative band if antigen levels are high. |
| CareStart malaria | CareStart Malaria HRP2 (Pf), CareStart Malaria HRP2/pLDH (Pf/PAN) Combo, CareStart malaria pLDH (PAN), CareStart malaria screen, CareStart Pf/Pv, CareStart Malaria | Malaria | Detection of HRP-2 and pLDH antigen for malaria diagnosis. |
| Chembio Dual Path Platform | Chembio DPP HIV-1/2 Assay, Chembio DPP Syphilis Screen & Confirm Assay, DPP HIV-Syphilis Assay, Dual Path Platform (DDP) Syphilis Test | Syphilis and HIV | Lateral flow test that can either detect syphilis (DDP test), or both syphilis and HIV (Chembio DDP test). For syphilis, antibodies against treponemal and non-treponemal antigens are detected. For HIV, anti-HIV-1 and anti-HIV-2 antibodies are detected. Whole blood, serum or plasma samples can be used. |
| Cobas Liat | Cobas Liat Influenza A/B, Cobas Liat influenza A/B & respiratory syncytial virus assay, Liat Cdiff Assay, Liat HIV Quant, The cobas Liat System, The LIAT Analyzer, Roche Cobas Liat platform | Influenza A & B, HIV, *Clostridioides difficile* | Rapid nucleic acid amplification test for influenza A and B and respiratory syncytial virus using nasopharyngeal samples. |
| Coris | Coris BioConcept Influ A&B Uni-Strip, Coris Crypto-Strip, Coris Duo-Strip | Influenza A and B and cryptosporidiosis (Cryptosporidium spp.) | Immunochromatographic tests for influenza A and B and cryptosporidiosis. |
| Directigen EZ Flu | Directigen EZ Flu A + B, Directigen Flu A | Influenza A & B | Commercially available POCT for influenza. |
| Enigma MiniLab | Enigma MiniLab, Enigma MiniLab FluAB-RSV PCR assay | Influenza A & B | Assay using nasopharyngeal samples to qualitatively detect influenza A (the matrix gene) and B (the non-structural gene) and respiratory syncytial virus (the fusion gene). |
| Espline Influenza A&B-N |  | Influenza A & B | Immunochromatographic assay using a strip to detect influenza A and B nucleoprotein. |
| FACSPresto |  | HIV | CD4 cell counting test using capillary or venous blood. |
| FilmArray | FilmArray, FilmArray Respiratory Panel, FilmArray Respiratory Panel kit version 1.6, FilmArray GI panels | Influenza A and B, Campylobacter spp. (*Campylobacter jejuni, Campylobacter coli*, and *Campylobacter upsaliensis*), *Clostridium difficile* (Toxin A/B), salmonella, *Yersinia enterocolitica*, Vibrio spp. (*Vibrio parahaemolyticus, vibrio vulnificus*, and *Vibrio cholerae*), enteroaggregative *Escherichia coli* (EAEC), enteropathogenic *Escherichia coli* (EPEC), enterotoxigenic *Escherichia coli* (ETEC), Shiga-like toxin-producing *Escherichia coli* (STEC), *Escherichia coli* O157, Shigella/enteroinvasive *Escherichia coli* (EIEC), cryptosporidiosis (Cryptosporidium  spp.), giardiasis (*Giardia lamblia*). | Reparatory version of the test detects 17 viruses and 3 bacteria, including influenza A, B and H1N1. Gastrointestinal version of the test detects: Campylobacter (*Campylobacter jejuni, Campylobacter coli*, and *Campylobacter upsaliensis*), *Clostridium difficile* (Toxin A/B), *Plesiomonas shigelloides*, salmonella, *Yersinia enterocolitica*, Vibrio spp. (*Vibrio parahaemolyticus, vibrio vulnificus*, and *Vibrio cholerae*), enteroaggregative *Escherichia coli* (EAEC), enteropathogenic *Escherichia coli* (EPEC), enterotoxigenic *Escherichia coli* (ETEC), Shiga-like toxin-producing *Escherichia coli* (STEC), *Escherichia coli* O157, Shigella/enteroinvasive *Escherichia coli* (EIEC), cryptosporidiosis (Cryptosporidium spp.), giardiasis (*Giardia lamblia*), *Cyclospora cayetanensis*, *Entamoeba histolytica*, adenovirus (F40/41, astrovirus, norovirus GI/GII, rotavirus A, and sapovirus (I, II, IV, and V). The tests integrate a number of different detection methods, including nucleic acid extraction, nested Polymerase chain reaction (PCR) and data analysis to identify a range of viral and bacterial nucleic acid targets in nasopharyngeal and stool samples. |
| GC | GC Check, GC One-step test, GC RapidResponse | Gonorrhoeae | No description provided. |
| Geenius | Geenius HIV 1/2Confirmatory Assay, Geenius rapid confirmatory test | HIV | Immunochromatographic test for detecting and differentiating between antibodies to HIV-1 and HIV-2. |
| HandiLab-C |  | Chlamydia | Enzyme detection test for chlamydia. |
| Hexagon | Hexagon chromatographic immunoanalysis, Hexagon HIV | HIV | Immunochromatographic test for detecting antibodies to HIV-1 and HIV-2. |
| illumigene Malaria | Illumigene Malaria, illumigene Malaria DNA Amplification assay | Malaria (*Plasmodium falciparum*, *Plasmodium vivax, Plasmodium ovale, Plasmodium malariae, and Plasmodium knowlesi*) | Qualitative loop-mediated isothermal amplification (LAMP) test for detecting the mitochondrial Deoxyribonucleic acid (DNA) of plasmodium species. Can detect *Plasmodium falciparum*, *Plasmodium vivax, Plasmodium ovale, Plasmodium malariae, and Plasmodium knowlesi.* |
| Immunocard Stat! | Immuno card Stat! Flu A&B, ImmunoCardSTAT!®CGE | Influenza A and B, giardiasis (*Giardia lamblia)*, cryptosporidiosis (Cryptosporidium spp.) | Immunochromatographic test for influenza A and B, *Giardia lamblia*, Cryptosporidium and *Entamoeba histolytica*. |
| Immunoflow | ImmunoFlow HCV test, ImmunoFlow HIV1‐HIV2 | Hepatitis C and HIV | Immunochromatographic cartridge test used for the qualitative detection of NS3, NS4 and NS5 antigens to identify hepatitis C and Gp120, gp41, and gp36b to identify HIV. |
| Influ | Influ A&B Respi Strip, Influ A&B Uni-Strip | Influenza A and B | Immunochromatographic assay using a strip which identifies and differentiates between influenza A and B nucleoproteins. |
| Influenza A/B 2 panel test |  | Influenza A and B | Immunochromatographic assay using a strip which identifies and differentiates between influenza A and B nucleoproteins. |
| INSTI | INSTI, INSTI HIV-1 Antibody test, INSTI HIV1/HIV 2 in vitro diagnostic test, INSTI HIV-1/HIV-2 Rapid Antibody Test, Insti Multiplex HIV-1/HIV-2/Syphilis Antibody Test | HIV and syphilis | Qualitative immunoassay that can either detect HIV or both HIV and syphilis. Detects HIV 1 and HIV-2 antigens and antibodies. |
| Labmen HCV test |  | Hepatitis C | Lateral flow test to detect anti-hepatitis C virus (HCV) antibodies in blood or oral fluid. |
| Legionella V Test |  | Legionella | Test for Legionella disease |
| Lepto | Leptocheck-WB Zephyr, LeptoTek Dri Dot, LeptoTek Lateral Flow, LeptoTek Lateral Flowa BioMerieux | Leptospirosis | Lateral flow assay to detect for anti-IgM antibody for leptospirosis detection in animals and humans. |
| mariPOC | mariPOC, MariPOC respi test | Influenza A and B, *Streptococcus pneumoniae* | Immunofluorescence-based Antigen test for nine respiratory viruses: influenza A and B; respiratory syncytial virus; parainfluenza virus types 1, 2 and 3; human metapneumovirus; human bocavirus; and adenovirus. The test also detects *Streptococcus pneumoniae*. |
| mChip |  | HIV | Miniature microfluidic–based enzyme-linked immunosorbent assay (ELISA) that detects gp41 and gp3 antibodies. |
| Multiplo | Multiplo, Multiplo Rapid TP/HIV Antibody Test | HIV and syphilis | Multiplo detects HIV and Multiplo Rapid *Treponema pallidum* (TP)/HIV Antibody Test detects both HIV and syphilis. Test based on immunofiltration methods to detect HIV and syphilis antigens and antibodies. |
| Multisure HCV |  | Hepatitis C | Lateral flow assay containing key antigens from HCV (core, NS3, NS4 and NS5). |
| OnSite | OnSite, OnSite Syphilis Ab Combo Rapid Test, OnSite Toxo IgG/IgM, OnSite Chikungunya  IgM Combo Rapid Test | Hepatitis C, syphilis, toxoplasma and chikungunya virus disease | Test card enclosed in a cassette in which the sample is pulled through by capillary action. Used to detect IgG and IgM from recombinant *Toxoplasma gondii* antigen and anti-HCV antibodies. |
| OptiMAL | OptiMAL and OptiMAL-IT | Malaria | Detect HRP-2 antigen for malaria detection. |
| Oraquick | Oraquick, Oraquick HCV rapid antibody test, Oraquick HBV Rapid Antibody Test, OraQuick Rapid HIV-1/2 Antibody Test, OraQuick assay, OraQuick In-Home HIV test, Orasure QuickFlu Rapid Flu A + B test | Hepatitis B, hepatitis C, HIV, Influenza A and B | Immunoassay for a range of viruses. For HCV detection, it identifies anti-HCV antibodies and for HIV it identified HIV-1 and HIV-2 antibodies. |
| Palutop | Palutop, PALUTOP+ 4 | Malaria | No description provided. |
| Parasight-F |  | Malaria | Detect HRP-2 antigen for malaria diagnosis. |
| PointCare NOW |  | HIV | CD4 cell counting device using flow cytometry to make CD4 cells fluorescent in blood samples so they can be counted by the test |
| Quick Navi-Flu |  | Influenza A and B | Immunochromatographic test for qualitative detection and differentiation of Influenza A and B nucleoprotein. |
| Quick Profile | Quick Profile, Quick Profile anti-HBsAb, QuickProfile HBV-3 Panel test | Hepatitis B | Rapid test to determine hepatitis B status. |
| Quickchek | Techlab Quik Chek, Giardia/Cryptosporidium Quik Chek | Giardiasis (*Giardia lamblia*) and cryptosporidium | Immunochromatographic test for *Giardia lamblia* and Cryptosporidium spp. |
| QuickVue | QuickVue, QuickVue Influenza A + B test, QuickVue Chlamydia Rapid Test, RIDT Quickvue | Chlamydia (*Chlamydia trachomatis*), influenza A and B | Immunochromatographic test for chlamydia, influenza A and B and Group A strep |
| Quidel | Quidel QuickVue Influenza A+B, Quidel Sofia influenza A and B flouroescent immunoassay, Quidel QuickVue Influenza A + B | Influenza A and B | Lateral flow immunoassay using immunofluorescence technology. The test can quantitively detect influenza A and B nucleoproteins. |
| ReEBOV antigen rapid test |  | Ebola virus disease | No description provided. |
| RIDA | R-Biopharm RIDA QUICK CG Combi, R-Biopharm RIDA®QUICK Cryptosporidium, RIDA®QUICK Cryptosporidium/Giardia/Entamoeba Combi, RIDAGENE Flu & RSV kit | Cryptosporidiosis (Cryptosporidium spp.), giardiasis, influenza A and B | Immunochromatographic tests for detecting a range of pathogens. Other tests are available to detect norovirus and adeno rotavirus. |
| SAMBA | SAMBA, SAMBA II, SAMBA HIV Semiquantitative Test, SAMBA HIV-1 qualitative assay, SAMBA I HIV-1 Qual whole blood test, SAMBA II WBSQ | HIV | Semiquantitative assay which amplifies HIV-1 nucleic acid (from groups M, N and O) and produces a blue band if HIV is present in the blood sample. The quantitative test is able to distinguish between patients with viral loads above and below 1000 copies/ml. |
| Simplexa Flu A/B & RSV kit |  | Influenza A and B | Detects influenza A and B and RSV |
| Sofia Influenza A+ B | Sofia Influenza A+ B, Sofia immunoassay, Sofia fluorescent immunoassay, Sofia, The Sofia Fluorescent Immunoassay Analyzer | Influenza A and B | Lateral flow immunoassays to detect influenza A and B and RSV nucleoprotein antigens. The test demonstrates a positive result using a fluorescent reaction. |
| Syphicheck | Syphicheck, Syphicheck (qualpro), Syphicheck-WB | Syphilis | Test card enclosed in a cassette to detect syphilis in whole blood, serum or plasma |
| Syphilis Health Check |  | Syphilis | Qualitative immunochromatographic assay for the detection of *Treponema pallidum* antibodies for syphilis diagnosis. |
| TOYO | TOYO anti-HCV test, Toyo HBsAg rapid test | Hepatitis B and hepatitis C | Lateral flow test to detect hepatitis B and C viruses in blood and oral fluid |
| Truenat | Truenat, TrueNat HIV Viral Load, Truenat Uno | TB, HIV and malaria (*Plasmodium falciparum* and *Plasmodium vivax*) | Nucleic acid amplification using PCR to detect TB from sputum samples and HIV and malaria from blood samples. |
| Uni-gold | Uni-Gold Syphilis Treponemal, Uni-Gold Streptococcus pneumoniae | Syphilis and *Streptococcus pneumoniae* | Lateral flow immunoassay in a cassette to qualitatively detect syphilis and pneumococcal soluble antigens using blood, plasma and serum (for syphilis) or cerebrospinal fluid (CSF) or urine (for *Streptococcus pneumoniae*). |
| VIKIA | VIKIA, VIKIA HIV, VIKIA BioMérieux, VIKIA HBsAg, VIKIA HIV 1/2 | HIV, hepatitis B | Lateral flow test that detects anti-HIV and anti-hepatitis B virus (HBV) antibodies. |
| VIRapid HYDATIDOSI |  | Cystic echinococcosis | Immunochromatographic test based on purified antigen B and antigen 5 of human cystic echinococcosis |
| Visitect | Visitect, Visitect CD4, Visitect syphilis | Syphilis and HIV | ELISA test to detect syphilis and CD4 protein on T cells to determine if T lymphocytes are above or below a threshold in HIV. |
| Xpert | Cepheid Gene Xpert, Cepheid Xpert CT/NG, Cepheid Xpert Flu Assay, Cepheid Xpert GBS system, Cepheid Xpert HIV-1 Viral Load, Cepheid Xpert Influenza+RSV Xpress Assay, Cepheid Xpert MRSA, GeneXpert R MTB/Rif Ultra assay, GeneXpert Omni, Xpert Flu A & B, Xpert Carba-R assay, Xpert HCV Viral Load test, Xpert GBS molecular test, Xpert molecular assay, Xpert MRB/RIF, XpertMRSA - G3 version, XpertMRSA - NxG version | Influenza A and B, Clostridium difficile, *Chlamydia trachomatis*, *Neisseria gonorrhoeae*, HIV, Methicillin resistant *Staphylococcus aureus* (MRSA), TB, hepatitis C, *Staphylococcus aureus*, group B streptococcus | Real-time PCR nucleic acid amplification test for a number of pathogens. In *Clostridium difficile* infections, the genes for toxin B (tcdB), binary toxin (cdt) and a point mutation associated with PCR ribotype are detected. In chlamydia and gonorrhoea diagnosis, the DNA of the bacteria is detected from vaginal or urine swabs. For HIV diagnosis, the viruses RNA and DNA are detected. In hepatitis C, the viruses RNA is detected. When using the test for TB, it can be used to both detect the presence of TB and identify if the TB will be resistant to rifampicin treatment. For influenza, the test can detect and differentiate influenza A (including H1N1) and B, with some also able to detect RSV. |
| ZIKV | ZIKV NASBA, ZIKV RT-RPA assay | Zika virus disease | Recombinase polymerase amplification assay to detect Zika virus nucleic acid. |

1. After running this search on the 15 November, we identified a typo in this search string relating to *Klebs-Loeffler bacillus.* We re-ran the search on the 12 December with the correct spelling of this term and did not identify any additional search hits. [↑](#footnote-ref-1)
2. After running this search on the 15 November, we identified an error in the string relating to the epidemic parotid virus terms, in that PubMed cannot search truncations (*) inside of quotation marks. We created a correct search string (Epidemic[tw] AND virus*[tw] AND (parotid[tw] OR parotiditis[tw] OR parotitides[tw] OR parotitus[tw])) and re-ran this search on 12 December, however the number of search hits remained the same. [↑](#footnote-ref-2)
3. De-duplication occurred after each type of search was run in each database, hence the number of unique citations reduces from PubMed to Cochrane. The searches are in the order in which they were conducted across each database, with the 56 disease search conducted in PubMed being the first search, and the general infectious disease in Cochrane being the last. The results presented in these annexes are in the order in which they were searched. [↑](#footnote-ref-3)
4. The searches of the four databases were conducted on different days in November which is why this date ranges across multiple days. [↑](#footnote-ref-4)
5. This includes only those tests mentioned in 2 or more separate studies. Where multiple diseases are covered by the test, this does not necessarily mean one test detects all diseases. Rather, there may be multiple types of the same named test. The diseases named here are those relevant to the scoping review; the tests may also detect pathogens not included in this scoping review. [↑](#footnote-ref-5)
6. Where included in the studies, both the pathogen and disease name are provided. [↑](#footnote-ref-6)
